# Design of PSMA ligands with modifications at the inhibitor part: an approach to reduce the salivary gland uptake of radiolabeled PSMA inhibitors?

**DOI:** 10.1186/s41181-021-00124-1

**Published:** 2021-02-26

**Authors:** Veronika Barbara Felber, Manuel Amando Valentin, Hans-Jürgen Wester

**Affiliations:** Technical University of Munich, Chair of Pharmaceutical Radiochemistry, Walther-Meißner-Str. 3, 85748 Garching, Germany

**Keywords:** PSMA, GCP II, Prostate cancer, Radioligand therapy, Salivary glands

## Abstract

**Aim:**

To investigate whether modifications of prostate-specific membrane antigen (PSMA)-targeted radiolabeled urea-based inhibitors could reduce salivary gland uptake and thus improve tumor-to-salivary gland ratios, several analogs of a high affinity PSMA ligand were synthesized and evaluated in in vitro and in vivo studies.

**Methods:**

Binding motifs were synthesized ‘on-resin’ or, when not practicable, in solution. Peptide chain elongations were performed according to optimized standard protocols via solid-phase peptide synthesis. In vitro experiments were performed using PSMA^+^ LNCaP cells. In vivo studies as well as *μ*SPECT/CT scans were conducted with male LNCaP tumor xenograft-bearing CB17-SCID mice.

**Results:**

PSMA ligands with A) modifications within the central Zn^2+^-binding unit, B) proinhibitor motifs and C) substituents & bioisosteres of the P1′-γ-carboxylic acid were synthesized and evaluated. Modifications within the central Zn^2+^-binding unit of PSMA-10 (Glu-urea-Glu) provided three compounds. Thereof, only ^nat^Lu-carbamate I (^nat^Lu-**3**) exhibited high affinity (IC_50_ = 7.1 ± 0.7 nM), but low tumor uptake (5.31 ± 0.94% ID/g, 1 h p.i. and 1.20 ± 0.55% ID/g, 24 h p.i.). All proinhibitor motif-based ligands (three in total) exhibited low binding affinities (> 1 μM), no notable internalization and very low tumor uptake (< 0.50% ID/g). In addition, four compounds with P1′-ɣ-carboxylate substituents were developed and evaluated. Thereof, only tetrazole derivative ^nat^Lu-**11** revealed high affinity (IC_50_ = 16.4 ± 3.8 nM), but also this inhibitor showed low tumor uptake (3.40 ± 0.63% ID/g, 1 h p.i. and 0.68 ± 0.16% ID/g, 24 h p.i.). Salivary gland uptake in mice remained at an equally low level for all compounds (between 0.02 ± 0.00% ID/g and 0.09 ± 0.03% ID/g), wherefore apparent tumor-to-submandibular gland and tumor-to-parotid gland ratios for the modified peptides were distinctly lower (factor 8–45) than for [^177^Lu]Lu-PSMA-10 at 24 h p.i.

**Conclusions:**

The investigated compounds could not compete with the in vivo characteristics of the EuE-based PSMA inhibitor [^177^Lu]Lu-PSMA-10. Although two derivatives (**3** and **11**) were found to exhibit high affinities towards LNCaP cells, tumor uptake at 24 h p.i. was considerably low, while uptake in salivary glands remained unaffected. Optimization of the established animal model should be envisaged to enable a clear identification of PSMA-targeting radioligands with improved tumor-to-salivary gland ratios in future studies.

**Supplementary Information:**

The online version contains supplementary material available at 10.1186/s41181-021-00124-1.

## Background

For imaging of prostate cancer (PCa) lesions, a variety of small molecule-based prostate-specific membrane antigen (PSMA) ligands have been or are currently investigated in clinical trials (Cimadamore et al. 2018; https://clinicaltrials.gov/ct2/results?cond=Prostate+Cancer&term=PSMA&cntry=&state=&city=&dist= 2020). Labeled with ^18^F, ^68^Ga, ^99m^Tc or ^123^I these compounds show high potential for visualization of primary tumors and metastases (Rowe et al. 2015; Giesel et al. 2018; Afshar-Oromieh et al. 2015; Weineisen et al. 2015; Vallabhajosula et al. 2014; Barrett et al. 2013). Additionally, ^64^Cu- or ^44^Sc-labeled PSMA ligands have proved their usefulness for prolonged acquisition periods, as required for pre-therapeutic dosimetry or intraoperative applications as well as for images with higher spatial resolution (Zhou et al. 2019; Eppard et al. 2017). Transfer of this targeted concept to radioligand therapy (RLT) of metastatic castration-resistant prostate cancer (mCRPC) by means of β^−^-emitting ligands, such as [^177^Lu]Lu-PSMA-I&T (Weineisen et al. 2015) and ^177^Lu-PSMA-617 (Benesova et al. 2015), already resulted in highly promising clinical studies (Rahbar et al. 2017). [^177^Lu]Lu-PSMA-617 is currently evaluated in a Phase III study (https://clinicaltrials.gov/ct2/show/NCT03511664?term=PSMA&cond=Prostate+Cancer&phase=2&draw=5&rank=2 2020) and moreover, PSMA-targeted α-therapy was introduced as a further salvage therapy of end-stage mCRPC (Chakravarty et al. 2018). The first application of ^225^Ac-labeled PSMA-617 in humans was reported in 2016 by Kratochwil et al. (Kratochwil et al. 2016a).

In particular, radiolabeled small molecule PSMA inhibitors, based on the L-Glu-urea-X binding motif have been shown to exhibit noticeable uptake in kidneys (proximal tubules), lacrimal and salivary glands (Zechmann et al. 2014; Kratochwil et al. 2016b; Klein Nulent et al. 2018; Oh et al. 2019). Elevated activity accumulation in these organs can be partially assigned to endogenous PSMA expression, but also to a non-PSMA-related uptake mechanism, as PSMA expression was assumed to be present to a considerable lower extent than on the surface of PCa (Ghosh and Heston 2004; Rupp et al. 2019). Thus, beside kidneys, the lacrimal and salivary glands have been identified as critical organs (Tönnesmann et al. 2019; Kratochwil et al. 2017). In this context it has become apparent, that ^177^Lu-labeled low molecular weight PSMA inhibitors can cause xerostomia (Kratochwil et al. 2017; Kratochwil et al. 2018), which can affect patients’ quality of life and was found to be partially irreversible when α-emitter-labeled (^225^Ac) compounds were administered (Rupp et al. 2019; Kratochwil et al. 2018; van Kalmthout et al. 2019). Hence, for the future optimization of PSMA-targeted radioligand therapies, the development of strategies to design improved small molecule-based PSMA ligands with reduced salivary gland uptake is of high priority.

This working hypothesis presupposes a specific uptake of the aforementioned PSMA inhibitors in salivary glands, although not necessarily PSMA-specific. Whereas PSMA protein and mRNA expression in salivary glands was confirmed by Western blot and genetic analyses (Troyer et al. 1995; Israeli et al. 1994; O'Keefe et al. 2004), the high accumulation of radiolabeled small molecule-based ligands (e.g. [^177^Lu]Lu-PSMA-617 and [^68^Ga]Ga-PSMA-11) did not correlate with the rather low PSMA expression density detected by immunohistochemistry and in patients, treated with the radiolabeled antibody [^177^Lu]Lu-J591 (Rupp et al. 2019; Horoszewicz et al. 1987; Lopes et al. 1990; Bander et al. 2005). Indeed, unwanted non-target tissue uptake in salivary glands is markedly reduced using radiolabeled antibodies. Anyhow, therapeutic concepts using PSMA targeting antibodies are affected by slow diffusion into solid lesions and myelotoxicity due to longer blood circulation (Bander et al. 2005; Maurer et al. 2016; Tagawa et al. 2013).

Based on the fact that administration of monosodium glutamate in mice prior to [^68^Ga]Ga-PSMA-11 markedly reduced activity uptake in salivary glands whilst maintaining high tumor uptake, non-PSMA specific interactions such as small molecule/anion/glutamate transporter mechanisms may be conceivable (Rousseau et al. 2018). However, the detailed mechanism of uptake into salivary and lacrimal glands and the respective target structure has not been elucidated so far (Rupp et al. 2019; Rousseau et al. 2018).

This lack of knowledge led to various different approaches towards the reduction of radioactivity uptake into the salivary glands and thus corresponding unwanted side effects during radioligand therapy. After reduction of stimulus conduction by injection of botulinum toxin, a significant decrease of the SUVmean (up to 64%) in the right parotid gland compared to the left (control) was observed (Baum et al. 2018). In contrast, external cooling with ice packs to decrease the overall blood perfusion showed no effect on ligand uptake and xerostomia when ^177^Lu-labeled peptides were used (van Kalmthout et al. 2018; Rathke et al. 2019; Yilmaz et al. 2019). Furthermore, excretion stimulus via vitamin C was investigated, but did not lead to any measurable uptake reduction of [^68^Ga]Ga-PSMA-11 (Afshar-Oromieh et al. 2015). Sialendoscopy with dilatation, saline irrigation and steroid injection (prednisolone) after targeted α-therapy ([^225^Ac]Ac-PSMA-617) showed beneficial effects on salivary gland function preservation and for patients‘ quality of life (Rathke et al. 2019). However, not all radiation induced damages on the parenchyma could be avoided, as macroscopic findings during sialendoscopy revealed endothelial avascularity with the presence of stenosis (Rathke et al. 2019). All in all, those efforts did not show the desired efficacy.

**Fig. 1:**
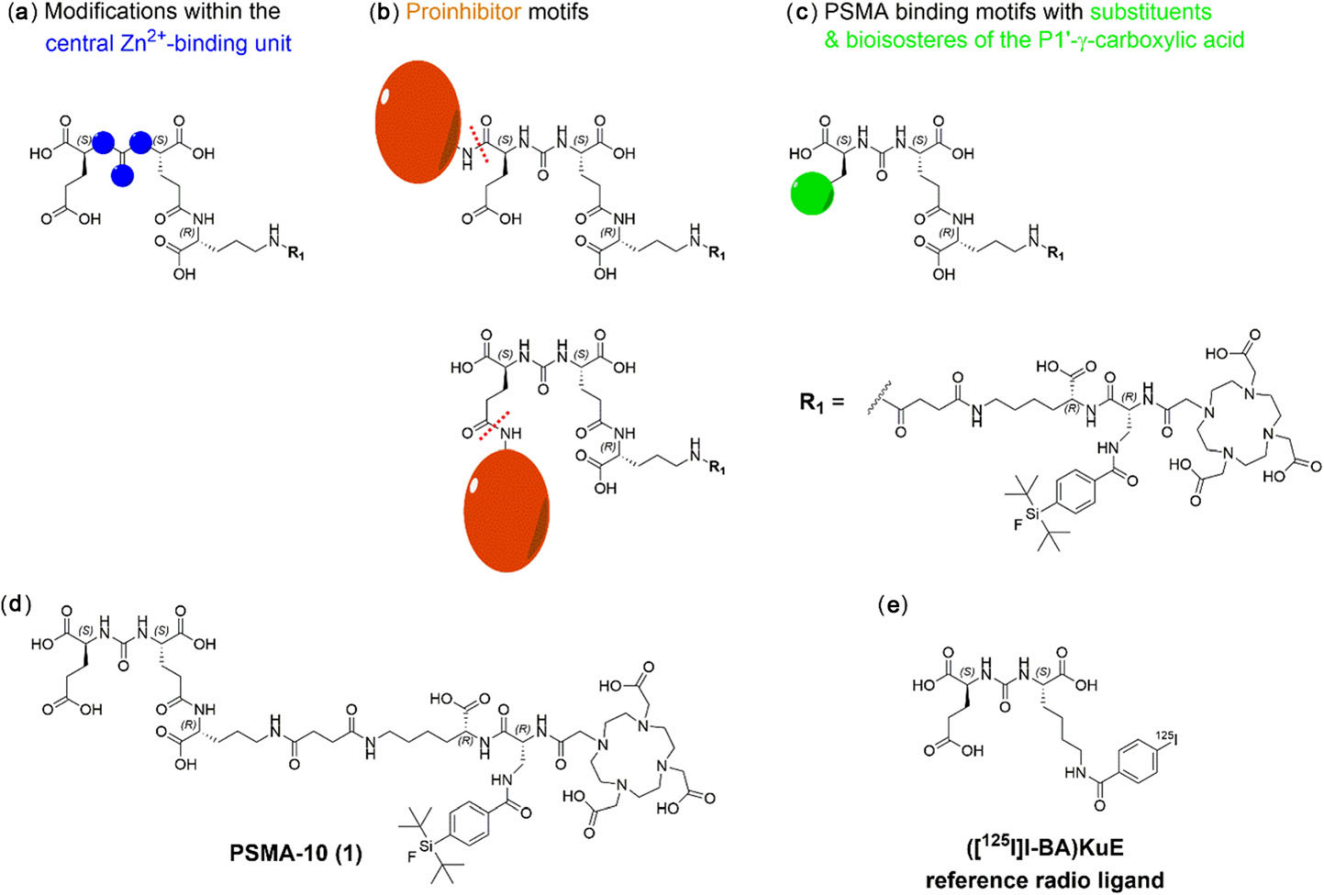
Schematic representation of PSMA inhibitors containing (**a**) modifications within the central Zn^2+^-binding unit (**b**) proinhibitor motifs (expected cleavage sites are indicated as red dotted lines) and (**c**) substituents & bioisosteres of the P1’-γ-carboxylic acid. All compounds were derived from the EuE-based ligand PSMA-10 (**1**) (**d**) which served as a reference for all obtained in vitro and in vivo data. **e** The reference ligand for IC_50_ determinations was ([^125^I]I-BA)KuE

With the aim to investigate whether modifications at the urea-based inhibitor unit could reduce the non-PSMA-specific or perhaps ‘transporter-mediated’ uptake (Kratochwil et al. 2017; Rousseau et al. 2018) of PSMA-binding ligands into salivary glands, three structural alterations were investigated and evaluated (Fig. 1):

A) Modifications within the central Zn^2+^-binding unit
B) Proinhibitor motifs
C) PSMA-binding motifs with substituents & bioisosteres of the P1’-γ-carboxylic acid

### Rationales for implemented inhibitor modifications

#### Modifications within the central Zn^2+^-binding unit

The *C*-terminal glutamate represents a common feature of both phosphoramidate- and urea-based PSMA ligands. However, the absorbed dose in salivary glands of PCa patients could be decreased by a factor of ~ 4 via administering a phosphoramidate-based PET agent compared to the urea-based radiotracer [^68^Ga]Ga-PSMA-I&T (Behr et al. 2019; Herrmann et al. 2015). Therefore, inhibitors containing modifications within the Zn^2+^-binding unit were synthesized, assuming that fragments of the Zn^2+^-binding group might serve as potential recognition sites for small molecule/anion/glutamate transporter systems or other transporter-independent uptake mechanisms.

Based on the results of Rousseau et al. (Rousseau et al. 2018), the *C*-terminal glutamate of PSMA ligands was presumed to cause for unwanted uptake into salivary glands. In currently used EuE- and KuE-based PSMA inhibitors, this structural feature is set as an essential part for proper ligand binding. However, at the same time it represents the fragment which is most similar to monosodium glutamate, by which activity accumulation could be markedly reduced in salivary glands in mice if administered 15 min prior to [^68^Ga]Ga-PSMA-11. Therefore, masking of this ligand-inherent glutamate was tried to realize by proinhibitors (reversible masking) and PSMA ligands with substituents & bioisosteres of the P1’-γ-carboxylic acid (irreversible masking).

#### Proinhibitors

Prodrug approaches for targeted delivery of cytotoxic agents to PCa lesions have been recently published and were further evaluated in an open label dose-escalation trial (NCT01056029) (Denmeade et al. 2012; Ristau et al. 2014). Hence, this prodrug principle was considered as a reasonable option for reducing non-target tissue uptake of PSMA ligands. Thereby, PSMA as hydrolytical active enzyme could act as instrument for on-site liberation of the respective inhibitor and yet serve as the target for RLT of PCa. Non-target tissue accumulation would thus be reduced to a remarkably low level, as binding of the masked inhibitor is only possible to enzymatically active sites. Variations from the original PSMA substrate *N-*acetyl-L-aspartyl-L-glutamate (NAAG), were recently published by Barinka et al. and Plechanovová et al. (Barinka et al. 2002; Plechanovova et al. 2011) Thereof, Ac-L-Glu-L-Met and Ac-L-Asp-L-2-Aoc showed turnover numbers (k_cat_) and cleavage efficiencies (k_cat_/K_m_) closest to the respective values indicated for NAAG. For this reason, both glutamate surrogates, L-Met as well as L-2-Aoc were implemented into the existing structure of PSMA-10 (**1**). They were linked to the α- or γ-carboxylate of the *C*-terminal glutamate, as the preferred cleavage site could not be clearly specified upfront. Hydrolysis might occur between α-linkages (NAALADase activity of GCP II in the central and peripheral nervous system) or between γ-linkages (FOLH1 activity in the gastrointestinal tract) (Barinka et al. 2012). In addition, prodrug approaches focusing just on γ-linkages were adversely affected by metabolic instability in human plasma (Mhaka et al. 2004).

#### PSMA-binding motifs with substituents & bioisosteres of the P1’-γ-carboxylic acid

Modifications at the P1’-γ-carboxylic acid moiety were preferred, as previous studies conducted by Kozikowski et al. revealed substitutions at the P1’ glutamate to be more tolerated at the γ-carboxylate than at the α-carboxylate (Kozikowski et al. 2004). Moreover, the P1’-γ-carboxylic acid possibly acts as the relevant recognition site for small molecule/anion/glutamate transporter systems (Rousseau et al. 2018). Besides Kozikowski et al., Wang et al. and Plechanovová et al. investigated the effect of carboxylic acid bioisosteres and aliphatic substituents on the affinity of urea-based inhibitors towards PSMA (Plechanovova et al. 2011; Wang et al. 2010). On the basis of these studies, incorporation of the most auspicious carboxylic acid substituents was pursued.

All these modifications were investigated based on one of our recently developed PSMA ligands, PSMA-10 (**1**) (Wurzer et al. 2020). In particular, a clear distinction between the salivary gland uptake values of EuE- and non-EuE-based radioligands and higher tumor-to-salivary gland ratios at 24 h p.i. in comparison to EuE-based PSMA-10 (**1**) were of primary interest. The latter might indicate for a possible transferability to humans, despite certain species-dependent differences, i.e. a general lower uptake in mouse salivary glands (Knedlik et al. 2017; Roy et al. 2020).

## Methods

For detailed information on all methods for synthesis and analysis as well as on the used instruments, see the ‘GENERAL INFORMATION’, ‘MATERIALS’ and ‘METHODS’ section in the supporting information (supplemental materials are available on https://ejnmmipharmchem.springeropen.com).

### Chemical synthesis

**Fig. 2:**
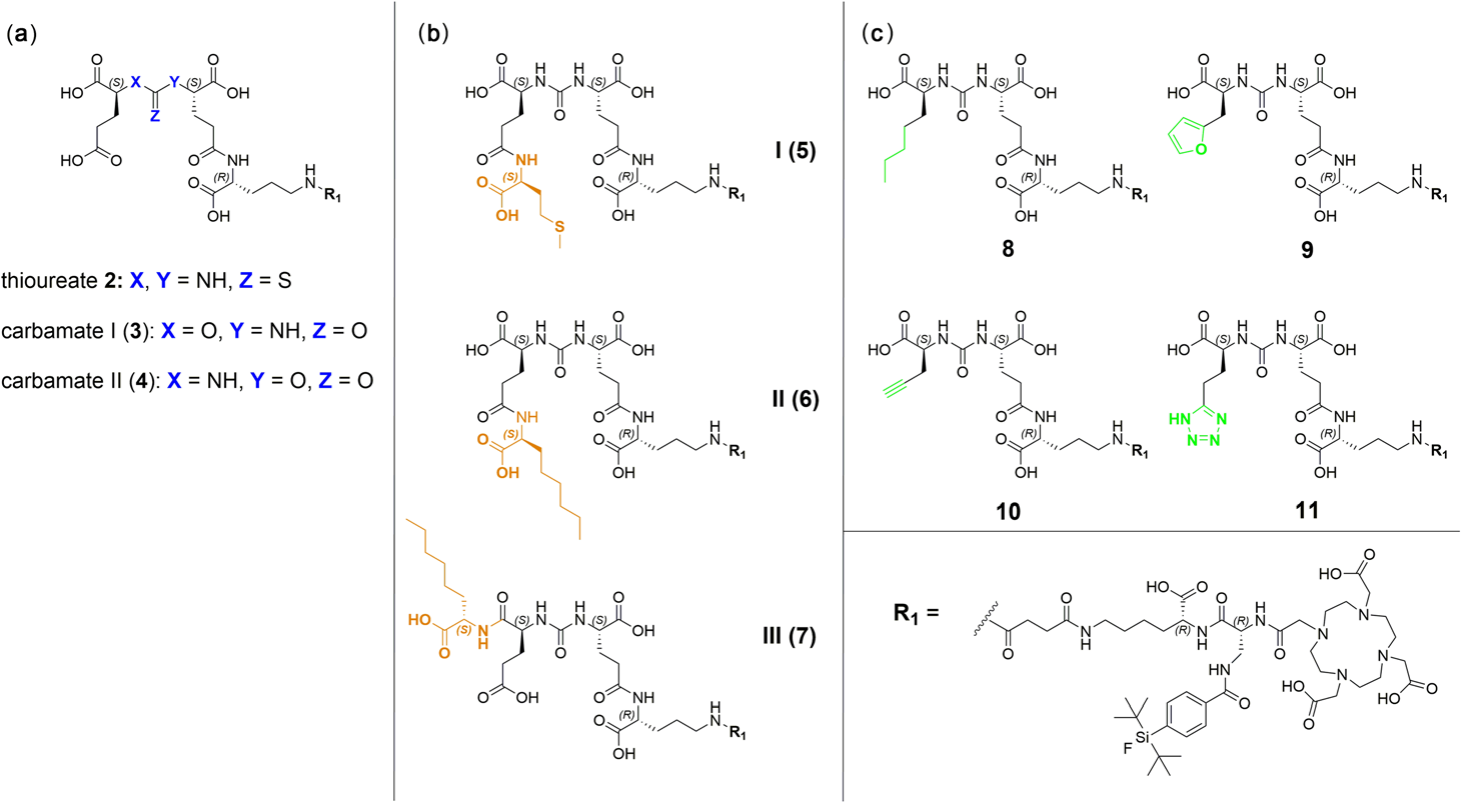
Detailed structures of the modified PSMA inhibitors investigated in this study, with (**a**) thioureate **2**, carbamate I & II (**3** & **4**) (**b**) proinhibitors I, II and III (**5**, **6** & **7**) and (**c**) L-2-aminoheptanoic acid (**8**), furyl (**9**), alkyne (**10**) and tetrazole (**11**) derivatives. All compounds are depicted in their free chelator form and represent PSMA ligands containing modifications within the central Zn^2+^-binding unit (**a**), proinhibitor motifs (**b**) and substituents & bioisosteres of the P1’-γ-carboxylic acid (**c**)

The PSMA ligands were prepared via a mixed solid phase/solution-phase synthetic strategy. Final purification of the compounds was achieved by RP-HPLC. For a detailed description of the synthesis of PSMA derivatives **1–11** (free chelator forms) see the ‘METHODS’ section in the supporting information. Detailed ^nat^Ga- and ^nat^Lu-complexation procedures as well as characterization of the resulting compounds are also provided in the ‘METHODS’ section in the supporting information. Schematic illustrations of all PSMA derivatives (**1–11**) as well as the structural formula of the reference ligand (((*S*)-1-carboxy-5-(4-(^125^Iodo) benzamido)pentyl)carbamoyl)-L-glutamic acid (([^125^I]I-BA)KuE) are depicted in Figs. 1 and 2.

### Radiolabeling

#### ^177^Lu-labeling

Previously published procedures were applied with minor modifications for ^177^Lu-labeling of DOTA-conjugated peptides (Weineisen et al. 2015; Sosabowski and Mather 2006). 5.00 μL of the precursor (0.20 mM in DMSO, 1.00 nmol, 1.00 eq.) was added to 10.0 μL of 1 M NaOAc buffer (aq.) (pH = 5.5). Subsequently, 14 to 65 MBq [^177^Lu]LuCl_3_ (A_s_ >  3000 GBq/mg, 740 MBq/mL, 0.04 M HCl, ITG, Garching, Germany) were added and the mixture was filled up to 100 μL with 0.04 M HCl (in Tracepur®-H_2_O). 10.0 μL of 0.1 M sodium ascorbate (aq.) (in Tracepur®-H_2_O) were added and the reaction mixture was heated for 25 min at 70 to 95 °C. The radiochemical purity (RCP) was determined using radio-RP-HPLC and radio-TLC. Occasionally, purification by cartridge (HLB Plus Light, 30 mg) and radio-RP-HPLC was necessary. For detailed procedures for each peptide, see the ‘METHODS’ section in the supporting information.

#### Lipophilicity

The log D values were determined, using the shake-flask method as previously described (Wurzer et al. 2020). The radiolabeled tracer (0.5–1 MBq, 20 μL) was dissolved in 1 mL of a 1/1 mixture (v/v) of PBS (pH 7.4) and *n*-octanol in a reaction vial (*n* = 6). After vigorous mixing of the suspension for 3 min at r.t., the vial was centrifuged at 9000 rpm (ca. 7700×g) for 5 min at room temperature (Heraeus Biofuge 15, Thermo Fisher Scientific, Darmstadt, Germany) and 150 μL aliquots of both layers were measured in a ɣ-counter.

#### ^125^I-labeling

The radioiodinated reference ligand ([^125^I]I-BA)KuE was synthesized in analogy to a previously described method (Schmidt et al. 2018).

### In vitro experiments

#### Cell culture

PSMA-positive LNCaP cells (300265; Cell Lines Service, Eppelheim, Germany) were cultivated in Dulbecco’s modified Eagle medium/Nutrition Mixture F-12 with GlutaMAX (1/1, DMEM-F12, Thermo Fisher Scientific, Darmstadt, Germany) supplemented with 10% fetal bovine serum (Merck KGaA, Darmstadt, Germany) and kept at 37 °C in a humidified 5% CO_2_ atmosphere. One day (24 ± 2 h) prior to all in vitro experiments, the cultivated LNCaP cells were harvested using a mixture of trypsin/ethylenediamine-tetraacetic acid (0.05%/0.02%) in PBS (Thermo Fisher Scientific, Darmstadt, Germany) and centrifuged at 1300 rpm (ca. 190×g) for 3 min at room temperature (Heraeus Megafuge 16, Thermo Fisher Scientific, Darmstadt, Germany). After centrifugation, the supernatant was disposed and the cell pellet was resuspended in culture medium. Cells were counted with a Neubauer hemocytometer (Paul Marienfeld GmbH & Co. KG, Lauda-Königshofen, Germany) and seeded in 24-well plates. IC_50_ values were determined by transferring 1.50 × 10^5^ cells/mL per well into 24-well plates, whereas internalization was assessed by transferring 1.25 × 10^5^ cells/mL per well into poly-L-lysine (PLL)-coated 24-well plates.

#### Affinity determinations (IC_50_) and internalization studies

Detailed information on affinity and internalization experiments is provided in the supporting information. In brief, competitive binding studies were determined on LNCaP cells (1.50 × 10^5^ cells in 1 mL/well) after incubation at 4 °C for one hour, using ([^125^I]I-BA)KuE (0.20 nM/well) as the reference radioligand (*n* = 3). IC_50_ values of (^nat^I-BA)KuE were determined on the same day for monitoring assay performance and to identify abnormal deviations in uptake and/or affinity. Only values for which the affinity of (^nat^I-BA)KuE was within a range of 3.95 ± 1.35 nM (*n* = 6) were considered. Internalization studies of the radiolabeled ligands (1.0 nM/well) were performed on LNCaP cells (1.25 × 10^5^ cells in 1 mL/well) at 37 °C for one hour and accompanied by ([^125^I]I-BA)KuE (0.20 nM/well), as reference ligand. Data were corrected for non-specific binding and normalized to the specific internalization observed for the radioiodinated reference compound (*n* = 3).

### In vivo experiments

All animal experiments were conducted in accordance with general animal welfare regulations in Germany (German animal protection act, as amended on 18.05.2018, Art. 141 G v. 29.3.2017 I 626, approval no. 55.2–1-54-2532-71-13) and the institutional guidelines for the care and use of animals. To establish tumor xenografts, LNCaP cells (approx. 10^7^ cells) were suspended in 200 μL of a 1/1 mixture (v/v) of DMEM F-12 and Matrigel (BD Biosciences, Heidelberg, Germany) and inoculated subcutaneously onto the right shoulder of 6–8 weeks old CB17-SCID mice (Charles River Laboratories, Sulzfeld, Germany). Mice were used for experiments when tumor size reached 5–10 mm in diameter (3–6 weeks after inoculation).

#### Biodistribution

Approximately 2–10 MBq (0.20 nmol) of the ^177^Lu-labeled PSMA inhibitors were injected into the tail vein of LNCaP tumor xenograft-bearing male CB17-SCID mice (*n* = 3 to 5). They were sacrificed by CO_2_ asphyxiation and cervical dislocation either 1 h or 24 h post injection (p.i.) (*n* = 3 for [^177^Lu]Lu-**5**, -**6** & -**7** (proinhibitor compounds I-III), *n* = 4 for [^177^Lu]Lu-**10** (alkyne), *n* = 5 for [^177^Lu]Lu-**3** (carbamate I) and *n* = 5 for [^177^Lu]Lu-**11** (tetrazole)). Selected organs were removed, weighed and organ activities measured in a γ-counter.

#### Metabolite analysis

Detailed information on organ preparation and extraction procedures is provided in the supporting information. In brief, [^177^Lu]Lu-**11** (9.64 MBq) was injected into the tail vein of a LNCaP tumor xenograft-bearing CB17-SCID mouse. The animal was sacrificed 1 h p.i. and subjected to the standard procedure for biodistribution studies. In addition, urine was taken from all mice that were investigated in this experiment (8.4–9.0 MBq injected activity) and pooled (*n* = 5). Liver, tumor and kidneys were homogenized with a MM-400 ball mill (Retsch GmbH, Haan, Germany) at 30 Hz for 20 min and extracted with 1 mL radioimmunoprecipitation assay (RIPA) buffer containing 2 μmol of 2-(phosphonomethyl)pentane-1,5-dioic acid (2-PMPA). The suspension was centrifuged (15,200 rpm, 10 min, 21 °C) and the supernatants were loaded onto a preconditioned Strata-X cartridge (200 mg). Elution of the activity was conducted by 750 μL of MeCN/H_2_O (6/4, 0.1% TFA) and the extracts were analyzed via radio-RP-HPLC. Prior to solid phase extraction, blood samples were centrifuged twice (13,000 rpm, 5 min) to separate the plasma from the blood cells. Urine samples were also centrifuged (13,000 rpm, 5 min) and the supernatant was directly analyzed via radio-RP-HPLC.

#### *μ*SPECT/CT imaging

Imaging experiments were conducted using a MILabs VECTor^4^ small-animal SPECT/PET/OI/CT. The resulting data were analyzed by the associated PMOD (version 4.0) software. Mice were anaesthetized with isoflurane and the ^177^Lu-labeled PSMA compounds were injected via the tail vein. Mice were euthanized 1 h or 24 h p.i. by CO_2_ asphyxiation and cervical dislocation and blood samples for later biodistribution studies or metabolite analysis were taken by cardiac puncture before image acquisition. Static images were acquired with 45 min acquisition time using the HE-GP-RM collimator and a step-wise multi-planar bed movement. All images were reconstructed using the MILabs-Rec software (version 10.02) and a pixel-based Similarity-Regulated Ordered Subsets Expectation Maximization (SROSEM) algorithm with a window-based scatter correction (20% below and 20% above the photopeak, respectively). Voxel size CT: 80 μm, voxel size SPECT: 0.8 mm, 1.6 mm (FWHM) Gaussian blurring post processing filter, with calibration factor in kBq/mL and decay correction, no attenuation correction.

## Results

### Synthesis

**Scheme 1:**
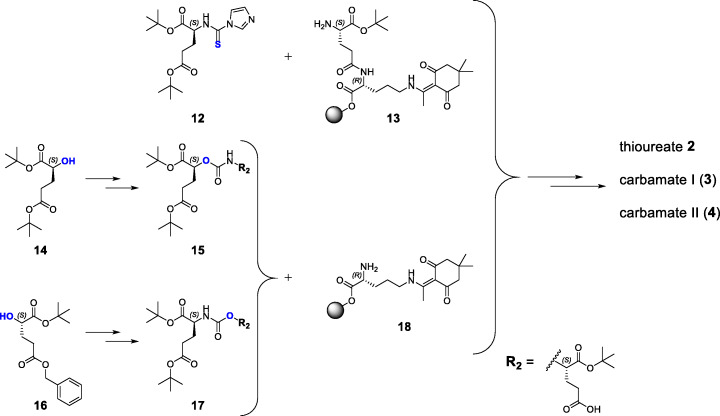
General, simplified synthetic routes for the preparation of thioureate **2**, carbamate I (**3**) and carbamate II (**4**). Synthesis of the binding motif of thioureate **2** was conducted by a solid phase procedure, whereas binding motifs **15** (carbamate I) and **17** (carbamate II) were obtained by solution phase synthesis prior to coupling to compound **18**. Detailed synthesis procedures are given in the supporting information

**Scheme 2:**
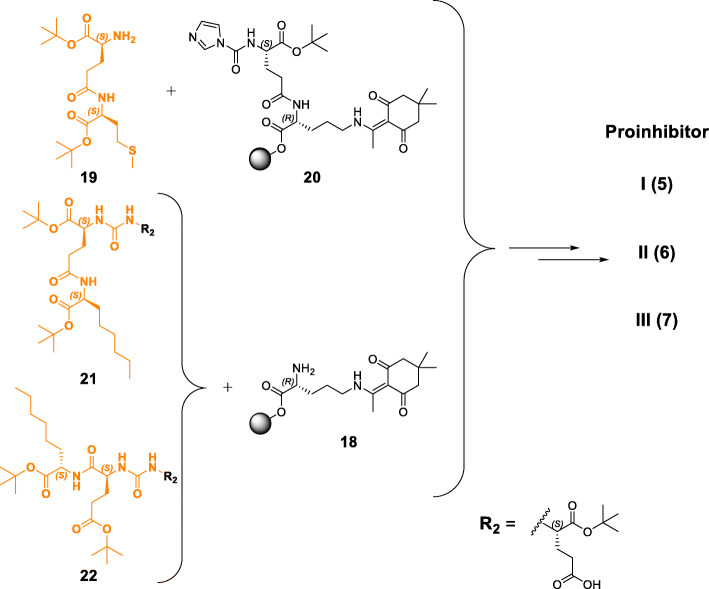
General, simplified synthetic routes for the preparation of proinhibitors I, II & III (**5**, **6** & **7**). Synthesis of the binding motif of proinhibitor I (**5**) was conducted by a solid phase procedure, whereas binding motif **21** was obtained by a mixed solid/solution phase synthesis prior to coupling to compound **18**. Compound **22** (proinhibitor III) could only be obtained by solution phase synthesis. Detailed synthesis procedures are given in the supporting information

**Scheme 3:**
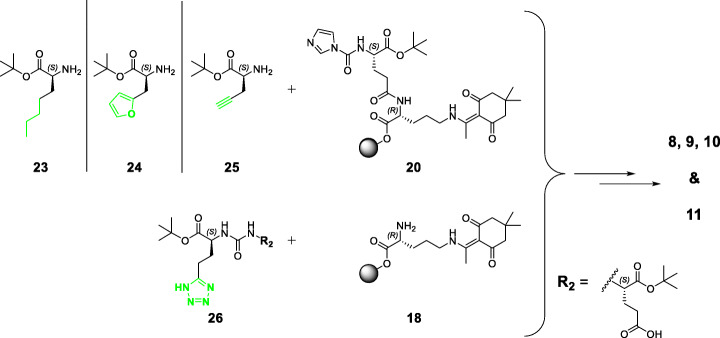
General, simplified synthetic routes for the preparation of L-2-aha (**8**), furyl (**9**), alkyne (**10**) and tetrazole (**11**) derivatives. Syntheses of the binding motifs of compounds **8** (L-2-aha), **9** (furyl) and **10** (alkyne) were conducted by a solid phase procedure, whereas binding motif **26** (tetrazole) was obtained by solution phase synthesis prior to coupling to compound **18**. Detailed synthesis procedures are given in the supporting information

PSMA ligands containing modified binding motifs (Fig. 2) were synthesized according to known or modified organic chemical synthesis procedures. On-resin synthesis of binding motifs was established and adjusted in individual cases (Schemes 1, 2 and 3). Peptide chain elongation was performed according to standard solid-phase peptide synthesis protocols for PSMA derivatives and optimizations concerning (radio)metal complexation reactions were performed if necessary (Tables 1 and 2 in the supporting information). The following sections cover the syntheses of compounds **2** to **11** (Fig. 2), highlighting special synthetical aspects, improvements to already described procedures as well as methods for preservation of the mandatory L-configuration of the PSMA-binding motif during inhibitor modification.

### Synthesis of PSMA derivatives containing modifications within the central Zn^2+^-binding unit: thioureate 2, carbamate I (3) and carbamate II (4)

In order to accomplish substitution of the urea by a thiourea moiety, 1,1’-thiocarbonyldiimidazole was used instead of 1,1’carbonyldiimidazole (CDI) for the synthesis of compound **12**. The binding motif was assembled by incubation of **12** with 2-CT (2-chlorotrityl) resin-bound dipeptide H-L-Glu[D-Orn(Dde)-2-CT]-O*t*Bu (**13**). This solid phase approach was successfully implemented for the Glu-thiourea-Glu moiety and transferred to further synthesis procedures of binding motifs in this study. Purified thioureate **2** was directly complexed with ^nat^Ga^3+^ for in vitro studies.

In contrast to thioureate **2**, syntheses of the PSMA-binding moieties of carbamate I (**15**) and carbamate II (**17**) were performed completely by a solution phase strategy. All synthesis steps towards compound **15** and **17**, starting from enantiopure *(S*)-5-oxotetrahydrofuran-2-carboxylic acid, focused on conditions to ensure and maintain L-configuration of the entire PSMA-binding motif (Zhang et al. 2009; Shin et al. 2000; Weineisen et al. 2014; Yang et al. 2016). By introduction of different protective groups (*tert*-butyl or benzyl) at the γ-carboxylate of compounds **14** and **16**, accommodation of the carbamate oxygen either within the S1’ or S1 pocket of PSMA is already defined. Carbamate I (**3**) was complexed with ^nat^Ga^3+^, ^nat^Lu^3+^ and [^177^Lu]Lu^3+^ for in vitro and in vivo studies, whereas carbamate II (**4**) was complexed with ^nat^Ga^3+^ and evaluated solely in in vitro studies.

### Synthesis of proinhibitors I, II & III (5, 6 & 7)

Dipeptide H-L-Glu(L-Met-O*t*Bu)-O*t*Bu (**19**) was generated in solution and further coupling to resin-bound compound **20** provided the tris-*t*Bu-protected proinhibitor I binding motif, which afforded product **5** after on-resin elongation. Functionalization of resin-bound dipeptide **13** (H-L-Glu[D-Orn(Dde)-2-CT]-O*t*Bu) to yield its carbonylimidazole derivative **20** was required, as functionalization of dipeptide **19** with CDI was not successful. Dimerization as a competing reaction was more favored and hence, only the urea-conjugated dimer of **19** could be isolated after this attempt (unpublished observations). Notably, carbonylimidazole functionalization of resin-bound peptide **13** worked properly in this approach, as a certain distance to the resin anchor (2-chlorotrityl group) was preserved. In contrast, this functionalization did not work at the first 2-CT resin-bound amino acid, provided that it was coupled to the resin by its α-carboxylate (unpublished observations). Proinhibitor I (**5**) was complexed with ^nat^Lu^3+^ and [^177^Lu]Lu^3+^ for in vitro and in vivo studies.

Synthesis of the binding motif of proinhibitor II (**21**) was accomplished via a mixed solid/solution phase strategy, starting with resin-bound Fmoc-L-2-aminooctanoic acid (Fmoc-L-2-Aoc-OH). After multiple steps, compound **21** was obtained, suitable for coupling to resin-bound H-D-Orn(Dde) (**18**). Subsequent on-resin elongation afforded proinhibitor II (**6**), which was complexed with ^nat^Lu^3+^ and [^177^Lu]Lu^3+^ for in vitro and in vivo studies.

In analogy to proinhibitor I (**5**), on-resin synthesis of the binding motif was also pursued for proinhibitor III (**7**). However, in contrast to the procedure for proinhibitor I, the starting dipeptide for proinhibitor III (H-L-Glu(O*t*Bu)-L-2-Aoc-O*t*Bu) did not react and only on-resin urea-conjugated dimerization of peptide **20** was detected (unpublished observations). Therefore, the strategy was changed to solution phase synthesis to obtain tris-*t*Bu-protected binding motif **22**. Coupling to **18** and subsequent on-resin elongation afforded proinhibitor III (**7**), which was complexed with ^nat^Lu^3+^ and [^177^Lu]Lu^3+^ for in vitro and in vivo studies.

### Synthesis of PSMA-binding motifs with substituents & bioisosteres of the P1’-γ-carboxylic acid: L-2-aminoheptanoic acid (2-aha) (8), furyl (9), alkyne (10) and tetrazole (11) derivatives

Reactants **23** and **24** were generated in solution, using the respective amino acid and *tert*-butyl acetate, according to a previously published procedure by Hyun et al. with some minor modifications (Hyun et al. 2010). This strategy was used to assure esterification of the carboxylic acid moiety only, with no unwanted alkylation/carbamation at the free primary amine. *O*-*tert*-butyl-*N,N′*-diisopropylisourea could not be used in this case, as its usage would have led to simultaneous alkylation of the free primary amine (Mathias 1979). Coupling of **23/24** with resin-bound **20** provided an alkane-/furyl-functionalized binding motif, which then afforded product **8/9** after on-resin elongation. Both, L-2-aminoheptanoic acid derivative **8** as well as was furyl derivative **9** were complexed with ^nat^Lu^3+^ for in vitro studies.

Reactant **25** was commercially available and used directly for on-resin coupling with compound **20**. Subsequent peptide chain elongation afforded alkyne derivative **10**, which was complexed with ^nat^Lu^3+^ and [^177^Lu]Lu^3+^ for in vitro and in vivo studies.

Synthesis of the tetrazole bioisostere was successfully accomplished following a multiple step procedure described by Kozikowski et al. (Kozikowski et al. 2004) Binding motif **26** was completed by a solution phase strategy. All synthesis steps, starting from enantiopure *N*-Cbz-L-Glu-O*t*Bu, focused on conditions to ensure and maintain L-configuration of the entire PSMA-binding motif (Kozikowski et al. 2004; Weineisen et al. 2014). After coupling to resin-bound **18** and further peptide chain elongation, tetrazole derivative **11** was obtained and complexed with ^nat^Lu^3+^ and [^177^Lu]Lu^3+^ for in vitro and in vivo studies.

### Cold metal complexation and radiolabeling

Cold metal complexation with molar excess of Ga(NO_3_)_3_*6 H_2_O (3.50-fold molar excess) or LuCl_3_ (6-fold molar excess) led to formation of the respective ^nat^Ga/^nat^Lu-PSMA ligands. Purification of the crude ^nat^Ga-ligand was performed by RP-HPLC. ^nat^Lu-complexation mixtures (0.5–1.0 mM in DMSO/H_2_O = 1/1) were directly used as stock solutions for affinity determination, as chemical purity was always > 92%. Reaction conditions, chemical purities and yields of the investigated ^nat^Ga- and ^nat^Lu-PSMA ligands are given in the supporting information (Table 1).

Radiolabeling ([^177^Lu]Lu^3+^) was performed using manual procedures and radiochemical purity (RCP), as determined by radio-RP-HPLC and radio-thin-layer chromatography was 96.4 ± 2.2% (*n* = 16) with exception of proinhibitor I (**5**) (89.3 ± 1.9% RCP, *n* = 4). In case of incomplete complexation, removal of free [^177^Lu]Lu^3+^ via HLB cartridge (30 mg) was performed. Moreover, in case of proinhibitor I, radioactive by-products were tried to remove by preparative radio-RP-HPLC. Reaction conditions, isolated radiochemical yields (RCY), apparent molar activities (A_m_) at the end of synthesis and purification, the range of used activities as well as radiochemical purities (RCP) are given in the supporting information (Table 2). Detailed synthesis procedures for cold metal complexation and radiolabeling are described in the ‘METHODS’ section in the supporting information.

### In vitro characterization

#### PSMA affinity

Binding to PSMA was determined using LNCaP human prostate cancer cells in a competitive binding assay with ^nat^Ga or ^nat^Lu complexes of compounds **2** to **11**. For comparison, IC_50_ values of ^nat^Lu-PSMA-10 (^nat^Lu-**1**) (2.8 ± 0.5 nM) were also determined. The results show loss of affinity to a varying extent for all modifications differing from glutamate at P1’ position (Table 1). Only ^nat^Lu-**3** (carbamate I) and ^nat^Lu-**11** (tetrazole derivative) still exhibited high affinity (7.1 ± 0.7 nM and 16.4 ± 3.8 nM, respectively) towards PSMA-expressing LNCaP cells.

**Table 1:** PSMA-binding affinities (IC_50_), internalization (%) and lipophilicity (log D) of the investigated compounds^a^

PSMA inhibitor	IC_50_	%Internalization compared to the reference^b^	log D
^nat^Lu/[^177^Lu]Lu-**1**	2.8 ± 0.5 nM	1 h: 177 ± 15	−3.78 ± 0.01
^nat^Ga-**2**	> 3 μM (*n* = 2)	n.d.	n.d.
^nat^Ga-**3**	21.3 ± 1.7 nM	n.d.	n.d.
^nat^Lu/[^177^Lu]Lu-**3**	7.1 ± 0.7 nM	1 h: 67.8 ± 0.5	− 3.40 ± 0.45
^nat^Ga-**4**	> 1 μM (*n* = 2)	n.d.	n.d.
^nat^Lu/[^177^Lu]Lu-**5**	26 ± 16 μM	0.5 h: 0.00 ± 0.00 1 h: 0.00 ± 0.00 2 h: 0.17 ± 0.32 4 h: 0.01 ± 0.05	−2.89 ± 0.18
^nat^Lu/[^177^Lu]Lu-**6**	2.5 ± 1.2 μM	0.5 h: 0.00 ± 0.00 1 h: 0.00 ± 0.10 2 h: 0.03 ± 0.03 4 h: 0.01 ± 0.06	−2.52 ± 0.22
^nat^Lu/[^177^Lu]Lu-**7**	6.3 ± 3.6 μM	0.5 h: 0.00 ± 0.00 1 h: 0.00 ± 0.00 2 h: 0.00 ± 0.00 4 h: 0.00 ± 0.00	−2.37 ± 0.22
^nat^Lu-**8**	> 2 μM (*n* = 2)	n.d.	n.d.
^nat^Lu-**9**	> 440 nM (*n* = 2)	n.d.	n.d.
^nat^Lu/[^177^Lu]Lu-**10**	138 ± 53 nM	1 h: 1.2 ± 0.4	− 2.83 ± 0.08
^nat^Lu/[^177^Lu]Lu-**11**	16.4 ± 3.8 nM	1 h: 9.9 ± 3.2	− 2.91 ± 0.05

^a^Binding assays (IC_50_) were performed using LNCaP cells (150,000 cells/well) and ([^125^I]I-BA)KuE (c = 0.2 nM) as radioligand. Cells were incubated in HBSS (1% BSA) at 4 °C for 1 h. ^b^Internalization values were corrected for unspecific binding and normalized to the external reference ([^125^I]I-BA)KuE (13.0 ± 2.5% internalization at 1 h (*n* = 21), c = 0.2 nM; 1.0 nM for ^177^Lu-compounds; 37 °C, 1 h, 125,000 cells/well, PLL-coated plates). Data for binding (IC_50_) and internalization are expressed as mean ± SD (*n* = 3) unless otherwise stated. Data are expressed as mean ± SD (*n* = 6) for log D

#### Internalization

LNCaP cells were used to investigate internalization of the ^177^Lu-labeled compounds **1**, **3**, **5**, **6**, **7**, **10** and **11**. No internalization studies were performed on thioureate **2**, carbamate II (**4**), L-2-aminoheptanoic acid derivative **8** and furyl derivative **9**, as these candidates were excluded due to poor IC_50_ data. Normalized to the uptake of ([^125^I]I-BA)KuE, the results in Table 1 show that all modified compounds exhibited significantly lower values in comparison to [^177^Lu]Lu-PSMA-10 ([^177^Lu]Lu-**1**) (177 ± 15%). ^nat^Lu-**3** (carbamate I) displayed an internalization of 67.8 ± 0.5% compared to ([^125^I]I-BA)KuE and hence, showed highest internalization of all modified inhibitor derivatives. For [^177^Lu]Lu-**10** (alkyne) and [^177^Lu]Lu-**11** (tetrazole), both lacking a glutamate moiety at the P1’ position, only weak internalization was detected (1.2 ± 0.4% and 9.9 ± 3.2% compared to the reference, respectively). For all proinhibitor motifs internalization studies were conducted prior to any affinity determination, in order to investigate possible substrate cleavage kinetics. Internalization at 37 °C was determined at several time points of 0.5 h, 1 h, 2 h and 4 h. All in all, no internalization at any time point could be detected.

#### Lipophilicity

In analogy to internalization studies, log D values were determined for ^177^Lu-labeled compounds **1**, **3**, **5**, **6**, **7, 10** and **11** (Table 1). Within the proinhibitor subgroup, methionine-functionalized derivative **5** displayed the most hydrophilic character with a log D value of − 2.89 ± 0.18. Moreover, [^177^Lu]Lu-**3** (carbamate I) represents the most hydrophilic compound of all modified PSMA inhibitors (log D = − 3.40 ± 0.45), comparable to [^177^Lu]Lu-**1** (log D = − 3.78 ± 0.01).

### In vivo characterization

#### Biodistribution and *μ*SPECT/CT imaging

**Fig. 3:**
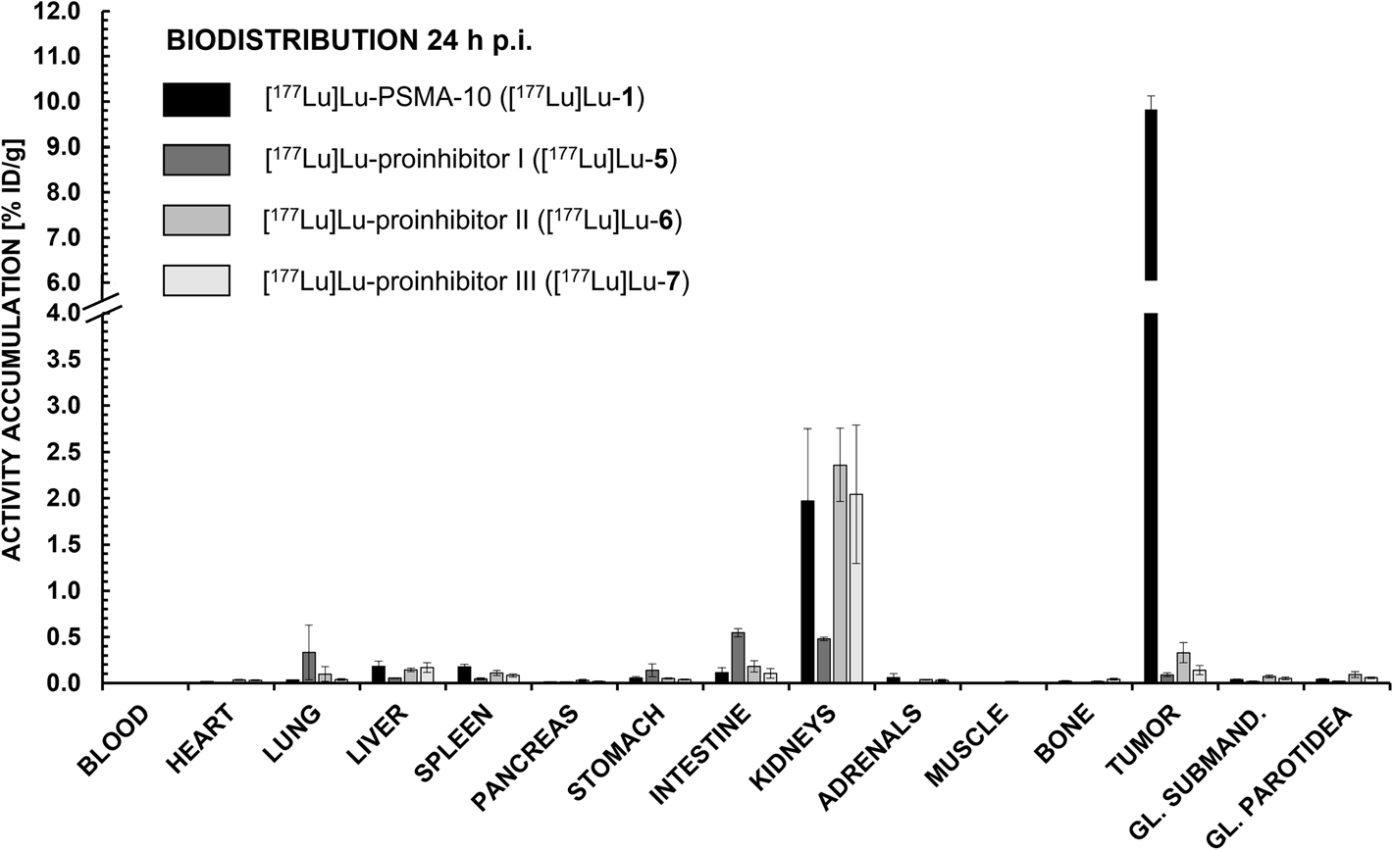
Biodistribution data (% ID/g) of [^177^Lu]Lu-PSMA-10 ([^177^Lu]Lu-**1**), [^177^Lu]Lu-**5**, [^177^Lu]Lu-**6** and [^177^Lu]Lu-**7** in tumor xenograft-bearing CB17-SCID mice at 24 h p.i. (*n* = 2 for [^177^Lu]Lu-5*, *n* = 3 for [^177^Lu]Lu-**6** and [^177^Lu]Lu-**7** and *n* = 5 for [^177^Lu]Lu-PSMA-10 ([^177^Lu]Lu-**1**)). Submandibular and parotid glands were dissected separately and their values are depicted in the columns ‚GL. SUBMAND.‘and ‚GL. PAROTIDEA‘, respectively. *Ingestion of radioactively contaminated animal feed led to putative high activities in stomach and intestine, therefore excluding all values of mouse 3

[^177^Lu]Lu-**5**, -**6** and- **7** (proinhibitors I-III) were evaluated (*n* = 3) in tumor-bearing CB-17 SCID mice prior to any affinity measurement. However, in vivo data in general reflected the low internalization values for all proinhibitors, with a maximum tumor accumulation of 0.33 ± 0.11% ID/g for [^177^Lu]Lu-**6** (proinhibitor II) and a minimum tumor accumulation of 0.09 ± 0.02% ID/g for [^177^Lu]Lu-**5** (proinhibitor I) as depicted in Fig. 3. Furthermore, non-target tissue uptake was on the scale of [^177^Lu]Lu-PSMA-10 ([^177^Lu]Lu-**1**) or even higher.

**Fig. 4:**
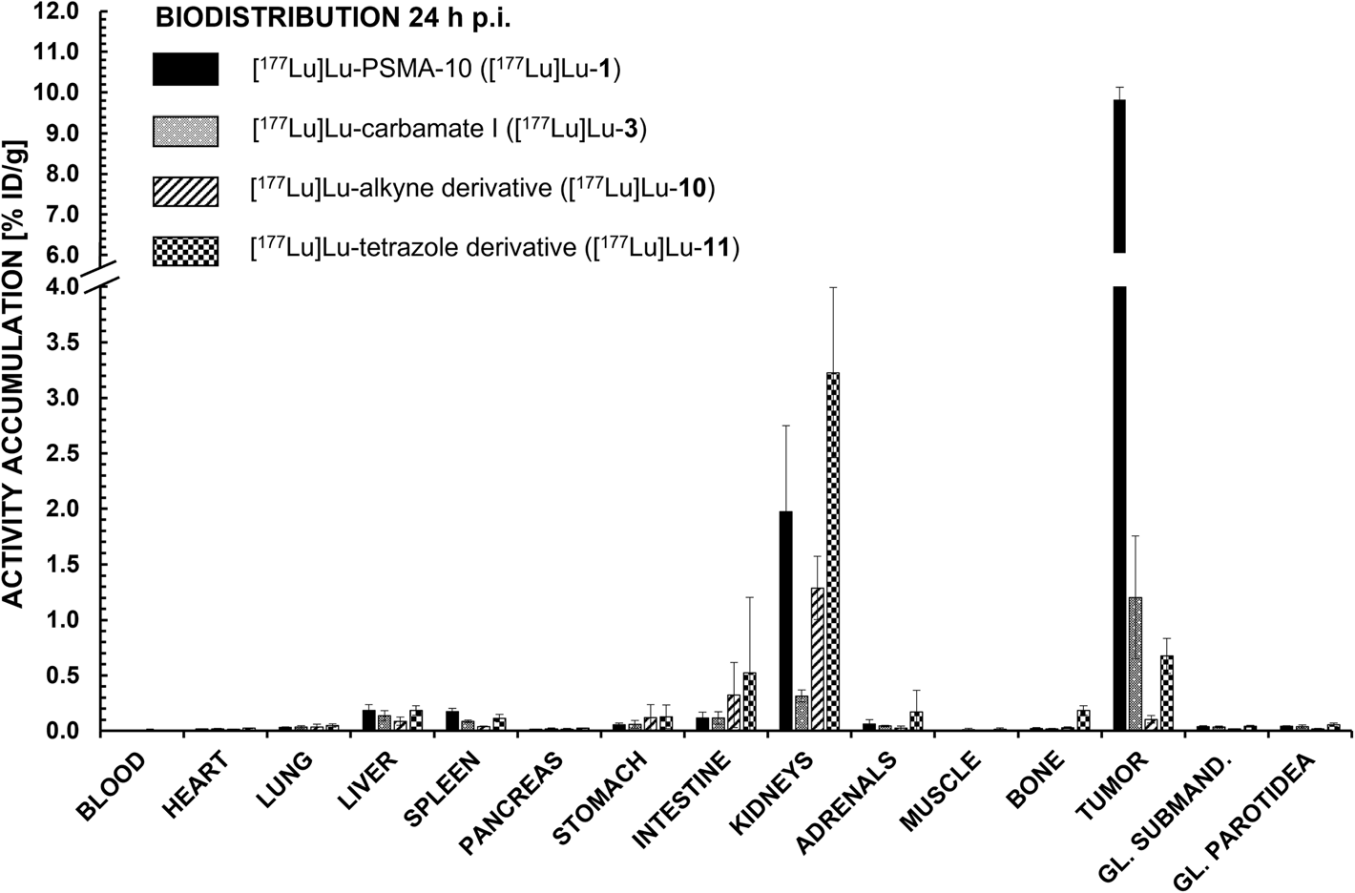
Biodistribution data (% ID/g) of [^177^Lu]Lu-PSMA-10 ([^177^Lu]Lu-**1**), [^177^Lu]Lu-**3**, [^177^Lu]Lu-**10** and [^177^Lu]Lu-**11** in tumor xenograft-bearing CB17-SCID mice at 24 h p.i. (*n* = 4 for [^177^Lu]Lu-**10** and *n* = 5 for all other experiments). Submandibular and parotid glands were dissected separately and their values are depicted in the columns ‚GL. SUBMAND.‘and ‚GL. PAROTIDEA‘, respectively

**Fig. 5:**
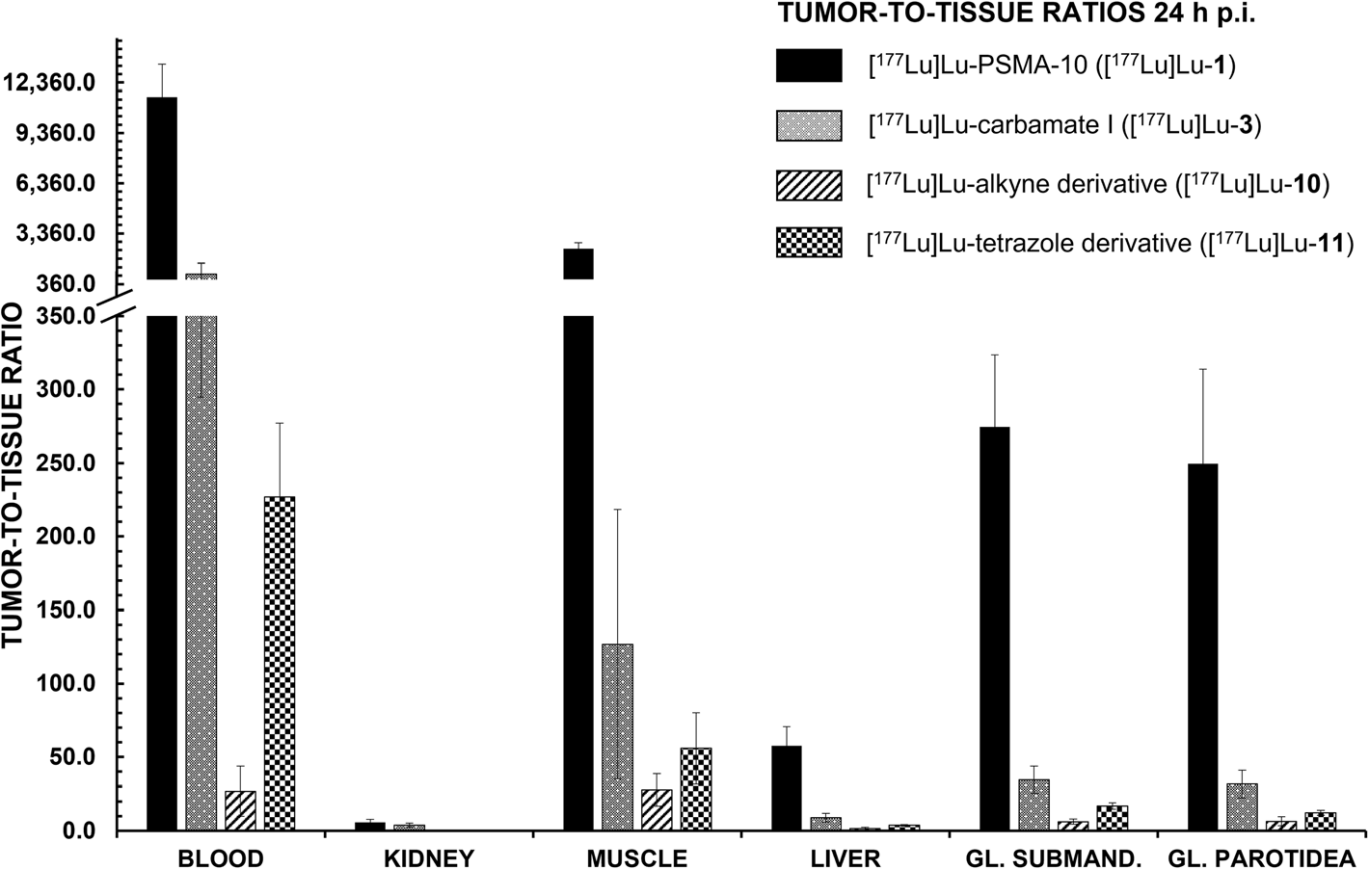
Tumor-to-tissue ratios of [^177^Lu]Lu-PSMA-10 ([^177^Lu]Lu-**1**), [^177^Lu]Lu-**3**, [^177^Lu]Lu-**10** and [^177^Lu]Lu-**11** in selected organs at 24 h p.i. (*n* = 4 for [^177^Lu]Lu-**10** and *n* = 5 for all other experiments)

For further in vivo studies, derivatives with medium to high affinity were chosen (i.e. [^177^Lu]Lu-**3**, -**10** and -**11**) (Fig. 4). For all modified peptides, a distinctly lower tumor accumulation (0.10 to 1.20% ID/g) at 24 h p.i. in comparison to the original [^177^Lu]Lu-PSMA-10 compound (9.82 ± 0.30% ID/g) could be observed with unchanging salivary gland uptake. Apparently, tumor-to-submandibular gland and tumor-to-parotid gland ratios (Fig. 5) declined by a factor of 8 for [^177^Lu]Lu-**11** (tetrazole) up to a factor of 45 for [^177^Lu]Lu-**10** (alkyne). However, salivary gland uptake in general stayed at an equally low level for all EuE- and non-EuE-based inhibitor motifs (between 0.02 ± 0.00% ID/g and 0.09 ± 0.03% ID/g). Residual tumor-to-tissue ratios observed for [^177^Lu]Lu-**3**, -**10** and -**11** were markedly lower than for [^177^Lu]Lu-PSMA-10 (Fig. 5). Tumor-to-tissue ratios of proinhibitors were not determined, as uptake in tumor xenografts was very low (≤ 0.33 ± 0.11% ID/g) (Fig. 3), with no significant change in kidney accumulation or other non-target tissues.

Tumor uptake for [^177^Lu]Lu-**3** (carbamate I) and -**11** (tetrazole) was also analyzed 1 h p.i. and compared to [^177^Lu]Lu-**1** (Table 4 in the supporting information). At this early time point, tumor accumulation of [^177^Lu]Lu-**3** was already only about half of the uptake obtained for [^177^Lu]Lu-**1** (5.31 ± 0.94% ID/g for [^177^Lu]Lu-**3** vs. 12.2 ± 1.8% ID/g for [^177^Lu]Lu-**1**). [^177^Lu]Lu-**11** showed even lower tumor uptake (3.40 ± 0.63% ID/g). Kidney uptake was distinctly lower for both substances (61.8 ± 25.9% ID/g for [^177^Lu]Lu-**3** and 33.2 ± 3.8% ID/g for [^177^Lu]Lu-**11** vs. 173 ± 56% ID/g for [^177^Lu]Lu-**1**) but activity accumulation in all other non-target tissues stayed in the same range. Interestingly, salivary gland uptake of both peptides was found to be distinctly higher at 1 h p.i. (between 0.37 ± 0.08% ID/g and 0.62 ± 0.20% ID/g) than at 24 h p.i. (Tables 3 and 4 in the supporting information). This resulted in two-fold lower tumor-to-submandibularis and -parotidea ratios of [^177^Lu]Lu-**3** and -**11** at 1 h p.i. compared to their respective values at 24 h p.i. (Tables 5 and 6 in the supporting information).

**Fig. 6:**
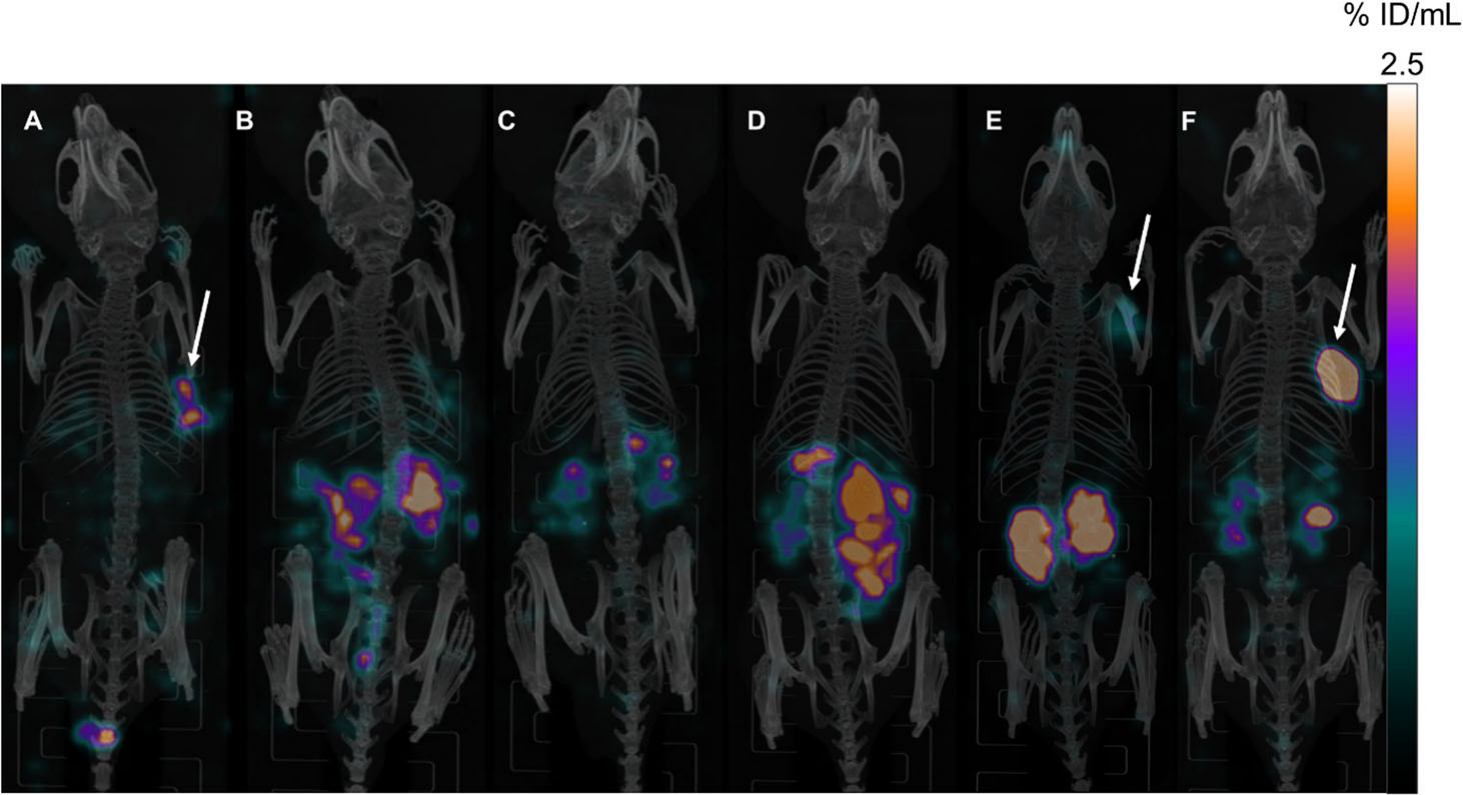
Maximum intensity projections (MIPs) of *μ*SPECT/CT scans in LNCaP xenograft-bearing mice, acquired 24 h p.i. of (**a**) [^177^Lu]Lu-**3** (carbamate I) (3.2 MBq) (**b**) [^177^Lu]Lu-**6** (proinhibitor II) (5.3 MBq) (**c**) [^177^Lu]Lu-**7** (proinhibitor III) (4.5 MBq) (**d**) [^177^Lu]Lu-**10** (alkyne) (9.7 MBq) (**e**) [^177^Lu]Lu-**11** (tetrazole) (7.0 MBq) (**f**) [^177^Lu]Lu-PSMA-10 (**1**) (2.8 MBq). For clear comparison, all images were scaled to the same maximum uptake value (2.5% ID/mL). Arrows indicate apparent tumor uptake in (**a**), (**e**) and (**f**). No *μ*SPECT/CT image is displayed for [^177^Lu]Lu-**5** (proinhibitor I), as ingestion of radioactive animal feed led to putative high activities in stomach and intestine. Therefore, all values of this mouse (= mouse 3) from the respective biodistribution study and also its *μ*SPECT/CT image were excluded. Static images were acquired *post mortem* (CO_2_ asphyxiation and cervical dislocation) and after cardiac puncture with an acquisition time of 45 min. Further biodistribution studies were performed after the scan and included in the calculation of % ID/g values provided by Figs. 3 and 4 within this manuscript and Table 3 in the supporting information

For visualization of the biodistribution data, maximum intensity projections (MIPs) of *μ*SPECT/CT scans in LNCaP xenograft-bearing mice, acquired 24 h p.i. of [^177^Lu]Lu-**3**, [^177^Lu]Lu-**6**, [^177^Lu]Lu-**7**, [^177^Lu]Lu-**10**, [^177^Lu]Lu-**11** and [^177^Lu]Lu-**1** ([^177^Lu]Lu-PSMA-10) are depicted in Fig. 6. Arrows indicate apparent tumor uptake of [^177^Lu]Lu-**3**, [^177^Lu]Lu-**11** and [^177^Lu]Lu-**1**.

### Metabolite analysis

Besides biodistribution and *μ*SPECT/CT studies, [^177^Lu]Lu-**11** (tetrazole) was analyzed according to its metabolic stability 1 h p.i. Relevant dissected organs (tumor, kidney, liver) and body fluids (blood and urine) were collected, homogenized if necessary and subjected to extraction of the incorporated activity. Tissue extracts were analyzed by radio-RP-HPLC at a predefined gradient (25–40% MeCN (0.1% TFA) in 20 min), for which the retention time (18.3 min) of the intact cold standard (^nat^Lu-**11**) was previously determined and hence, served as a reference. Only one metabolite with higher hydrophilicity was detected in tumor (7.1%), blood (8.5%) and kidney (11.7%), with the maximum percentage of metabolite amount found in the urine (18.7%). This metabolite was not detectable in the liver homogenate, instead a more lipophilic metabolite was observed at t_*R*_ = 20.5 min (28.0%). The respective radio-RP-HPLC analyses of extracts from homogenized organs and body fluids are depicted in Figure 1 in the supporting information.

## Discussion

With the aim to develop radiolabeled small molecule-based PSMA inhibitors with reduced non-target tissue uptake in patients, especially in salivary and lacrimal glands, several modified PSMA-10 ligands were prepared and further evaluated in vitro and in vivo. Comparison of salivary gland uptake values at 24 h p.i. should give a first indication whether a clear distinction of modified PSMA (pro)inhibitors from classical L-Glu-urea-X binding motifs could be provided by this animal model, established in our group for preclinical evaluation of PCa radiotherapeutics.

### Synthesis

For all peptides, the starting material as well as further synthetical procedures were adjusted in order to preserve L-configuration of the inhibitor motif, since high affinity binding to PSMA basically depends on steric orientation (Barinka et al. 2019; Ferraris et al. 2012). The introduction of different protective groups (*tert*-butyl or benzyl) at the γ-carboxylate of the carbamate-oxygen-bearing moiety was of utmost importance for both carbamates I & II (**3** & **4**). These steps, already conducted at an early stage of synthesis, were crucial for determination of carbamate orientation.

On-resin synthesis of the binding motif was first established for thioureate **2** and simplified the reaction process significantly. In contrast to L-Glu-urea-L-Lys binding motifs (Mosayebnia et al. 2018), PSMA inhibitor motifs based on L-Glu-urea-L-Glu were synthesized exclusively in solution in previously published attempts (Wurzer et al. 2020; Robu et al. 2018; Lu et al. 2013; Kularatne et al. 2009). Related time- and substance-consuming steps (purification, removal of an orthogonal protective group at P1 position, etc.) could be avoided by this on-resin method. Hence, this strategy was also used for the synthesis of subsequent derivatives (proinhibitor I (**5**), L-2-aha derivative **8**, furyl derivative **9** and alkyne derivative **10**). Moreover, it might serve as a general procedure for the preparation of L-Glu-urea-L-Glu-based PSMA inhibitors.

Syntheses of the binding motifs of proinhibitors **5**–**7** had to be adjusted due to unreactive starting material or unwanted dimerization reactions. In addition, recurring methionine oxidation resulted in low overall yields (0.08%) for proinhibitor I (**5**) after RP-HPLC purification.

### Cold metal complexation and radiolabeling

In general, all derivatives containing modifications within the Zn^2+^-binding unit showed enhanced formation of intramolecular condensation by-products during complexation (unpublished observation). Therefore, all ^nat/177^Lu-complexation reactions of **3** were performed at 70 °C, as formation of unwanted by-products could be almost completely reduced. Oxidation tendency for proinhibitor I (**5**) remained at ^177^Lu-labeling and also during radio-RP-HPLC purification, which impeded the preparation of products with RCP higher than 90%, even at lower temperatures (70–80 °C).

### In vitro *&* in vivo characterization

Though IC_50_ and lipophilicity data of carbamate I (**3**) were comparable to [^177^Lu]Lu-PSMA-10 ([^177^Lu]Lu-**1**), internalization was distinctly lower (67.8 ± 0.5% for [^177^Lu]Lu-**3** vs. 177 ± 15% for [^177^Lu]Lu-**1**), which might explain decreased tumor accumulation at 1 h and 24 h p.i. However, low internalization may not be the only reason for decreased tumor uptake. As observed for SST_2_ antagonists, high tumor uptake can also be reached with a negligible capacity to internalize (Dude et al. 2017). A two-fold lower tumor accumulation compared to [^177^Lu]Lu-**1** already 1 h p.i. (5.31 ± 0.94% ID/g) in combination with a rapid decline to 1.20 ± 0.55% ID/g at 24 h p.i., led to the assumption that in vivo decomposition of the inhibitor motif might have generated a non-PSMA-binding ligand, resulting in fast renal excretion (0.31 ± 0.05% ID/g for [^177^Lu]Lu-**3** vs. 1.97 ± 0.78% ID/g for [^177^Lu]Lu-**1**, 24 h p.i.). Applications of carbamate-based prodrugs, liberating the biologically active substance by in vivo hydrolysis, support this theory (Ghosh and Brindisi 2015).

Similar in vitro results as obtained for carbamate I and II were reported by Yang et al. and Barinka et al. (Yang et al. 2016; Barinka et al. 2019) These observations emphasize the necessity of a hydrogen bond donor at the (non-pharmacophore) P1 position and provide a certain flexibility within the pharmacophore S1’ subpocket.

Since thioureate derivative **2** revealed sulfur to be less tolerated inside the binding pocket, it was assumed that thiourethane derivatives (= combination of carbamate I or II with thioureate) would also lead to poor results. In consequence, their synthesis was not further pursued.

For all proinhibitors, internalization studies were conducted first in order to investigate possible substrate cleavage kinetics. Since no internalization could be detected at any time point (0.5 h, 1 h, 2 h and 4 h) for [^177^Lu]Lu-**5**, - **6** or - **7** (Table 1), it was assumed that no cleavage occurred under these conditions. As we suggested that cleavage of the proinhibitor motifs might be strongly dependent on the tumor cells’ microenvironment, in vivo studies were directly conducted after internalization experiments. With a maximum tumor accumulation of 0.33 ± 0.11% ID/g for [^177^Lu]Lu-**6** (proinhibitor II) and a minimum tumor accumulation of 0.09 ± 0.02% ID/g for [^177^Lu]Lu-**5** (proinhibitor I), all investigated proinhibitors showed very low ability to bind to PSMA-expressing tumors (24 h p.i.), as depicted in Fig. 3. Furthermore, non-target tissue uptake was on the scale of [^177^Lu]Lu-PSMA-10 ([^177^Lu]Lu-**1**), wherefore no tumor-to-tissue ratios were calculated. It was assumed that proinhibitor cleavage probably did not occur in in vitro and in vivo experiments, due to the low (micromolar) affinities of these conjugates determined by additional competitive binding experiments (Table 1). For this reason, synthesis and evaluation of proinhibitor IV (= methionine at the α-carboxylate) was abandoned, as no positive results were expected.

As presumed for the tetrazole moiety (Herr 2002), in vitro studies confirmed a slightly increased lipophilicity for [^177^Lu]Lu-**11** (~ 7.4-fold increase compared to [^177^Lu]Lu-**1**). A noticeable decreased internalization of [^177^Lu]Lu-**11** (9.9 ± 3.2%) with concomitant high affinity (16.4 ± 3.8 nM) did not lead to favorable in vivo results. As the metabolite proportion was rather low in tumor tissue (7.1%) and circulating blood (8.5%), low retention of [^177^Lu]Lu-**11** within the LNCaP tumor xenograft at 1 h p.i. cannot be attributed to severe metabolic instability. Therefore, poor in vivo performance at 1 h p.i. (3.40 ± 0.63% ID/g) as well as at 24 h p.i. (0.68 ± 0.16%ID/g,) was mainly assigned to the overall lower internalization in combination with the decreased hydrophilicity and affinity and of the final ligand.

Low accumulation of alkyne derivative [^177^Lu]Lu-**10** in tumor tissue (0.10 ± 0.03% ID/g, 24 h p.i.) as well as weak internalization (1.2 ± 0.4% compared to the reference) could be attributed to the medium affinity of ^nat^Lu-**10** (138 ± 53 nM).

Apparently, tumor-to-submandibular and tumor-to-parotid gland values of [^177^Lu]Lu-**3** both decreased by a factor of 8 when compared to [^177^Lu]Lu-**1**. An even higher decrease (16 to 20 times lower) was observed for [^177^Lu]Lu-**11 (**tetrazole) and the alkyne analog [^177^Lu]Lu-**10**, which exhibited the lowest tumor-to-salivary gland ratios (39–45 times lower than for [^177^Lu]Lu-**1**) (Fig. 5). However, reduced tumor-to salivary gland values at 24 h p.i. in comparison to reference compound [^177^Lu]Lu-**1** were found to be mainly induced by the decreased tumor accumulation rather than by an altered salivary gland uptake (Fig. 4 in this manuscript and Table 3 in the supporting information). Salivary gland uptake values of EuE- and non-EuE-based ligands ranged between 0.02 ± 0.00% ID/g and 0.09 ± 0.03% ID/g and hence, revealed no significant difference. At least for EuE-based reference [^177^Lu]Lu-**1** a higher activity accumulation was expected, which in turn accounts for a certain unsuitability of this animal model concerning the development of ligands for RLT with reduced salivary gland uptake (discussed in more detail in ‘*Critical assessment of the animal model’*).

High IC_50_ values led to cessation of further in vitro and in vivo investigations for thioureate **2**, carbamate II (**4**), L-2-aha derivative **8** and furyl derivative **9**. Theoretically, a multitude of other substituents as P1’-γ-carboxylic acid bioisosteres might be conceivable (Lassalas et al. 2016). IC_50_ data of the respective PSMA ligands can give a first indication whether further in vitro and in vivo investigations are rational. However, a successful transfer of promising in vitro results to the in vivo situation cannot be guaranteed, as demonstrated by carbamate I (**3**) and tetrazole derivative **11**.

### Critical assessment of the animal model

Activity uptake in the salivary glands of mice was found to be constantly at a very low level (between 0.02 ± 0.00% ID/g and 0.09 ± 0.03% ID/g) irrespective of the use of EuE- (**1**) or non-EuE-based (**3**, **5**, **6**, **7**, **10**, **11**) PSMA ligands (Fig. 4 in this manuscript and Table 3 in the supporting information). The low overall tracer uptake in salivary glands at 24 h p.i. might indicate for a rather unspecific accumulation and that species-dependent differences possibly not allow for a similar radioligand uptake as observed in humans (Knedlik et al. 2017; Roy et al. 2020). Possibly only early time points (e.g. 1 h p.i.) enable a clear distinction. For future studies, the currently used animal model should be re-evaluated at different time points by examination of the salivary gland uptake of [^68^Ga]Ga-PSMA-11 or [^177^Lu]Lu-**1** and possible blockable tracer accumulation when monosodium glutamate is pre-administered (Rousseau et al. (Rousseau et al. 2018)). An alternative animal model should be envisaged if radioligand uptake emerges to be non-blockable or if again low overall tracer uptake does not allow for a proper discrimination. Re-evaluation of high affinity compounds [^177^Lu]Lu-**3** and [^177^Lu]Lu-**11** in an optimized animal model remains questionable or even inappropriate, due to their low accumulation at 24 h p.i. in tumor xenografts (1.20 ± 0.55% ID/g for [^177^Lu]Lu-**3** and 0.68 ± 0.16%ID/g for [^177^Lu]Lu-**11**) which clearly emphasizes their unsuitability for RLT in PCa patients.

### Critical assessment of the rationale for inhibitor modification

PSMA ligands containing modifications within the inhibitor motif (like in compounds **2** to **11**) might be overestimated if considered as the sole strategy for reduction of non-target tissue uptake in PCa patients. Although small molecule/anion/glutamate transporter systems were proposed for non-target tissue accumulation (Rousseau et al. 2018), the exact mechanism of how monosodium glutamate affects non-target binding of radiolabeled PSMA inhibitors remains still unknown. This effect could also originate from differences in physiological pH of healthy salivary gland parenchyma and poorly differentiated tumor tissue and/or tumor-associated stroma (Warburg effect) (Vāvere et al. 2009). Previous Western blot analyses have shown a variation of the PSMA protein within the salivary glands by 20 kDa (in total: 120 kDa; PSMA of LNCaP cell extracts: 100 kDa) (Troyer et al. 1995). Additionally, genetic analyses revealed mRNA expression of PSMA in salivary glands (Israeli et al. 1994; O'Keefe et al. 2004). Posttranslational modified versions of PSMA (e.g. different glycosylation pattern, otherwise than on PCa) on the surface of salivary gland cells might serve as a reasonable concept for integrating all these contradictory findings (Troyer et al. 1995; Barinka et al. 2012). Binding of antibodies that are highly specific towards epitopes of PCa-related PSMA (intra- or extracellular) would not be possible in this case (Rupp et al. 2019; Horoszewicz et al. 1987; Lopes et al. 1990; Bander et al. 2005). By contrast, small molecule ligands would still be able to bind, as their binding mechanism is probably not affected by posttranslational changes at the surface of the protein. Modifications within the ligand structure would hence result in no benefit.

Furthermore, inconsistent in vitro results concerning the binding of PSMA-specific mAbs to salivary gland tissue are still present (Rupp et al. 2019; Wolf et al. 2010). This indicates that not enough fundamental research was performed yet to provide a clear statement of PSMA expression on salivary gland parenchyma. As long as there is no reliable information concerning this issue, further investigations on ligand modifications might be dispensable.

## Conclusions

No modified inhibitor structure was found to be able to compete with favorable in vivo characteristics of EuE-based PSMA inhibitor [^177^Lu]Lu-PSMA-10. In consequence, the development of further strategies to minimize radioligand uptake in salivary glands should be envisaged.

## Supplementary Information

**Additional file 1.** Supporting Information is provided in addition to data presented in the main manuscript portion, including detailed information on all methods for synthesis and analysis as well as on the used instruments. Furthermore, detailed procedures for ligand synthesis, cold metal complexation and radiolabeling are described. Methods for in vitro and in vivo characterizations are given in more detail, as well as radio-RP-HPLC analyses and extraction efficiencies of the metabolite analysis.

## Data Availability

The datasets supporting the conclusions of this article are included within this article and its additional file.

## Abbreviations

2-Aha: 2-Aminoheptanoic acid
2-Aoc: 2-Aminooctanoic acid
2-CT: 2-Chlorotrityl
2-PMPA: 2-(Phosphonomethyl)pentane-1,5-dioic acid
A_m_: Molar activity
ARG: Autoradiography
BSA: Bovine serum albumin
Bzl: Benzyl
Cbz: Carbobenzoxy
CDI: 1,1’-Carbonyldiimidazole
CT: Computed tomography
Dde: *N*-1-(4,4-Dimethyl-2,6-dioxocyclohex-1-ylidene)ethylamine
DMEM: Dulbecco’s Modified Eagle’s Medium
DMSO: Dimethyl sulfoxide
DOTA: 1,4,7,10-Tetraazacyclododecane-1,4,7,10-tetraacetic acid
EuE: L-Glu-urea-L-Glu
KuE: L-Lys-urea-L-Glu
FOLH1: Folate hydrolase 1
FWHM: Full width at half maximum
GCP II: Glutamate carboxypeptidase II
HBSS: Hank’s buffered salt solution
IC_50_: Half maximal inhibitory concentration
ID/g: Injected dose per gram
ID/mL: Injected dose per milliliter
IHC: Immunohistochemistry
k_cat_: Turnover number
K_m_: Michaelis-Menten constant
mAb: Monoclonal antibody
mCRPC: Metastatic castration-resistant prostate cancer
MeCN: Acetonitrile
MeOH: Methanol
MIP: Maximum intensity projection
mRNA: Messenger ribonucleic acid
NAAG: *N*-Acetyl-L-aspartyl-L-glutamate
NAALADase: *N*-acetylated alpha-linked acidic dipeptidase
OI: Optical imaging
PBS: Phosphate-buffered saline
PCa: Prostate cancer
PET: Positron emission tomography
PSMA: Prostate-specific membrane antigen
RCP: Radiochemical purity
RCY: Radiochemical yield
RIPA: Radioimmunoprecipitation assay
RLT: Radioligand therapy
RP-HPLC: Reversed-phase high-performance liquid chromatography
r.t.: Room temperature
SPECT: Single-photon emission computed tomography
SST_2_: Somatostatin receptor subtype 2
SUV: Standardized uptake value
*t*Bu: *Tert*-butyl
TFA: Trifluoroacetic acid

## References

[CR1] Afshar-Oromieh A, Avtzi E, Giesel FL, Holland-Letz T, Linhart HG, Eder M (2015). The diagnostic value of PET/CT imaging with the (68)Ga-labelled PSMA ligand HBED-CC in the diagnosis of recurrent prostate cancer. Eur J Nucl Med Mol Imaging.

[CR2] Bander NH, Milowsky MI, Nanus DM, Kostakoglu L, Vallabhajosula S, Goldsmith SJ (2005). Phase I trial of 177Lutetium-labeled J591, a monoclonal antibody to prostate-specific membrane antigen, in patients with androgen-independent prostate Cancer. J Clin Oncol.

[CR3] Barinka C, Novakova Z, Hin N, Bim D, Ferraris DV, Duvall B (2019). Structural and computational basis for potent inhibition of glutamate carboxypeptidase II by carbamate-based inhibitors. Bioorg Med Chem.

[CR4] Barinka C, Rinnova M, Sacha P, Rojas C, Majer P, Slusher BS (2002). Substrate specificity, inhibition and enzymological analysis of recombinant human glutamate carboxypeptidase II. J Neurochem.

[CR5] Barinka C, Rojas C, Slusher B, Pomper M (2012). Glutamate Carboxypeptidase II in diagnosis and treatment of neurologic disorders and prostate Cancer. Curr Med Chem.

[CR6] Barrett JA, Coleman RE, Goldsmith SJ, Vallabhajosula S, Petry NA, Cho S (2013). First-in-man evaluation of 2 high-affinity PSMA-avid Small molecules for imaging prostate Cancer. J Nucl Med.

[CR7] Baum RP, Langbein T, Singh A, Shahinfar M, Schuchardt C, Volk GF (2018). Injection of Botulinum toxin for preventing salivary gland toxicity after PSMA Radioligand therapy: an empirical proof of a promising concept. Nucl Med Mol Imaging.

[CR8] Behr SC, Aggarwal R, VanBrocklin HF, Flavell RR, Gao K, Small EJ (2019). Phase I study of CTT1057, an 18F-labeled imaging agent with Phosphoramidate Core targeting prostate-specific membrane antigen in prostate Cancer. J Nucl Med.

[CR9] Benesova M, Schafer M, Bauder-Wust U, Afshar-Oromieh A, Kratochwil C, Mier W (2015). Preclinical evaluation of a tailor-made DOTA-conjugated PSMA inhibitor with optimized linker moiety for imaging and Endoradiotherapy of prostate Cancer. J Nucl Med.

[CR10] Chakravarty R, Siamof CM, Dash A, Cai W (2018). Targeted α-therapy of prostate cancer using radiolabeled PSMA inhibitors: a game changer in nuclear medicine. Am J Nucl Med Mol Imaging.

[CR11] Cimadamore A, Scarpelli M, Montironi R, Cheng M, Santoni M, Battelli N (2018). New prostate Cancer targets for diagnosis, imaging, and therapy: focus on prostate-specific membrane antigen. Front Oncol.

[CR12] Denmeade SR, Mhaka AM, Rosen DM, Brennen WN, Dalrymple S, Dach I (2012). Engineering a prostate-specific membrane antigen-activated tumor endothelial cell prodrug for cancer therapy. Sci Transl Med.

[CR13] Dude I, Zhang Z, Rousseau J, Hundal-Jabal N, Colpo N, Merkens H (2017). Evaluation of agonist and antagonist radioligands for somatostatin receptor imaging of breast cancer using positron emission tomography. EJNMMI Radiopharmacy Chem.

[CR14] Eppard E, de la Fuente A, Benešová M, Khawar A, Bundschuh RA, Gärtner FC (2017). Clinical translation and first in-human use of [(44)Sc]Sc-PSMA-617 for PET imaging of metastasized castrate-resistant prostate Cancer. Theranostics..

[CR15] Ferraris DV, Shukla K, Tsukamoto T (2012). Structure-activity relationships of glutamate carboxypeptidase II (GCPII) inhibitors. Curr Med Chem.

[CR16] Ghosh A, Heston WDW (2004). Tumor target prostate specific membrane antigen (PSMA) and its regulation in prostate cancer. J Cell Biochem.

[CR17] Ghosh AK, Brindisi M (2015). Organic Carbamates in drug design and medicinal chemistry. J Med Chem.

[CR18] Giesel FL, Will L, Lawal I, Lengana T, Kratochwil C, Vorster M (2018). Intraindividual comparison of 18F-PSMA-1007 and 18F-DCFPyL PET/CT in the prospective evaluation of patients with newly diagnosed prostate carcinoma: a pilot study. J Nucl Med.

[CR19] Herr RJ (2002). 5-substituted-1H-tetrazoles as carboxylic acid isosteres: medicinal chemistry and synthetic methods. Bioorg Med Chem.

[CR20] Herrmann K, Bluemel C, Weineisen M, Schottelius M, Wester H-J, Czernin J (2015). Biodistribution and radiation dosimetry for a probe targeting prostate-specific membrane antigen for imaging and therapy. J Nucl Med.

[CR21] Horoszewicz JS, Kawinski E, Murphy GP (1987). Monoclonal antibodies to a new antigenic marker in epithelial prostatic cells and serum of prostatic cancer patients. Anticancer Res.

[CR22] https://clinicaltrials.gov/ct2/results?cond=Prostate+Cancer&term=PSMA&cntry=&state=&city=&dist=. Accessed on 23 Sept 2020.

[CR23] https://clinicaltrials.gov/ct2/show/NCT03511664?term=PSMA&cond=Prostate+Cancer&phase=2&draw=5&rank=2. Accessed on 23 Sept 2020.

[CR24] Hyun S-H, Kim H-K, Kim J-M, Thompson DH (2010). Oriented insertion of phi29 N-Hexahistidine-tagged gp10 connector protein assemblies into C20BAS Bolalipid membrane vesicles. J Am Chem Soc.

[CR25] Israeli RS, Powell CT, Corr JG, Fair WR, Heston WDW (1994). Expression of the prostate-specific membrane antigen. Cancer Res.

[CR26] Klein Nulent TJW, Valstar MH, de Keizer B, Willems SM, Smit LA, Al-Mamgani A (2018). Physiologic distribution of PSMA-ligand in salivary glands and seromucous glands of the head and neck on PET/CT. Oral Surg Oral Med Oral Pathol Oral Radiol.

[CR27] Knedlik T, Vorlova B, Navratil V, Tykvart J, Sedlak F, Vaculin S (2017). Mouse glutamate carboxypeptidase II (GCPII) has a similar enzyme activity and inhibition profile but a different tissue distribution to human GCPII. FEBS Open Bio.

[CR28] Kozikowski AP, Zhang J, Nan F, Petukhov PA, Grajkowska E, Wroblewski JT (2004). Synthesis of urea-based inhibitors as active site probes of glutamate Carboxypeptidase II: efficacy as analgesic agents. J Med Chem.

[CR29] Kratochwil C, Bruchertseifer F, Giesel FL, Weis M, Verburg FA, Mottaghy F (2016). 225Ac-PSMA-617 for PSMA-targeted α-radiation therapy of metastatic castration-resistant prostate Cancer. J Nucl Med.

[CR30] Kratochwil C, Bruchertseifer F, Rathke H, Bronzel M, Apostolidis C, Weichert W (2017). Targeted α-therapy of metastatic castration-resistant prostate Cancer with 225Ac-PSMA-617: Dosimetry estimate and empiric dose finding. J Nucl Med.

[CR31] Kratochwil C, Bruchertseifer F, Rathke H, Hohenfellner M, Giesel FL, Haberkorn U (2018). Targeted α-therapy of metastatic castration-resistant prostate Cancer with 225Ac-PSMA-617: swimmer-plot analysis suggests efficacy regarding duration of tumor control. J Nucl Med.

[CR32] Kratochwil C, Giesel FL, Stefanova M, Benešová M, Bronzel M, Afshar-Oromieh A (2016). PSMA-targeted radionuclide therapy of metastatic castration-resistant prostate Cancer with 177Lu-labeled PSMA-617. J Nucl Med.

[CR33] Kularatne SA, Zhou Z, Yang J, Post CB, Low PS (2009). Design, synthesis, and preclinical evaluation of prostate-specific membrane antigen targeted 99mTc-Radioimaging agents. Mol Pharm.

[CR34] Lassalas P, Gay B, Lasfargeas C, James MJ, Tran V, Vijayendran KG (2016). Structure property relationships of carboxylic acid Isosteres. J Med Chem.

[CR35] Lopes AD, Davis WL, Rosenstraus MJ, Uveges AJ, Gilman SC (1990). Immunohistochemical and pharmacokinetic characterization of the site-specific Immunoconjugate CYT-356 derived from Antiprostate monoclonal antibody 7E11-C5. Cancer Res.

[CR36] Lu G, Maresca KP, Hillier SM, Zimmerman CN, Eckelman WC, Joyal JL (2013). Synthesis and SAR of 99mTc/re-labeled small molecule prostate specific membrane antigen inhibitors with novel polar chelates. Bioorg Med Chem Lett.

[CR37] Mathias LJ (1979). Esterification and alkylation reactions employing isoureas. Synthesis..

[CR38] Maurer T, Eiber M, Schwaiger M, Gschwend JE (2016). Current use of PSMA–PET in prostate cancer management. Nat Rev Urol.

[CR39] Mhaka A, Gady AM, Rosen DM, Lo K-M, Gillies SD, Denmeade SR (2004). Use of methotrexate-based peptide substrates to characterize the substrate specificity of prostate-specific membrane antigen (PSMA). Cancer Biol Ther.

[CR40] Mosayebnia M, Rezaeianpour S, Rikhtechi P, Hajimahdi Z, Beiki D, Kobarfard F (2018). Novel and efficient method for solid phase synthesis of urea-containing peptides targeting prostate specific membrane antigen (PSMA) in comparison with current methods. Iran J Pharm Res.

[CR41] Oh SW, Wurzer A, Wester H-J, Teoh EJ, Oh S, Langbein T (2019). Quantitative and Qualitative Analyses of Biodistribution and PET Image Quality of Novel Radiohybrid PSMA, (18)F- rhPSMA-7, in Patients with Prostate Cancer. J Nucl Med.

[CR42] O'Keefe DS, Bacich DJ, Heston WDW (2004). Comparative analysis of prostate-specific membrane antigen (PSMA) versus a prostate-specific membrane antigen-like gene. Prostate.

[CR43] Plechanovova A, Byun Y-J, Alquicer G, Skultetyova L, Mlcochova P, Nemcova A (2011). Novel substrate-based inhibitors of human glutamate Carboxypeptidase II with enhanced Lipophilicity. J Med Chem.

[CR44] Rahbar K, Ahmadzadehfar H, Kratochwil C, Haberkorn U, Schäfers M, Essler M (2017). German multicenter study investigating 177Lu-PSMA-617 Radioligand therapy in advanced prostate Cancer patients. J Nucl Med.

[CR45] Rathke H, Kratochwil C, Hohenberger R, Giesel FL, Bruchertseifer F, Flechsig P (2019). Initial clinical experience performing sialendoscopy for salivary gland protection in patients undergoing 225Ac-PSMA-617 RLT. Eur J Nucl Med Mol Imaging.

[CR46] Ristau BT, Bacich DJ, O'Keefe DS (2014). The prostate-specific membrane antigen: lessons and current clinical implications from 20 years of research. Urol Oncol.

[CR47] Robu S, Schmidt A, Eiber M, Schottelius M, Günther T, Hooshyar Yousefi B (2018). Synthesis and preclinical evaluation of novel (18)F-labeled Glu-urea-Glu-based PSMA inhibitors for prostate cancer imaging: a comparison with (18)F-DCFPyl and (18)F-PSMA-1007. EJNMMI Res.

[CR48] Rousseau E, Lau J, Kuo H-T, Zhang Z, Merkens H, Hundal-Jabal N (2018). Monosodium glutamate reduces 68Ga-PSMA-11 uptake in salivary glands and kidneys in a preclinical prostate Cancer model. J Nucl Med.

[CR49] Rowe SP, Gage KL, Faraj SF, Macura KJ, Cornish TC, Gonzalez-Roibon N (2015). 18F-DCFBC PET/CT for PSMA-based detection and characterization of primary prostate Cancer. J Nucl Med.

[CR50] Roy J, Warner BM, Basuli F, Zhang X, Wong K, Pranzatelli T (2020). Comparison of prostate-specific membrane antigen expression levels in human salivary glands to non-human Primates and rodents. Cancer Biother Radiopharm.

[CR51] Rupp NJ, Umbricht CA, Pizzuto DA, Lenggenhager D, Topfer AT, Muller JM (2019). First clinicopathologic evidence of a non-PSMA-related uptake mechanism for 68Ga-PSMA-11 in salivary glands. J Nucl Med.

[CR52] Schmidt A, Wirtz M, Färber SF, Osl T, Beck R, Schottelius M (2018). Effect of Carbohydration on the Theranostic tracer PSMA I&T. ACS Omega.

[CR53] Shin I, Lee M-R, Lee J, Jung M, Lee W, Yoon J (2000). Synthesis of optically active Phthaloyl d-Aminooxy acids from l-amino acids or l-Hydroxy acids as building blocks for the preparation of Aminooxy peptides. J Org Chem.

[CR54] Sosabowski JK, Mather SJ (2006). Conjugation of DOTA-like chelating agents to peptides and radiolabeling with trivalent metallic isotopes. Nat Protoc.

[CR55] Tagawa ST, Milowsky MI, Morris M, Vallabhajosula S, Christos P, Akhtar NH (2013). Phase II study of Lutetium-177–labeled anti-prostate-specific membrane antigen monoclonal antibody J591 for metastatic castration-resistant prostate Cancer. Clin Cancer Res.

[CR56] Tönnesmann R, Meyer PT, Eder M, Baranski A-C (2019). [(177)Lu]Lu-PSMA-617 Salivary Gland Uptake Characterized by Quantitative In Vitro Autoradiography. Pharmaceuticals (Basel).

[CR57] Troyer JK, Beckett ML, Wright GL Jr. Detection and characterization of the prostate-specific membrane antigen (PSMA) in tissue extracts and body fluids. Int J Cancer. 1995;62(5):552–8. 10.1002/ijc.2910620511 7665226

[CR58] Vallabhajosula S, Nikolopoulou A, Babich JW, Osborne JR, Tagawa ST, Lipai I (2014). 99mTc-labeled Small-molecule inhibitors of prostate-specific membrane antigen: pharmacokinetics and biodistribution studies in healthy subjects and patients with metastatic prostate Cancer. J Nucl Med.

[CR59] van Kalmthout L, Braat A, Lam M, van Leeuwaarde R, Krijger G, Ververs T (2019). First experience with 177Lu-PSMA-617 therapy for advanced prostate Cancer in the Netherlands. Clin Nucl Med.

[CR60] van Kalmthout LWM, Lam MGEH, de Keizer B, Krijger GC, Ververs TFT, de Roos R (2018). Impact of external cooling with icepacks on (68)Ga-PSMA uptake in salivary glands. EJNMMI Res.

[CR61] Vāvere AL, Biddlecombe GB, Spees WM, Garbow JR, Wijesinghe D, Andreev OA (2009). A novel Technology for the Imaging of acidic prostate tumors by positron emission tomography. Cancer Res.

[CR62] Wang H, Byun Y, Barinka C, Pullambhatla M, Bhang H-EC, Fox JJ (2010). Bioisosterism of urea-based GCPII inhibitors: synthesis and structure–activity relationship studies. Bioorg Med Chem Lett.

[CR63] Weineisen M, Schottelius M, Wester H-J, Simecek J, Baum RP, Kulkarni HR (2015). 68Ga- and 177Lu-labeled PSMA I&T: optimization of a PSMA-targeted Theranostic concept and first proof-of-concept human studies. J Nucl Med.

[CR64] Weineisen M, Simecek J, Schottelius M, Schwaiger M, Wester H-J (2014). Synthesis and preclinical evaluation of DOTAGA-conjugated PSMA ligands for functional imaging and endoradiotherapy of prostate cancer. EJNMMI Res.

[CR65] Wolf P, Freudenberg N, Bühler P, Alt K, Schultze-Seemann W, Wetterauer U (2010). Three conformational antibodies specific for different PSMA epitopes are promising diagnostic and therapeutic tools for prostate cancer. Prostate.

[CR66] Wurzer A, Di Carlo D, Schmidt A, Beck R, Eiber M, Schwaiger M (2020). Radiohybrid ligands: a novel tracer concept exemplified by 18F- or 68Ga-labeled rhPSMA inhibitors. J Nucl Med.

[CR67] Yang X, Mease RC, Pullambhatla M, Lisok A, Chen Y, Foss CA (2016). [18F]Fluorobenzoyllysinepentanedioic acid Carbamates: new scaffolds for positron emission tomography (PET) imaging of prostate-specific membrane antigen (PSMA). J Med Chem.

[CR68] Yilmaz B, Nisli S, Ergul N, Gursu RU, Acikgoz O, Çermik TF (2019). Effect of external cooling on 177Lu-PSMA uptake by the parotid glands. J Nucl Med.

[CR69] Zechmann CM, Afshar-Oromieh A, Armor T, Stubbs JB, Mier W, Hadaschik B (2014). Radiation dosimetry and first therapy results with a 124I/131I-labeled small molecule (MIP-1095) targeting PSMA for prostate cancer therapy. Eur J Nucl Med Mol Imaging.

[CR70] Zhang D-W, Luo Z, Liu G-J, Weng L-H (2009). α N-O turn induced by fluorinated α-aminoxy diamide: synthesis and conformational studies. Tetrahedron..

[CR71] Zhou Y, Li J, Xu X, Zhao M, Zhang B, Deng S (2019). 64Cu-based radiopharmaceuticals in molecular imaging. Technol Cancer Res Treat.

