# Supplementary material for: Design of PSMA ligands with modifications at the inhibitor part: an approach to reduce the salivary gland uptake of radiolabeled PSMA inhibitors?
Source: EJNMMI Radiopharm Chem. 2021 Feb 26;6:10. doi: 10.1186/s41181-021-00124-1 (PMC7910394; doi:10.1186/s41181-021-00124-1)
Supplement: Supplementary file 1 — Additional file 1. Supporting Information is provided in addition to data presented in the main manuscript portion, including detailed information on all methods for synthesis and analysis as well as on the used instruments. Furthermore, detailed procedures for ligand synthesis, cold metal complexation and radiolabeling are described. Methods for in vitro and in vivo characterizations are given in more detail, as well as radio-RP-HPLC analyses and extraction efficiencies of the metabolite analysis. [file 41181_2021_124_MOESM1_ESM.docx]

**Design of PSMA Ligands with Modifications at the Inhibitor Part: An Approach to Reduce the Salivary Gland Uptake of Radiolabeled PSMA Inhibitors?**

**- Supporting Information -**

Veronika Barbara Felber *, Manuel Amando Valentin, Hans-Jürgen Wester

*Chair of Pharmaceutical Radiochemistry, Technical University of Munich, Garching, Germany;*

**Institutional address of all authors:**

Technical University of Munich,

Chair of Pharmaceutical Radiochemistry,

Walther-Meißner-Str. 3

85748 Garching

GERMANY

***Corresponding author:**

Veronika Barbara Felber

Phone: +49.89.289.12237

Fax: +49.89.289.12204

Email: vroni.felber@tum.de

Keywords: PSMA, GCP II, prostate cancer, radioligand therapy, salivary glands

1. GENERAL INFORMATION

All reagents were purchased from Merck KGaA (Darmstadt, Germany), Sigma-Aldrich Chemie GmbH (Steinheim, Germany), VWR International GmbH (Darmstadt, Germany), TCI (Eschborn, Germany), Iris Biotech (Marktredwitz, Germany) and Carbolution (St. Ingbert, Germany) in the quality grade “for synthesis”. Racemic 2‑PMPA was purchased from Bio‑Techne GmbH (Wiesbaden-Nordenstadt, Germany). Chematech (Dijon, France) delivered the chelator DOTA and derivatives thereof. Cell culture media and buffer solutions were purchased from Merck KGaA (Darmstadt, Germany) and Sigma Aldrich Chemie GmbH (Steinheim, Germany). ([^125^I]NaI was purchased from Hartmann Analytic (Braunschweig, Germany), n.c.a. [^177^Lu]LuCl_3_ was delivered by ITG (Garching, Germany). Solvents were purchased from VWR International GmbH (Darmstadt, Germany) in the quality grade “HPLC grade” and used for column chromatography or liquid-liquid extraction. Dry solvents were purchased from Sigma-Aldrich Chemie GmbH (Steinheim, Germany), Alfa Aesar (Karlsruhe, Germany) and VWR International GmbH (Darmstadt, Germany). Silica gel (high purity grade, 60 Å, 0.040 ‑ 0.063 particle size) used for column chromatography was purchased from Sigma-Aldrich Chemie GmbH (Steinheim, Germany). Solid phase synthesis of the peptides was carried out by manual operation using an Intelli-Mixer syringe shaker (Neolab, Heidelberg, Germany).

1. MATERIALS

*Analytical* and *preparative* RP-HPLC were performed using Shimadzu gradient systems (Shimadzu Deutschland GmbH, Neufahrn, Germany), each equipped with a SPD-20A UV/Vis detector (220 nm, 254 nm). All systems were operated by the LabSolutions software. Prior to quality control acquisitions, a control run was performed, in which only water/acetonitrile (1/1) was injected. Thereby, the system was checked for impurities in the injection port or on the column.

As different eluents and flow rates have been used for several compounds, the used methods are cited in the text and described as follows:

Method A: solvent A = water + 0.1% TFA, solvent B = acetonitrile + 2% water + 0.1% TFA

Method B: solvent A = water + 0.1% TFA, solvent B = acetonitrile + 5% water + 0.1% TFA

Method C: solvent A = water, solvent B: acetonitrile + 5% water

Method D: solvent A = water, solvent B: acetonitrile

*Analytical RP-HPLC* was performed on a Nucleosil 100-C18 (5 μm, 125 mm x 4.6 mm) column (CS GmbH, Langerwehe, Germany) applying different linear solvent gradients (Method A) and a constant flow rate of 1 mL/min. Both, specific gradients and the corresponding retention times t*_R_* as well as capacity factor *k* are cited in the text. The capacity factor was calculated from the experimentally determined dead time (t_0_ = 1.6 min) of the HPLC system and the respective retention time t*_R_*:

$$k=\frac{t_{R}- t_{0}}{t_{0}}$$

*Preparative RP-HPLC* was performed on a Multospher 100 RP C18 (5 μm, 250 x 20 mm) column (CS GmbH, Langerwehe, Germany) applying different linear solvent gradients (Method B or C) and different constant flow rates of 5, 8, 9 or 10 mL/min.

*Flash chromatography* was performed with a Biotage gradient HPLC system (Biotage Europe, Uppsala, Sweden), using HP-Sphere C18-25 catridges (micron spherical silica, Biotage SNAP Ultra C18, 12 g). The compounds were eluted applying different solvent gradients (Method D).

*Radio-RP-HPLC* was performed on a Nucleosil 100-C18 (5 μm, 125 mm x 4.6 mm) column (CS GmbH, Langerwehe, Germany) using a Shimadzu gradient system (Shimadzu Deutschland GmbH, Neufahrn, Germany) with a linear solvent gradient (Method A) and a constant flow rate of 1 mL/min. For radioactivity detection, the outlet of the UV detector was connected to a HERM LB 500 NaI detector (Berthold Technologies, Bad Wildbad, Germany). For metabolite analysis a FlowStar^2^ LB 514 detector (Berthold Technologies, Bad Wildbad, Germany) was additionally connected to the HERM detector.

*Mass spectra* were acquired with an Advion expression L compact mass spectrometer (Advion Ltd., Harlow, UK) with electrospray ionization (positive ion mode) and an orthogonal ion sampling from the heated capillary. The system was operated by the Mass Express software and spectra were processed using the Data Express software.

All *^1^H- and ^13^C-NMR spectra* were measured at room temperature in either DMSO-*d*_6_ or CDCl_3_ on Bruker (Rheinstetten, Germany) instruments (AHV HD-300, AHV HD-400). Chemical shifts (*δ*) are reported in parts per million (ppm) and calibrated on the residual solvent signal (DMSO-*d*_6_: 2.50 ppm for ^1^H and 39.5 ppm for ^13^C, CDCl_3_: 7.26 ppm for ^1^H and 77.0 ppm for ^13^C). Multiplicities are described as follows: s = singlet, d = doublet, t = triplet, q = quartet, br = broad singlet m = multiplet.

*Analytical thin layer chromatography* (TLC) was carried out on precoated silica gel plates form Merck KGaA (Darmstadt, Germany) (TLC plates silica gel 60 F254). Substance spots were visualized either via UV illumination at 254 nm or with a 0.75% (m/v) potassium permanganate stain solution.

*Radio-thin layer chromatography* (TLC) was carried out with a Scan-RAM detector (LabLogic Systems, Sheffield, United Kingdom). Cellulose strips were used for citrate-buffer (0.1 M disodium citrate sesquihydrate in H_2_O). Normal-phase TLC plates (Silica gel 60 RP-18 F_254_s) were used for analyses in NH_4_OAc buffer (1.0 M NH_4_OAc in H_2_O)/DMF (1/1).

*Activity measurements* of the respective probes obtained from competitive binding assays, internalization assays, log D determinations, biodistribution or metabolism studies were measured by a 2480 Wizard^2^ automatic ɣ-counter (PerkinElmer, Waltham, USA).

1. METHODS

General remarks on peptide synthesis

The used equivalents of the reactants for the solid phase synthesis refer to the calculated load after attaching the first amino acid onto the resin. The specific loads are cited in the text. Prior to any reaction, dry resin was swelled in NMP for at least 30 min and then filtered. Unless otherwise indicated, the resin was washed with DMF (6x) after each reaction step. For storage, the resin was washed with DMF (3x) and DCM (3x) and dried in a desiccator.

General procedure for loading the first amino acid onto 2-CTC resin (GP1)

The first amino acid (1.50 eq.) and DIPEA (1.33 eq.) were dissolved in DCM (5.00 mL) and stirred for 5 min at r.t. prior to addition of the resin (1.00 eq.). After 15 min further DIPEA (2.67 eq.) was added and the reaction mixture was stirred for 75 min. Afterwards, MeOH (2 mL) was added and stirred for 15 min. The resin was washed successively with MeOH (4x), DMF (4x) and DCM (4x) and dried at least two hours or overnight in a desiccator. The load was calculated using the following formula:

$$load=\frac{\left( m_{2}-m_{1} \right)\cdot1000}{{(M}_{W}-M_{\mathrm{HCl}}) \cdot m_{2}} \left[ \frac{\mathrm{mmol}}{g} \right]$$

M_W_ = molecular weight of the amino acid [g/mol]

M_HCl_ = molecular weight of HCl [g/mol]

*m_1_ =* mass of dry 2‑CTC resin before coupling [g]

*m_2_ =* mass of dried resin after coupling [g]

General procedure for on-resin peptide bond formation (GP2)

To a solution of TBTU (2.00 eq.), HOAt (2.00 eq.) and the amino acid (2.00 eq.) in DMF (~ 10 mL/g resin), DIPEA (6.00 - 9.00 eq.) was added to adjust the pH value to 9 ‑ 10 and the mixture was allowed to preactivate for five minutes. In the case of Fmoc‑D-Dap(Dde)-OH *sym*‑collidine (6.00 ‑ 8.00 eq.) was added instead of DIPEA. Unless otherwise noted, the solution was added to the resin-coupled peptide and shaken for 2 h at room temperature. Afterwards the resin was washed with DMF (6x).

General procedure for the on-resin Fmoc-removal (GP3)

The resin was shaken 5 × 5 min in 20% piperidine in DMF (v/v) to remove the Fmoc-protective group and afterwards washed with DMF (7x). If Ornithin was the first amino acid bound to the resin, Fmoc-removal was performed 12 x 5min in 20% piperidine.

General procedures for the on-resin Dde-removal (GP4 & GP5)

GP4: If no Fmoc-group was present in the resin bound peptide, the resin was treated with 2% hydrazine in

DMF (10 mL) for 20 min and afterwards washed with DMF (7x).

GP5: If an Fmoc-group was present in the resin bound peptide, the resin was treated with a solution of

imidazole (0.46 g/g resin) and hydroxylamine hydrochloride (0.63 g/g resin) in NMP (5.0 mL/g resin)

and DMF (1.0 mL/g resin) for 2 x 3 h. Afterwards, the resin was washed with DMF (7x).

General procedures for monitoring the reaction progress (GP6 & GP7)

For a test cleavage with TFA (GP6) a small aliquot of the resin was taken and treated with 100 μL of TFA for 15 min at r.t. in an Eppendorf tube.

To avoid cleavage of *tert*-butyl groups or formation of other unidentifiable by-products, test cleavage with HFIP/DCM (GP7) was used. The resin aliquot was treated with 100 μL of HFIP/DCM (1/4, v/v) for 30 min at r.t.

For both procedures the respective solution (without beads!) was transferred into another Eppendorf tube and the solvent was evaporated under a stream of nitrogen. The residue was dissolved in a mixture of H_2_O and MeCN (1/1, v/v), now ready for RP‑HPLC analysis.

Cleaving the peptide off the resin with simultaneous removal of all acid-labile protective groups (GP8)

The resin was treated with TFA/TIPS/DCM (95/2.5/2.5, 10.0 mL) twice for 30 min at r.t. and washed with DCM afterwards (3x). The solvent was evaporated under N_2_ flow and after lyophilisation the crude product was obtained.

General procedure for reactions under air- and moisture free conditions (GP9)

The used Schlenk flask, further glassware as well as the agitator were heated properly three times under vacuum (2.0 × 10^-3^ - 8.0 × 10^-3^ mbar) prior to the reaction. The apparatus was flushed with argon after each heating cycle. Only dry solvents and dry reagents were used for the reactions. The addition of reagents or reactants to the reaction mixture was only performed under argon counterflow. Probes for HPLC control were also only taken under argon counterflow. If the reaction mixture was heated or evolution of gas was expected, the stop cock at the top of the apparatus was replaced by a balloon.

- 1. Synthesis, cold complexation and radiolabeling of ligands with different binding motifs

Reference compound PSMA-10 (**1**) in its free chelator form was synthesized according to a previously published protocol.^(1)^ Preparation of ^nat^Lu‑**1** and [^177^Lu]Lu-**1** followed similar procedures to those conducted within this study.^(2)^ Hence, their analytical data can be found elsewhere and are not again listed. IC_50_ data for ^nat^Lu-**1** as well as internalization, log D, biodistribution and *µ*SPECT/CT data of [^177^Lu]Lu-**1** were not adopted from previously published studies. Instead, they were again determined to ensure a valid comparability of the obtained results and to investigate salivary gland uptake of the reference [^177^Lu]Lu-**1** at 24 h p.i.

- - 1. Synthesis of PSMA derivatives containing modifications within the central Zn^2+^-binding unit
       1. Thioureate 2

*Di-tert-butyl (1H-imidazole-1-carbonothioyl)-L-glutamate (****12****)*

Compound **S-1** was dissolved in dry DCM (14.9 mL) and cooled to 0 °C. Triethylamine (1.79 mL, 12.8 mmol, 2.53 eq.) and DMAP (24.8 mg, 0.20 mmol, 0.04 eq.) were added and stirred for five minutes. Afterwards, 1,1’‑thiocarbonyldiimidazole (**S-2**) (1.36 g, 7.61 mmol, 1.50 eq.) was added, the solution was allowed to warm to room temperature and stirred overnight. The mixture was dissolved with ~ 15 mL DCM and washed with NaHCO_3_ (1x), H_2_O (1x) and brine (1x). The organic layer was dried over Na_2_SO_4_, filtered and the solvent removed under reduced pressure. This afforded 1.67 g (89.2%) of crude product **12** as a yellow oil, which was used without further purification in the next step.

Chemical formula: C_17_H_27_N_3_O_4_S

Molecular weight: 369.48 g/mol

Exact mass: 369.17 g/mol

t*_R_*-value: 13.0 min (10 - 90% B in 15 min, Method A)

Capacity factor *k:* 6.2

ESI-MS: calculated monoisotopic mass (C_17_H_27_N_3_O_4_S): 369.17;

found: ESI (positive ion mode): *m/z*= 370.2 [M(**12**)+H]^+^, 411.2 [M(**12**)+MeCN+H]^+^;

^1^H-NMR (300 MHz, CDCl_3_) δ(ppm): 8.45 (*virt*. d, 1H, H(1)), 7.65 (*virt*. 1H, H(2)), 7.23 (s, 1H, H(3)), 7.18 – 7.01 (m, 1H, H(4)), 4.83 (t, ^3^*J* = 5.7 Hz, 1H, H(5)), 2.56 – 2.44 (m, 2H, H(6)), 2.30 – 2.20 (m, 2H, H(7)), 1.48 (s, 9H, H(8, 8’, 8’’)), 1.45 (s, 9H, H(9, 9’, 9’’)).

*L-Glu-[D-Orn(Dde)-2-CT]-OtBu (****13****)*

Fmoc-D-Orn(Dde)-OH was coupled to 2-CTC resin according to GP1 (load: 0.71 mmol/g, 0.34 mmol, 1.00 eq.). After removal of the Fmoc protective group (GP3), Fmoc-L-Glu-O*t*Bu (**S-3**) (294 mg, 0.69 mmol, 2.00 eq.) was coupled to **18** according to GP2 (2.00 eq. TBTU, 2.00 eq. HOAt, 9.00 eq. DIPEA). After two hours, the Fmoc group of the resin-bound dipeptide was cleaved off, again according to GP3. Some resin beads were taken according to GP6 and analyzed via RP‑HPLC, which indicated nearly complete conversion to **13**.

Chemical formula: C_24_H_39_N_3_O_7_; -*t*Bu: C_20_H_31_N_3_O_7_

Molecular weight: 481.59 g/mol; -*t*Bu: 425.48 g/mol

Exact mass: 481.28 g/mol; -*t*Bu: 425.22 g/mol

t*_R_*-value (-*t*Bu): 8.47 min (10 - 90% B in 15 min, Method A)

Capacity factor *k* (-*t*Bu): 4.6

ESI-MS: calculated monoisotopic mass (C_24_H_39_N_3_O_7_): 481.28, -*t*Bu (C_20_H_31_N_3_O_7_): 425.22;

found: ESI (positive ion mode): *m/z*= 426.0 [M(**13**)-*t*Bu+H]^+^;

*L-Glu(OtBu)_2_-(thiocarbonyl)-L‑Glu-[D-Orn(Dde)-2-CT]-OtBu (****S-4****)*

Reactant **12** (160 mg of crude product, contain ~ 60% of **12** (RP‑HPLC), 0.26 mmol, 1.50 eq.) was dissolved in 2 mL DCM and cooled to 0 °C. Triethylamine (59.6 µL, 0.43 mmol, 2.50 eq.) and the resin-bound dipeptide **13** (0.17 mmol, 1.00 eq) were added and stirred for five minutes at 0 °C. The reaction mixture was heated to 40 °C and stirred overnight under argon atmosphere. The resin was transferred to a syringe for peptide synthesis (equipped with a frit, pore size 25 µm) and washed with DCM (4x). Some resin beads were taken according to GP7 and analyzed via RP‑HPLC, which indicated nearly complete conversion to **S-4**.

Chemical formula: C_38_H_62_N_4_O_11_S;

Molecular weight: 782.99 g/mol;

Exact mass: 782.41 g/mol;

t*_R_*-value: 12.7 min (40 - 100% B in 15 min, Method A)

Capacity factor *k:* 7.5

ESI-MS: calculated monoisotopic mass (C_38_H_62_N_4_O_11_S): 782.41;

found: ESI (positive ion mode): *m/z*= 783.2 [M(**S-4**)+H]^+^, 805.4 [M(**S-4**)+Na]^+^,

822.1 [M(**S-4**)+K]^+^;

*Thioureate* ***2***

Further reactions on resin-bound compound **S-4** were performed according to standard Fmoc‑SPPS on 2‑CT resin, applying the above-mentioned methods (GP2 - GP8). In brief, the Dde protective group was removed (GP4) and succinic anhydride (119 mg, 1.19 mmol, 7.00 eq.) was coupled (GP2) over a period of at least 2.5 h, only using DIPEA (202 µL, 1.19 mmol, 7.00 eq.) and no further coupling reagents. The peptide was elongated with Fmoc‑D‑Lys-O*t*Bu*HCl (157 mg, 0.34 mmol, 2.00 eq.). Therefore, the resin‑bound acid was preactivated for five minutes using TBTU (109 mg, 0.34 mmol, 2.00 eq.), HOAt (46.3 mg, 0.34 mmol, 2.00 eq.) and DIPEA (173 µL, 1.02 mmol, 6.00 eq.). The amino acid was dissolved in DMF, added to the preactivated resin and shaken for at least 2.5 h. Fmoc-removal was conducted (GP3) and Fmoc‑D‑Dap(Dde)‑OH was coupled for at least 2.5 h using TBTU (109 mg, 0.34 mmol, 2.00 eq.), HOAt (46.3 mg, 0.34 mmol, 2.00 eq.) and *sym*-collidine (158 µL, 1.19 mmol, 7.00 eq.) as coupling reagents. Afterwards, Dde-removal was performed according to GP5. The SiFA‑BA moiety (73.4 mg, 0.26 mmol, 1.50 eq.) was attached using TBTU (109 mg, 0.34 mmol, 2.00 eq.), HOAt (46.3 mg, 0.34 mmol, 2.00 eq.) and *sym*-collidine (158 µL, 1.19 mmol, 7.00 eq.) as coupling reagents. After an incubation time of at least 2 h, the Fmoc protective group was removed (GP3) and the DOTA chelator moiety was added to half of the resin. Therefore, DOTA*6 H_2_O (43.6 mg, 85.0 µmol, 1.00 eq.), TBTU (22.8 mg, 71.0 µmol, 0.84 eq.), HOAt (9.66 mg, 71.0 µmol, 0.84 eq.) and *sym*-collidine (66.3 µL, 0.50 mmol, 5.88 eq.) were dissolved in a mixture of DMF/DMSO (5/1, v/v) and incubated with the resin-bound amine for 23 h. As RP-HPLC/MS analysis (GP6) revealed successful coupling, the peptide was cleaved off the resin with TFA/TIPS/H_2_O (95/2.5/2.5, slightly modified to GP8) (2 x 30 min) and purified afterwards by preparative RP-HPLC (30 - 50% B in 20 min, Method B). Subsequent lyophilization afforded 1.99 mg (1.65% yield) of pure product **2** as a colorless powder.

Chemical formula: C_60_H_95_FN_12_O_22_SSi;

Molecular weight: 1415.63 g/mol;

Exact mass: 1414.62 g/mol;

t*_R_*-value: 7.82 min (40 - 100% B in 15 min, Method A)

Capacity factor *k:* 4.2

ESI-MS: calculated monoisotopic mass (C_60_H_95_FN_12_O_22_SSi): 1414.62;

found: ESI (positive ion mode): *m/z*= 708.6 [M(**2**)+2H]^2+^, 1415.8 [M(**2**)+H]^+^;

*^nat^Ga-thioureate* ***2*** *(^nat^Ga-****2****)*

Thioureate **2** (1.99 mg, 1.41 µmol, 1.00 eq.) was dissolved in 300 µL of *t*BuOH, and Ga(NO_3_)_3_*6 H_2_O (1.79 mg, 4.92 µmol, 3.50 eq.) dissolved in 100 µL H_2_O was added. The mixture was stirred for 30 min at 75 °C and afterwards filtered through single use syringe filters (Sartorius Minisart^®^) to remove Ga(OH)_3_ precipitate. The ^nat^Ga‑complex of thioureate **2** (= ^nat^Ga-**2**) was purified by RP‑HPLC (30 - 50% B in 20 min, Method B) and afforded 70.0 µg (3.35%) of pure product ^nat^Ga-**2** as a colorless powder after lyophilization.

Chemical formula: C_60_H_93_FGaN_12_O_22_SSi;

Molecular weight: 1483.33 g/mol;

Exact mass: 1481.53 g/mol;

t*_R_*-value: 8.65 min (10 - 90% B in 15 min, Method A)

Capacity factor *k:* 4.7

ESI-MS: calculated monoisotopic mass (C_60_H_93_FGaN_12_O_22_SSi): 1481.53;

found: ESI (positive ion mode): *m/z*= 742.5 [M(^nat^Ga-**2**)+2H]^2+^, 1483.0 [M(^nat^Ga‑**2**)+H]^+^ ,

1503.6 [M(^nat^Ga‑**2**)+Na]^+^;

- - - 1. Carbamate I (3)

*Tert-butyl (S)-5-oxotetrahydrofuran-2-carboxylate (****S-6****)*

According to a slightly modified procedure by Zhang *et al.*^(3)^, *(S*)-5-oxotetrahydrofuran-2-carboxylic acid (**S‑5**) (1.50 g, 11.5 mmol, 1.00 eq.) was weighed in a 100 mL round bottom flask and dissolved in 36 mL dry DCM. DMAP on polystyrene (1.60 mmol/g, 718 mg, 1.15 mmol, 0.10 eq.) and dry *t*BuOH (1.40 mL, 15.0 mmol, 1.30 eq.) were added and the reaction mixture was cooled to 0 °C. EDC*HCl (2.87 g, 15.0 mmol, 1.30 eq.) in 12 mL dry DCM was added slowly, the ice bath was removed, the solution was allowed to warm to room temperature and stirred under argon atmosphere for 17.3 h. DMAP on polystyrene was filtered off, the organic layer was washed once with H_2_O (spiked with some drops of brine), dried over Na_2_SO_4_ and the solvent was removed under reduced pressure. The crude product was purified by column chromatography (toluene/EtOAc = 3/2) to afford 928 mg (43%) of compound **S-6** as a colorless, crystalline solid.

Chemical formula: C_9_H_14_O_4_;

Molecular weight: 186.21 g/mol;

Exact mass: 186.09 g/mol;

t*_R_*-value: not detectable at 220/254 nm

*R*_f_-value: 0.58 (toluene/EtOAc = 3/2)

^1^H-NMR (300 MHz, CDCl_3_) δ(ppm): 4.83 – 4.76 (m, 1H, H(1)), 2.68 – 2.39 (m,

3H, H(2, 3, 4)), 2.32 – 2.14 (m, 1H, H(5)), 1.48 (s, 9H, H(6, 6’, 6’’)).

^13^C‑NMR (75 MHz, CDCl_3_) δ(ppm): 176.32 (s, 1C, C(1)), 169.12 ((s, 1C, C(2)), 83.23 (s, 1C, C(3)), 76.40 (s, 1C, C(4)), 28.03 (s, 3C, C(5, 5’ 5’’)),

26.90 (s, 1C, C(6)), 26.00 (s, 1C, C(7)).

*(S)-5-(tert-butoxy)-4-hydroxy-5-oxopentanoic acid (****S-7****)*

According to a slightly modified procedure by Zhang *et al.*^(3)^, *tert*-butyl (*S*)-5-oxotetrahydrofuran-2-carboxylate (**S-6**) (425 mg, 2.28 mmol, 1.00 eq.) was dissolved in 2.40 mL THF. At 0 °C, a 1 M aqueous KOH solution (2.64 mL, 2.64 mmol, 1.16 eq.) was added dropwise over five minutes. The solution was allowed to warm to room temperature and stirred for one hour. As reaction control via TLC revealed almost complete consumption of educt **S-6**, THF was removed under reduced pressure and the pH value of the remaining aqueous layer was adjusted to 3 by adding 2 M HCl. The aqueous residue was extracted with EtOAc (3x), the combined organic phases dried over Na_2_SO_4_, filtered and the solvent removed under reduced pressure. This afforded 381 mg of crude product **S-7** as a colorless solid, which was used without further purification in the next step.

Chemical formula: C_9_H_16_O_5_;

Molecular weight: 204.22 g/mol;

Exact mass: 204.10 g/mol;

t*_R_*-value: not detectable at 220/254 nm

*R*_f_-value: 0.0 (toluene/EtOAc = 3/2)

*Di-tert-butyl (S)-2-hydroxypentanedioate (****14****)*

Compound **14** was synthesized in analogy to a previously published procedure by Bergmeier *et al.*^(4)^ with some minor modifications. Glassware and reagents were handled under air- and moisture-free conditions (GP9). Lyophilized educt **S-7** (crude product, ~100 mg, 0.49 mmol, 1.00 eq.) was dissolved in 5 mL dry DCM and the first portion of *O*‑*tert*‑butyl‑*N,N’*‑diisopropylisourea (**S-8**) (162 µL, 0.73 mmol, 1.50 eq.) was added. The reaction mixture was stirred under reflux (~42 °C) and argon atmosphere for 24 h. A second portion of **S-8** (162 µL, 0.73 mmol, 1.50 eq.) was added and also DCM, in order to keep the solvent amount constantly between 3 and 5 mL. After stirring for further 72 h at reflux temperature and under argon atmosphere, the reaction was terminated by diluting the suspension with DCM. Solid by‑products were removed by filtration and the solvent removed under reduced pressure. Purification by column chromatography (PE/EtOAc = 9/1) provided 24.6 mg (19%) of compound **14** as a colorless liquid.

Chemical formula: C_13_H_24_O_5_;

Molecular weight: 260.33 g/mol;

Exact mass: 260.16 g/mol;

t*_R_*-value: not detectable at 220/254 nm

*R*_f_-value: 0.65 (PE/EtOAc = 9/1)

^1^H-NMR (300 MHz, CDCl_3_) δ(ppm): 4.07 (dd, ^3^*J* = 8.0, 4.1 Hz, 1H, H(1)), 2.88 (s, 1H, H(2)), 2.53 – 2.24 (m, 2H, H(3)), 2.08 (*virt*. dddd, 1H, H(4)), 1.93 – 1.73 (m, 1H, H(5)), 1.49 (s, 9H, H(6, 6’, 6’’)), 1.44 (s, 9H, H(7, 7’ 7’’)).

^13^C‑NMR (75 MHz, CDCl_3_) δ(ppm): 174.22 (s, 1C, C(1)), 172.72 (s, 1C, C(2)), 82.80 (s, 1C, C(3)), 80.59 (s, 1C, C(4)), 69.89 (s, 1C, C(5)), 31.13 (s, 1C, C(6)), 29.82 (s, 1C, C(7)), 28.25 (s, 3C, C(8, 8’, 8’’)), 28.17 (s, 3C, C(9, 9’, 9’’)).

*Di-tert-butyl (S)-2-((1H-imidazole-1-carbonyl)oxy)pentanedioate (****S-10****)*

Compound **S-10** was synthesized in analogy to a previously published procedure by Yang et al.^(5)^, with some minor modifications. Di-*tert*-butyl (*S*)-2-hydroxypentanedioate (**14**) (398 mg, 1.53 mmol, 1.00 eq.) was dissolved in 25 mL dry DCM and stirred at room temperature. 1,1’‑Carbonyldiimidazole (**S-9**) (478 mg, 2.95 mmol, 1.93 eq.) was added to the solution, which was then stirred under argon atmosphere for 20 h at room temperature. The reaction mixture was diluted with DCM and washed once with H_2_O. The aqueous layer was extracted two times with DCM, the combined organic phases were dried over Na_2_SO_4_, filtered and the solvent evaporated in vacuo. This afforded 615 mg (>99%) of crude product as slightly yellow solid. RP‑HPLC/MS control revealed nearly complete conversion to product **S-10**, which was used in the next step without further purification.

Chemical formula: C_17_H_26_N_2_O_6_;

Molecular weight: 354.40 g/mol;

Exact mass: 354.18 g/mol;

t*_R_*-value: 10.2 min (40 - 100% B in 15 min, Method A)

Capacity factor *k:* 5.8

ESI-MS: calculated monoisotopic mass (C_17_H_26_N_2_O_6_): 354.18;

found: ESI (positive ion mode): *m/z*= 355.2 [M(**S‑10**)+H]^+^, 396.2 [M(**S‑10**)+MeCN+H]^+^;

*5-Benzyl 1-(tert-butyl) ((((S)-1,5-di-tert-butoxy-1,5-dioxopentan-2-yl)oxy)carbonyl)-L-glutamate (****S-12****)*

Compound **S-12** was synthesized in analogy to a previously published procedure by Weineisen et al.^(6)^ with some minor modifications. Di-*tert*-butyl (*S*)-2-((*1H*-imidazole-1-carbonyl)oxy)pentanedioate (**S-10**) (542 mg, 1.53 mmol, 1.00 eq.) was dissolved in 7.29 mL DCE and cooled to 0 °C. H‑L‑Glu(OBzl)‑OtBu*HCl (**S-11**) (1.01 g, 3.06 mmol, 2.00 eq.) and triethylamine (531 µL, 3.83 mmol, 2.50 eq.) were added and stirred for further five minutes at 0 °C. The reaction mixture was warmed to 45 °C and stirred for 41 h under argon atmosphere. As RP‑HPLC/MS analysis revealed very low conversion (4.4% **S-12**, 44% **S-10**), again, H‑L‑Glu(OBzl)-O*t*Bu*HCl (**S-11**) (1.01 g, 3.06 mmol, 2.00 eq.) and triethylamine (531 µL, 3.83 mmol, 2.50 eq.) were added and the temperature was increased to 55 °C. Complete consumption of educt **S-10** was observed after 70.5 h at 55 °C under argon atmosphere. DCM was added for dilution of the reaction mixture and washed once with H_2_O (+ 1 mL brine). The aqueous phase was extracted two times with DCM, the combined organic layers were dried over Na_2_SO_4_, filtered and the solvent was removed under reduced pressure. The obtained crude product was purified by preparative RP-HPLC (70 ‑ 80% B in 20 min, Method B, 5 mL/min), which gave 277 mg (31.3%) of compound **S-12** as a colorless oil.

Chemical formula: C_30_H_45_NO_10_;

Molecular weight: 579.69 g/mol;

Exact mass: 579.30 g/mol;

t*_R_*-value: 12.6 min (40 - 95% B in 15 min, Method A)

Capacity factor *k:* 7.4

ESI-MS: calculated monoisotopic mass (C_30_H_45_NO_10_): 579.30;

found: ESI (positive ion mode): *m/z*= 412.3 [M(**S-12**)-3*t*Bu+H]^+^, 468.3 [M(**S-12**)‑2*t*Bu+H]^+^,

524.4 [M(**S-12**)‑*t*Bu+H]^+^, 580.5 [M(**S-12**)+H]^+^, 597.5 [M(**S-12**)+H_2_O+H]^+^,

602.5 [M(**S-12**)+Na]^+^, 618.5 [M(**S-12**)+K]^+^;

^1^H-NMR (300 MHz, CDCl_3_) δ(ppm): 7.44 – 7.30 (m, 5H, H(1, 1’, 1’’, 1’’’, 1’’’’), 5.44 (d, ^3^*J* = 8.3 Hz, 1H, H(2)), 5.12 (s, 2H, H(3)), 4.84 (dd, ^3^*J* = 8.1, 4.6 Hz, 1H, H(4)), 4.27 (td, ^3^*J* = 8.2, 4.9 Hz, 1H, H(5)), 2.60 – 2.41 (m, 2H, H(6)), 2.41 – 2.28 (m, 2H, H(7)), 2.28 – 1.82 (m, 4H, H(8, 9)), 1.46 (s, 9H, H(10, 10’, 10’’)), 1.44 (s, 9H, H(11, 11’, 11’’)), 1.44 (s, 9H, H(12, 12’, 12’’)).

^13^C‑NMR (75 MHz, CDCl_3_) δ(ppm): 172.69 (s, 2C, C(1, 2)), 171.94 (s, 1C, C(3)), 170.95 (s, 1C, C(4)), 155.22 (s, 1C, C(5)), 136.02 (s, 1C, C(6)), 128.67 (s, 2C, C(7, 7’)), 128.34 (s, 3C, C(8, 9, 9’)), 82.71 (s, 1C, C(10)), 82.31 (s, 2C, C(11, 12)), 80.75 (s, 1C, C(13)), 66.57 (s, 1C, C(14)), 53.82 (s, 1C, C(15)), 31.24

(s, 1C, C(16)), 30.24 (s, 1C, C(17)), 28.23 (s, 3C, C(18, 18’, 18’’)), 28.12 (s, 3C, C(19, 19’, 19’’)), 28.09 (s, 3C, C(20, 20’, 20’’)), 26.80 (s, 2C, C(21, 22)).

*(S)-5-(tert-butoxy)-4-(((((S)-1,5-di-tert-butoxy-1,5-dioxopentan-2-yl)oxy)carbonyl)amino)-5-oxopentanoic acid (****15****)*

Compound **S-12** (139 mg, 0.24 mmol, 1.00eq.) was dissolved in 12 mL DCM and the solution was flushed 5 minutes with argon to remove dissolved oxygen. 25.5 mg of palladium on carbon (10% wt) (corresponds to 2.55 mg palladium, 24.0 µmol, 0.10 eq.) were added and the flask was sealed with a rubber septum. Remaining air was displaced with argon and subsequently replaced by hydrogen gas. The mixture was stirred under hydrogen atmosphere at room temperature for 16 h. Palladium on carbon was filtered off, and the solvent was evaporated *in vacuo*. This afforded 59.2 mg (50.4%) of compound **15** as a colorless clear oil, which was used in the next step without further purification.

Chemical formula: C_23_H_39_NO_10_;

Molecular weight: 489.56 g/mol;

Exact mass: 489.26 g/mol;

t*_R_*-value: 8.80 min (40 - 95% B in 15 min, Method A)

Capacity factor *k:* 4.9

ESI-MS: calculated monoisotopic mass (C_23_H_39_NO_10_): 489.26;

found: ESI (positive ion mode): *m/z*= 304.2 [M(**15**)-3*t*Bu-OH]^•+^, 322.3 [M(**15**)-3*t*Bu+H]^+^,

378.3 [M(**15**)-2*t*Bu +H]^+^, 434.3 [M(**15**)-*t*Bu +H]^+^, 490.5 [M(**15**)+H]^+^,

507.5 [M(**15**)+H_2_O+H]^+^, 512.5 [M(**15**)+Na]^+^, 528.4 [M(**15**)+K]^+^;

*[S-2-oxopentanedioic acid(OtBu)_2_]-(carbonyl)-L-Glu-[D-Orn(Dde)-2-CT]-OtBu (****S-13****)*

According to GP2, fragment **15** (59.2 mg, 0.12 mmol, 1.10 eq.) was coupled to resin‑bound H‑D‑Orn(Dde) (**18**) (0.11 mmol 1.00 eq.), with TBTU (70.6 mg, 0.22 mmol, 2.00 eq.) and HOAt (29.9 mg, 0.22 mmol, 2.00 eq.) as coupling reagents and *sym*-collidine (131 µL, 0.99 mmol, 9.00 eq.) as base. After shaking for 85 h at room temperature, formation of product **S-13** could be confirmed (GP7). Additionally, some resin beads were taken, treated for 20 minutes with 2% hydrazin/DMF (v/v) and analyzed via RP-HPLC/MS after a test cleavage according to GP7. Thus, stability of the carbamate moiety towards Dde-removal conditions was confirmed, as only one major peak (besides DMF) with the expected m/z-ratio of 604.6 occurred.

Chemical formula: C_38_H_61_N_3_O_13_;

Molecular weight: 767.91 g/mol;

Exact mass: 767.42 g/mol;

t*_R_*-value: 9.21 min (40 - 95% B in 15 min, Method A)

Capacity factor *k*: 5.1

ESI-MS: calculated monoisotopic mass (C_38_H_61_N_3_O_13_): 767.42;

found: ESI (positive ion mode): *m/z*= 768.7 [M(**S-13**)+H]^+^, 790.6 [M(**S-13**)+Na]^+^,

806.6 [M(**S‑13**)+K]^+^;

*Carbamate I (****3****)*

Further reactions on resin-bound compound **S-13** were performed according to standard Fmoc‑SPPS on 2‑CT resin, applying the above-mentioned methods (GP2 - GP8). In brief, the Dde protective group was removed (GP4) and succinic anhydride (77.1 mg, 0.77 mmol, 7.00 eq.) was coupled (GP2) over a period of at least 2.5 h, only using DIPEA (131 µL, 0.77 mmol, 7.00 eq.) and no further coupling reagents. The peptide was elongated with Fmoc‑D‑Lys-O*t*Bu*HCl (101 mg, 0.22 mmol, 2.00 eq.). Therefore, the resin‑bound acid was preactivated for five minutes using TBTU (70.6 mg, 0.22 mmol, 2.00 eq.), HOAt (29.9 mg, 0.22 mmol, 2.00 eq.) and DIPEA (112 µL, 0.66 mmol, 6.00 eq.). The amino acid was dissolved in DMF, added to the preactivated resin and shaken for at least 2.5 h. Fmoc-removal was conducted (GP3) and Fmoc‑D‑Dap(Dde)‑OH was coupled for at least 2.5 h using TBTU (70.6 mg, 0.22 mmol, 2.00 eq.), HOAt (29.9 mg, 0.22 mmol, 2.00 eq.) and *sym*-collidine (102 µL, 0.77 mmol, 7.00 eq.) as coupling reagents. Afterwards, Dde-removal was performed according to GP5. The SiFA‑BA moiety (46.6 mg, 0.17 mmol, 1.50 eq.) was attached using TBTU (70.6 mg, 0.22 mmol, 2.00 eq.), HOAt (29.9 mg, 0.22 mmol, 2.00 eq.) and *sym*-collidine (102 µL, 0.77 mmol, 7.00 eq.) as coupling reagents. After an incubation time of at least 2 h, the Fmoc protective group was removed (GP3) and the DOTA chelator moiety was added to the resin. Therefore, DOTA*6 H_2_O (56.4 mg, 0.11 mmol, 1.00 eq.), TBTU (29.3 mg, 91.3 µmol, 0.83 eq.), HOAt (12.4 mg, 91.3 µmol, 0.83 eq.) and *sym*-collidine (102 µL, 0.77 mmol, 7.00 eq.) were dissolved in a mixture of DMF/DMSO (5/1, v/v) and incubated with the resin-bound amine for 19.5 h. As RP-HPLC/MS analysis (GP6) revealed no sufficient coupling, the resin was divided into two equivalent portions. One portion was again incubated with a freshly prepared DOTA-coupling mixture for 20 h. An only low rise in turnover led to a further DOTA-coupling step, now with DIPEA (101 µL, 0.59 mmol, 10.7 eq.) instead of *sym*‑collidine, in order to monitor and adjust the pH value to 9 ‑ 10. After incubation for 68 h an adequate conversion was achieved and the peptide was cleaved off the resin with TFA/TIPS/H_2_O (95/2.5/2.5, slightly modified to GP8) (2 x 30 min) and purified afterwards by preparative RP‑HPLC (20 - 70% B in 20 min, Method B, 5 mL/min). Subsequent lyophilization afforded 6.30 mg (8.18%) of pure product **3** as a colorless powder. The second portion was cleaved off the resin with HFIP/DCM (1/4, v/v) at room temperature for 1 h in total (2 x 30 min). Thereby, all acid-labile protective groups were retained. The major portion of solvent was removed under a stream of nitrogen and the residual crude product additionally dried by lyophilization. Crude product (48.0 mg, 39.0 µmol, 1.00 eq.) was dissolved in 3.00 mL DMF and DOTA-NHS (32.5 mg, 43.0 µmol, 1.10 eq.) as well as DIPEA (39.7 µL, 0.23 mmol, 6.00 eq.) was added. The mixture was stirred at room temperature for 18 h. Preparative RP-HPLC (35 - 95% B in 15min, Method B, 1 mL/min) and subsequent lyophilization afforded 23.7 mg of the *tert*-butyl-functionalized peptide. Incubation with TFA/TIPS/DCM (95/2.5/2.5, slightly modified to GP8) at 0 °C for 1 h in total, revealed 11.8 mg (21.5%) of pure product **3** after RP-HPLC purification (30 ‑ 50% B in 15 min, Method B, 1 mL/min) and lyophilization. This resulted in an overall yield of 18.1 mg (13.8% yield, chemical purity >98%) of carbamate I (**3**).

Chemical formula: C_60_H_94_FN_11_O_24_Si;

Molecular weight: 1400.55 g/mol;

Exact mass: 1399.62 g/mol;

t*_R_*-value: 8.58 min (10 - 90% B in 15 min, Method A)

Capacity factor *k:* 4.72

ESI-MS: calculated monoisotopic mass (C_60_H_94_FN_11_O_24_Si): 1399.62;

found: ESI (positive ion mode): *m/z*= 701.0 [M(**3**)+2H]^2+^, 1400.9 [M(**3**)+H]^+^,

1868.1 [M_4_(**3**)+3H]^3+^;

*^nat^Ga-carbamate I (^nat^Ga-****3****)*

Carbamate I (**3**) (5.90 mg, 4.22 µmol, 1.00 eq.) was dissolved in 400 µL of HEPES buffer (pH = 3) and Ga(NO_3_)_3_*6 H_2_O (5.37 mg, 14.8 µmol, 3.50 eq.) dissolved in 444 µL HEPES buffer was added. The mixture was stirred for 30 min at 75 °C and afterwards filtered through single use syringe filters (Sartorius Minisart^®^) to remove Ga(OH)_3_ precipitate. The ^nat^Ga‑complex of carbamate I (**3**) (= ^nat^Ga-**3**) was purified by RP‑HPLC (25 - 45% B in 15 min, Method A, 1 mL/min) which afforded 1.64 mg (26.5%) of pure product ^nat^Ga‑**3** as a colorless powder after lyophilization.

Chemical formula: C_60_H_92_FGaN_11_O_24_Si;

Molecular weight: 1468.26 g/mol;

Exact mass: 1466.53 g/mol;

t*_R_*-value: 12.0 min (25 - 45% B in 15 min, Method A)

Capacity factor *k:* 7.0

ESI-MS: calculated monoisotopic mass (C_60_H_92_FGaN_11_O_24_Si): 1466.53;

found: ESI (positive ion mode): *m/z*= 734.9 [M(^nat^Ga-**3**)+2H]^2+^, 1468.0 [M(^nat^Ga‑**3**)+H]^+^,

1835.8 [M_5_(^nat^Ga‑**3**)+4H]^4+^, 1957.3 [M_4_(^nat^Ga‑**3**)+3H]^3+^;

*^nat^Lu-carbamate I (^nat^Lu‑****3****)*

100 µL of the precursor (**3**) (2.00 mM in DMSO, 0.20 µmol, 1.00 eq.) were added to 60.0 µL of LuCl_3_ (20 mM in Tracepur^®^-H_2_O, 1.20 µmol, 6.00 eq.) and 40.0 µL Tracepur^®^-H_2_O. The reaction mixture was heated for 30 min at 70 °C and afforded ^nat^Lu-**3** in 92.3% chemical purity (>99% yield), determined by RP‑HPLC (220 nm). This ^nat^Lu-**3** solution (now 1.00 mM) was directly used as stock solution for affinity determination.

Chemical formula: C_60_H_91_FLuN_11_O_24_Si;

Molecular weight: 1572.49 g/mol;

Exact mass: 1571.54 g/mol;

t*_R_*-value: 8.48 min (10 - 90% B in 15 min, Method A)

Capacity factor *k:* 4.7

ESI-MS: calculated monoisotopic mass (C_60_H_91_FLuN_11_O_24_Si): 1571.54;

found: ESI (positive ion mode): *m/z*= 787.1 [M(^nat^Lu-**3**)+2H]^2+^, 1573.2 [M(^nat^Lu‑**3**)+H]^+^,

1598.0 [M(^nat^Lu‑**3**)+Na]^+^;

*[^177^Lu]Lu-carbamate I ([^177^Lu]Lu^-^****3****)*

5.00 µL of the precursor (**3**) (0.20 mM in DMSO, 1.00 nmol, 1.00 eq.) were added to 10.0 µL of 1 M NaOAc buffer (aq.) (pH = 5.5). Subsequently, 22.3 to 62.2 MBq [^177^Lu]LuCl_3_ (A_s_ > 3000 GBq/mg, 740 MBq/mL, 0.04 M HCl, ITG, Garching, Germany) were added and the mixture was filled up to 100 µL with 0.04 M HCl (in Tracepur^®^-H_2_O). 10.0 µL of 0.1 M sodium ascorbate (aq.) (in Tracepur^®^-H_2_O) were added and the reaction mixture was heated for 25 min at 70 °C. Due to this lower temperature, removal of free [^177^Lu]Lu^3+^ via HLB cartridge (30 mg) was required. This afforded [^177^Lu]Lu-**3** in 69.7 ± 18.2% (n = 6) isolated radiochemical yield (RCY) (decay corrected (d.c.) to the start of synthesis). The apparent molar activities (A_m_) were 12.5 to 21.9 GBq/µmol at the end of synthesis. Radiochemical purity (RCP) as determined by radio-RP-HPLC and radio‑TLC was 96.9 ± 2.1%.

Chemical formula: C_60_H_91_F^177^LuN_11_O_24_Si;

Molecular weight: 1574.47 g/mol;

Exact mass: 1573.54 g/mol;

t*_R_*-value: 9.59 min (10 - 90% B in 15 min, Method A)

t*_R_*-value of co-injected cold standard: 9.49 min (10 - 90% B in 15 min, Method A)

Capacity factor *k:* 5.4

- - - 1. Carbamate II (4)

*5-benzyl 1-(tert-butyl) (S)-2-hydroxypentanedioate (****16****)*

According to a slightly modified procedure by Shin *et al*.^(7)^, (*S*)-5-(*tert*-butoxy)-4-hydroxy-5-oxopentanoic acid (**S-7**) (crude product, 381 mg, 2.28 mmol, 1.00 eq.) was dissolved in 20.7 mL MeOH/H_2_O (10/1, v/v). The solution was stirred during continuous addition of aqueous 10% Na_2_CO_3_, which was terminated as soon as a pH value of 7 was reached (1.10 mL of 10% Na_2_CO_3_ in total). All solvents were removed *in vacuo*, and the remaining residue was further dried by lyophilization, which afforded a slightly yellow solid. For the subsequent step, the used glassware was pretreated as described in GP9 (air- and moisture‑free conditions) and also handling of reactants proceeded by the described methods (GP9). Lyophilized reactant **S-7** was dissolved in 11.4 mL dry DMSO and 5.70 mL thereof (1.14 mmol, 1.00 eq.) were transferred into a Schlenk flask. Benzyl bromide (203 µL, 1.71 mmol, 1.50 eq.) was added and the reaction mixture was stirred at room temperature for 4.5 h under argon atmosphere. Afterwards, the reaction was quenched with H_2_O, the mixture was extracted with Et_2_O (3x). The organic phase was washed once with H_2_O, dried over Na_2_SO_4_, filtered and the solvent removed under reduced pressure. The obtained crude product was purified by preparative RP-HPLC (20 ‑ 80% B in 20 min, Method C, 5 mL/min). Subsequent lyophilization afforded 178 mg (53.2%) of pure product **16** as a colorless solid.

Chemical formula: C_16_H_22_O_5_;

Molecular weight: 294.35 g/mol;

Exact mass: 294.15 g/mol;

t*_R_*-value: 15.7 min (10 - 90% B in 15 min, Method A)

Capacity factor *k:* 9.5

ESI-MS: calculated monoisotopic mass (C_16_H_22_O_5_): 294.15;

found: ESI (positive ion mode): *m/z*= 239.1 [M(**16**)-*t*Bu+H]^+^, 262.2 [M(**16**)-*t*Bu+Na]^+^,

280.2 [M(**16**)-*t*Bu+MeCN+H]^+^, 295.2 [M(**16**)+H]^+^, 317.2 [M(**16**)+Na]^+^,

333.2 [M(**16**)+K]^+^, 358.3 [M(**16**)+MeCN+Na]^+^;

^1^H-NMR (300 MHz, CDCl_3_) δ(ppm): 7.38 – 7.32 (m, 5H, H(1, 1’, 1’’, 1’’’, 1’’’’)), 5.13 (s, 2H, H(2)), 4.09 (dd, ^3^*J* = 7.9, 4.1 Hz, 1H, H(3)), 2.87 (s, 1H, H(4)), 2.63 – 2.39 (m, 2H, H(5)), 2.16 (*virt.* dddd, 1H, H(6)), 1.98 – 1.81 (m, 1H, H(7)), 1.48 (s, 9H, H(8, 8’, 8’’)).

^13^C‑NMR (75 MHz, CDCl_3_) δ(ppm): 174.04 (s, 1C, C(1)), 173.16 (s, 1C, C(2)), 136.06 (s, 1C, C(3)), 128.69 (s, 2C, C(4, 4’)), 128.36 (s, 1C, C(5)), 128.32 (s, 2C, C(6, 6’)), 82.96 (s, 1C, C(7)), 69.73 (s, 1C, C(8)), 66.49 (s, 1C, C(9)), 29.91 (s, 1C, C(10)), 29.63 (s, 1C, C(11)), 28.15 (s, 3C, C(12, 12’, 12’’)).

5-benzyl 1-(tert-butyl) (S)-2-((1H-imidazole-1-carbonyl)oxy)pentanedioate (**S-14**)

Compound **S-14** was synthesized in analogy to a previously published procedure by Yang et al.^(5)^ with some minor modifications. 5-benzyl 1-(tert-butyl) (S)-2-hydroxypentanedioate (**16**) (71.6 mg, 0.24 mmol, 1.00 eq.) was dissolved in 4 mL dry DCM and stirred at room temperature. 1,1’‑Carbonyldiimidazole (**S-9**) (74.5 mg, 0.46 mmol, 1.92 eq.) was dissolved in 1 mL dry DCM and added to the solution, which was stirred under argon atmosphere for 21.3 h at room temperature. The reaction mixture was diluted with DCM and washed once with H_2_O (+ 1 mL brine). The aqueous phase was extracted two times with DCM, the combined organic phases were dried over Na_2_SO_4_, filtered and the solvent evaporated in vacuo. This afforded 112 mg (>99%) of crude product as slightly yellowish oil. RP-HPLC/MS control revealed nearly complete conversion to product **S-14**, which was used in the next step without further purification.

Chemical formula: C_20_H_24_N_2_O_6_;

Molecular weight: 388.42 g/mol;

Exact mass: 388.16 g/mol;

t*_R_*-value: 10.1 min (40 - 100% B in 15 min, Method A)

Capacity factor *k:* 5.7

ESI-MS: calculated monoisotopic mass (C_20_H_24_N_2_O_6_): 388.16;

found: ESI (positive ion mode): *m/z*= 389.2 [M(**S-14**)+H]^+^, 839.5 [M_2_(**S-14**)+MeCN+Na]^+^;

5-benzyl 1-(tert-butyl) (S)-2-((((S)-1,5-di-tert-butoxy-1,5-dioxopentan-2-yl)carbamoyl)oxy)pentanedioate (**S-15**)

Compound **S-15** was synthesized in analogy to a previously published procedure by Weineisen et al.^(6)^ with some minor modifications. 5-benzyl 1-(tert-butyl) (S)-2-((1H-imidazole-1-carbonyl)oxy)pentanedioate (**S‑14**) (93.2 mg, 0.24 mmol, 1.00 eq.) was dissolved in 2.50 mL DCE and cooled to 0 °C. H‑L‑Glu(OtBu)‑OtBu*HCl (**S-1**) (142 mg, 0.48 mmol, 2.00 eq.) and triethylamine (83.2 µL, 0.60 mmol, 2.50 eq.) were added and stirred for further five minutes at 0 °C. The reaction mixture was warmed to 40 °C and stirred for 27 h under argon atmosphere. As RP‑HPLC/MS analysis revealed very low conversion (7% **S‑15**, 90.7% **S-14**), again, H‑L‑Glu(OtBu)-OtBu*HCl (**S-1**) (284 mg, 0.96 mmol, 4.00 eq.) and triethylamine (166 µL, 1.20 mmol, 5.00 eq.) were added and the temperature was increased to 45 °C. Complete consumption of educt **S-14** was observed after 96 h at 45 °C under argon atmosphere. DCM was added for dilution of the reaction mixture and once washed with H_2_O (+ 1 mL brine). The aqueous phase was extracted two times with DCM, the combined organic layers were dried over Na_2_SO_4_, filtered and the solvent was removed under reduced pressure. The obtained crude product was purified by flash chromatography (65 ‑ 98% B in 10 min, Method D, 12 mL/min), which gave 84.1 mg (60.5%) of compound **S-15** as a colorless viscous oil.

Chemical formula: C_30_H_45_NO_10_;

Molecular weight: 579.69 g/mol;

Exact mass: 579.30 g/mol;

t*_R_*-value: 16.9 min (40 - 100% B in 15 min, Method A)

Capacity factor *k:* 10.3

ESI-MS: calculated monoisotopic mass (C_30_H_45_NO_10_): 579.30;

found: ESI (positive ion mode): *m/z*= 602.1 [M(**S-15**)+Na]^+^, 618.2 [M_2_(**S-15**)+K]^+^;

*(S)-5-(tert-butoxy)-4-((((S)-1,5-di-tert-butoxy-1,5-dioxopentan-2-yl)carbamoyl)oxy)-5-oxopentanoic acid (****17****)*

Compound **S-15** (84.1mg, 0.15 mmol, 1.00eq.) was dissolved in 6.20 mL DCM and the solution was flushed 5 minutes with argon to remove dissolved oxygen. 16.0 mg of palladium on carbon (10% wt) (corresponds to 1.60 mg palladium, 15.0 µmol, 0.10 eq.) were added and the flask was sealed with a rubber septum. Remaining air was displaced with argon and subsequently replaced by hydrogen gas. The mixture was stirred under hydrogen atmosphere at room temperature for 24 h. Palladium on carbon was filtered off, and the solvent was evaporated *in vacuo*. This afforded 85.5 mg (>99%) of compound **17** as a colorless clear oil, which was used in the next step without further purification.

Chemical formula: C_23_H_39_NO_10_;

Molecular weight: 489.56 g/mol;

Exact mass: 489.26 g/mol;

t*_R_*-value: 11.6 min (40 - 100% B in 15 min, Method A)

Capacity factor *k:* 6.7

ESI-MS: calculated monoisotopic mass (C_23_H_39_NO_10_): 489.26;

found: ESI (positive ion mode): *m/z*= 304.1 [M(**17**)-3*t*Bu-OH]^•+^, 322.1 [M(**17**)-3*t*Bu+H]^+^,

378.2 [M(**17**)-2*t*Bu+H]^+^, 434.2 [M(**17**)-*t*Bu+H]^+^, 490.4 [M(**17**)+H]^+^, 512.3 [M(**17**)+Na]^+^,

528.4 [M(**17**)+K]^+^;

*L-Glu(OtBu)_2_-(carbonyl)-[(S)-2-oxopentanedioic acid-(D-Orn(Dde)-2-CT)-OtBu] (****S-16****)*

According to GP2, fragment **17** (73.4 mg, 0.15 mmol, 1.00 eq.) was coupled to resin bound H-D‑Orn(Dde) (**18**) with TBTU (110 mg, 0.34 mmol, 2.29 eq.) and HOAt (46.8 mg, 0.34 mmol, 2.29 eq.) as coupling reagents and DIPEA (264 µL, 1.55 mmol, 10.3 eq.) as base. After shaking for 65.3 h at room temperature, formation of product **S-16** could be confirmed (GP6). Additionally, some resin beads were taken, treated for 20 minutes with 2% hydrazin/DMF (v/v) and analyzed via RP-HPLC/MS after test cleavage according to GP7. Thus, stability of the carbamate moiety towards Dde-removal conditions was confirmed, as only one major peak (besides DMF) with the expected m/z-ratio of 604 occurred.

Chemical formula: C_38_H_61_N_3_O_13_, -3*t*Bu: C_26_H_37_N_3_O_13_;

Molecular weight: 767.91 g/mol, -3*t*Bu: 599.59 g/mol;

Exact mass: 767.42 g/mol, -3*t*Bu: 599.23 g/mol;

t*_R_*-value (-3*t*Bu): 10.1 min (10 - 90% B in 15 min, Method A)

Capacity factor *k* (-3*t*Bu): 5.7

ESI-MS: calculated monoisotopic mass (C_38_H_61_N_3_O_13_): 767.42, -3*t*Bu (C_26_H_37_N_3_O_13_): 599.23;

found: ESI (positive ion mode): *m/z*= 600.5 [M(**S-16**)-3*t*Bu+H]^+^,

662.3 [M(**S‑16**)‑3*t*Bu+MeCN+Na]^+^;

*Carbamate II (****4****)*

Further reactions on resin-bound compound **S-16** were performed according to standard Fmoc‑SPPS on 2‑CT resin, applying the above-mentioned methods (GP2 - GP8). In brief, the Dde protective group was removed (GP4) and succinic anhydride (121 mg, 1.20 mmol, 7.00 eq.) was coupled (GP2) over a period of at least 2.5 h, only using DIPEA (156 µL, 0.92 mmol, 5.33 eq.) and no further coupling reagents. The peptide was elongated with Fmoc‑D‑LysO*t*Bu*HCl (159 mg, 0.34 mmol, 2.00 eq.). Therefore, the resin‑bound acid was preactivated for five minutes using TBTU (110 mg, 0.34 mmol, 2.00 eq.), HOAt (46.8 mg, 0.34 mmol, 2.00 eq.) and DIPEA (176 µL, 1.02 mmol, 6.00 eq.). The amino acid was dissolved in DMF, added to the preactivated resin and shaken for at least 2.5 h. Fmoc-removal was conducted (GP3) and Fmoc‑D‑Dap(Dde)‑OH was coupled for at least 2.5 h using TBTU (110 mg, 0.34 mmol, 2.00 eq.), HOAt (46.8 mg, 0.34 mmol, 2.00 eq.) and *sym*-collidine (160 µL, 1.20 mmol, 7.00 eq.) as coupling reagents. Afterwards, Dde-removal was performed according to GP5. The SiFA‑BA moiety (73.4 mg, 0.26 mmol, 1.50 eq.) was attached using TBTU (110 mg, 0.34 mmol, 2.00 eq.), HOAt (46.8 mg, 0.34 mmol, 2.00 eq.) and *sym*-collidine (160 µL, 1.20 mmol, 7.00 eq.) as coupling reagents. After an incubation time of at least 2 h, the Fmoc protective group was removed (GP3) and the DOTA chelator moiety was added to the resin. Therefore, DOTA*6 H_2_O (87.1 mg, 0.17 mmol, 1.00 eq.), TBTU (45.0 mg, 0.14 mmol, 0.83 eq.), HOAt (19.1 mg, 0.14 mmol, 0.83 eq.) and *sym*-collidine (160 µL, 1.20 mmol, 7.00 eq.) were dissolved in a mixture of DMF/DMSO (5/1, v/v) and incubated with the resin-bound amine for 19.5 h. As RP-HPLC/MS analysis (GP6) revealed successful coupling, the peptide was cleaved off the resin with TFA/TIPS/DCM (95/2.5/2.5, GP8) and purified afterwards by preparative RP‑HPLC (35 - 45% B in 20 min, Method B, 5 mL/min). Subsequent lyophilization afforded 8.35 mg (3.51% yield) of pure product **4** as a colorless powder.

Chemical formula: C_60_H_94_FN_11_O_24_Si;

Molecular weight: 1400.55 g/mol;

Exact mass: 1399.62 g/mol;

t*_R_*-value: 13.7 min (10 - 90% B in 15 min, Method A)

Capacity factor *k:* 8.1

ESI-MS: calculated monoisotopic mass (C_60_H_94_FN_11_O_24_Si): 1399.62;

found: ESI (positive ion mode): *m/z*= 701.1 [M(**4**)+2H]^2+^, 1400.6 [M(**4**)+H]^+^;

*^nat^Ga-carbamate II (^nat^Ga-****4****)*

Carbamate II (**4**) (3.22 mg, 2.30 µmol, 1.00 eq.) was dissolved in 200 µL of HEPES buffer (pH = 3) and 260 µL of Ga(NO_3_)_3_*6 H_2_O (2.93 mg, 8.05 µmol, 3.50 eq.) dissolved in HEPES buffer was added. The mixture was stirred for 30 min at 75 °C and afterwards filtered through single use syringe filters (Sartorius Minisart^®^) to remove Ga(OH)_3_ precipitate. The ^nat^Ga‑complex of carbamate II (**4**) (= ^nat^Ga-**4**) was purified by RP‑HPLC (20 - 60% B in 15 min, Method A, 1 mL/min) and afforded 0.53 mg (15.7%) of pure product ^nat^Ga‑**4** as a colorless powder after lyophilization.

Chemical formula: C_60_H_92_FGaN_11_O_24_Si;

Molecular weight: 1468.26 g/mol;

Exact mass: 1466.53 g/mol;

t*_R_*-value: 8.46 min (10 - 90% B in 15 min, Method A)

Capacity factor *k:* 4.6

ESI-MS: calculated monoisotopic mass (C_60_H_92_FGaN_11_O_24_Si): 1466.53;

found: ESI (positive ion mode): *m/z*= 734.3 [M(^nat^Ga-**4**)+2H]^2+^, 1468.2 [M(^nat^Ga‑**4**)+H]^+^,

1835.5 [M_5_(^nat^Ga‑**4**)+4H]^4+^, 1957.4 [M_4_(^nat^Ga‑**4**)+3H]^3+^;

- - 1. Synthesis of proinhibitors I, II & III
       1. Proinhibitor I (5)

*Tert-butyl N^5^-((S)-1-(tert-butoxy)-4-(methylthio)-1-oxobutan-2-yl)-L-glutaminate (****19****)*

Fmoc-L-Glu-O*t*Bu (**S-3**) (3.00 g, 7.05 mmol, 1.10 eq.) was dissolved in 40.1 mL DMF and cooled to 0 °C. COMU (3.02 g, 7.05 mmol, 1.10 eq) and DIPEA (5.27 mL, 31.0 mmol, 4.84 eq.) were added to reach a basic pH of 10. H-L-Met-O*t*Bu*HCl (**S-17**) (1.55 g, 6.41 mmol, 1.00 eq.) was added and the reaction mixture was stirred for further 2 h at 0 °C. The ice bath was removed and the solution was stirred at room temperature for 42 h. The reaction was terminated by addition of H_2_O and diluted with Et_2_O. The aqueous phase was extracted three times with Et_2_O and the combined organic phases were then washed once with saturated NaHCO_3_ and brine. The organic layer was dried over Na_2_SO_4_, filtered and the solvent was removed under reduced pressure. As RP‑HPLC/MS analysis revealed dipeptide Fmoc-**19** to be the main portion of the crude product, 5 mL of 20% piperidine (DMF) were added and stirred for 30 min at room temperature. As the dibenzofulvene‑piperidine by-product could not be removed sufficiently by preparative RP-HPLC (35 ‑ 60% B in 20 min, Method B), it was removed by column chromatography (n‑Hex/EtOAc = 3/2). Afterwards, the solvent was changed (DCM/MeOH/acetone = 5/1/1) by which a distinct separation of product **19** from all remaining by‑products was possible. This afforded 287 mg (11.5%) of pure product **19** as a clear, yellow oil.

Chemical formula: C_18_H_34_N_2_O_5_S;

Molecular weight: 390.54 g/mol;

Exact mass: 390.22 g/mol;

t*_R_*-value: 9.37 min (10 - 90% B in 15 min, Method A)

Capacity factor *k:* 5.2

*R*_f_-value: 0.64 (DCM/MeOH/acetone = 5/1/1)

ESI-MS: calculated monoisotopic mass (C_18_H_34_N_2_O_5_S): 390.22;

found: ESI (positive ion mode): *m/z*= 391.5 [M(**19**)+H]^+^, 782.0 [M_2_(**19**)+H]^+^;

*1-Carbonylimidazole-Glu[D-Orn(Dde)-2-CT]-OtBu (****20****)*

Compound **S‑18**(for the detailed synthesis procedure: see section 3.1.1.1) was transferred into a round bottom flask, where it was dissolved in 3.81 mL DCE. At 0 °C triethylamine (146 µL, 1.05 mmol, 2.50 eq.) and 1,1’‑carbonyldiimidazole (**S-9**) (68.1 mg, 0.42 mmol, 1.00 eq.) were added and the mixture was stirred for further 5 min at 0 °C. Afterwards, it was warmed to 40 °C and stirred for 16 h under argon atmosphere. The resin was washed with DCM (4x) and again dried in a desiccator for 30 min. Some resin beads were taken and treated with HFIP/DCM (1/4) according to GP7. RP‑HPLC/MS analysis revealed nearly complete conversion to product **20**.

Chemical formula: C_28_H_41_N_5_O_8_;

Molecular weight: 575.66 g/mol;

Exact mass: 575.30 g/mol;

t*_R_*-value: 8.24 min (10 - 90% B in 15 min, Method A)

Capacity factor *k:* 4.5

ESI-MS: calculated monoisotopic mass (C_28_H_41_N_5_O_8_): 575.30;

found: ESI (positive ion mode): *m/z*= 452.4 [M(**20**)-imidazole-*t*Bu+H]^+^,

508.5 [M(**20**)‑imidazole]^•+^, 576.6 [M(**20**)+H]^+^;

*L-Glu(L-Met-OtBu)-OtBu-(carbonyl)-L-Glu[D-Orn(Dde)-2-CT]-OtBu (****S-19****)*

Compound **S-19** was synthesized in analogy to a previously published procedure by Weineisen et al.^(6)^ with some minor modifications. The H-L-Glu(L-Met-O*t*Bu)-O*t*Bu dipeptide **19** (246 mg, 0.63 mmol, 1.50 eq.) was dissolved in 3.81 mL DCE and added to compound **20** (0.42 mmol, 1.00 eq.). At 0 °C triethylamine (146 µL, 1.05 mmol, 2.50 eq.) was added and the mixture was stirred for further five minutes at 0 °C. The solution was warmed to 40 °C and stirred for 16 h under argon atmosphere. The resin was transferred to a syringe for peptide synthesis (equipped with a frit, pore size 25 µm) and washed with DCM (4x). Some resin beads were taken according to GP7 and analyzed via RP‑HPLC/MS, which indicated nearly complete conversion to **S‑19**.

Chemical formula: C_43_H_71_N_5_O_13_S;

Molecular weight: 898.12 g/mol;

Exact mass: 897.48 g/mol;

t*_R_*-value: 13.2 min (10 - 90% B in 15 min, Method A)

Capacity factor *k:* 7.8

ESI-MS: calculated monoisotopic mass (C_43_H_71_N_5_O_13_S): 897.48;

found: ESI (positive ion mode): *m/z*= 450.2 [M(**S‑19**)+2H]^2+^, 470.7 [M(**S-19**)+MeCN+2H]^2+^, 899.2 [M(**S‑19**)+H]^+^;

*Proinhibitor I (****5****)*

Further reactions on resin-bound compound **S‑19** were performed according to standard Fmoc‑SPPS on 2‑CT resin, applying the above-mentioned methods (GP2 - GP8). In brief, the Dde protective group was removed (GP4) and succinic anhydride (294 mg, 294 mmol, 7.00 eq.) was coupled (GP2) over a period of at least 2.5 h, only using DIPEA (500 µL, 2.94 mmol, 7.00 eq.) and no further coupling reagents. The peptide was elongated with Fmoc‑D‑Lys-O*t*Bu*HCl (290 mg, 0.63 mmol, 1.50 eq.). Therefore, the resin‑bound acid was preactivated for five minutes using TBTU (270 mg, 0.84 mmol, 2.00 eq.), HOAt (114 mg, 0.82 mmol, 2.00 eq.) and DIPEA (629 µL, 3.70 mmol, 8.81 eq.). The amino acid was dissolved in DMF, added to the preactivated resin and shaken for at least 2.5 h. Fmoc-removal was conducted (GP3) and Fmoc‑D‑Dap(Dde)‑OH (309 mg, 0.63 mmol, 1.50 eq.) was coupled for at least 2.5 h using TBTU (270 mg, 0.84 mmol, 2.00 eq.), HOAt (114 mg, 0.84 mmol, 2.00 eq.) and *sym*-collidine (490 µL, 3.70 mmol, 8.81 eq.) as coupling reagents. Afterwards, Dde-removal was performed according to GP5. Due to extensive oxidation and other side reactions, some test reactions were performed with a minor part (0.09 mmol) of the resin for optimization of SiFA‑BA and DOTA coupling. Hence, further reactions were conducted on the remaining part (0.33 mmol) and all following equivalents refer to this amount of substance. The SiFA‑BA moiety was attached via the Pfp-ester, which was generated previously by preactivation of SiFA-BA (93.1 mg, 0.33 mmol, 1.00 eq.) with pentafluorophenol (104 µL, 0.99 mmol, 3.00 eq.), DIC (153 µL, 0.99 mmol, 3.00 eq.) and pyridine (214 µL, 2.64 mmol, 8.00 eq.) in DMF for 1.5 h. The solution was added to the resin‑bound peptide and incubated for 22 h prior to Fmoc protective group removal (GP3). For coupling of the DOTA moiety, DOTA-NHS (276 mg, 0.36 mmol, 1.10 eq.) and DIPEA (437 µL, 2.57 mmol, 7.79 eq.) were each dissolved in DMF. First, DIPEA in DMF was added to the resin for preactivation. After five minutes, DOTA-NHS in DMF was added and incubated with the resin-bound amine for 21 h. An adequate conversion (RP-HPLC/MS after GP7) was achieved and the peptide was cleaved off the resin under argon atmosphere with TFA/TIPS/DCM/dithiothreitol (95/2.5/2.5/0.5% wt, slightly modified to GP8) (3 x 30 min) and purified afterwards by preparative RP‑HPLC (30 - 60% B in 20 min, Method B and 33 ‑ 40% B in 15 min, Method A). Subsequent lyophilization afforded 0.39 mg (0.08% yield, chemical purity 98.0%) of pure product **5** as a colorless powder.

Chemical formula: C_65_H_104_FN_13_O_24_SSi;

Molecular weight: 1530.76 g/mol;

Exact mass: 1529.68 g/mol;

t*_R_*-value: 8.62 min (10 - 90% B in 15 min, Method A)

Capacity factor *k:* 4.7

ESI-MS: calculated monoisotopic mass (C_65_H_104_FN_13_O_24_SSi): 1529.68;

found: ESI (positive ion mode): *m/z*= 765.8 [M(**5**)+2H]^2+^, 1531.5 [M(**5**)+H]^+^;

*^nat^Lu-proinhibitor I (^nat^Lu‑****5****)*

50 µL of the precursor (**5**) (2.00 mM in DMSO, 0.10 µmol, 1.00 eq.) were added to 30.0 µL of LuCl_3_ (20 mM in Tracepur^®^-H_2_O, 60.0 µmol, 6.00 eq.) and 20.0 µL Tracepur^®^-H_2_O. The reaction mixture was heated for 30 min at 70 °C and afforded ^nat^Lu-**5** in >99% chemical purity (>99% yield), determined by RP‑HPLC (220 nm). This ^nat^Lu-**5** solution (now 0.50 mM) was directly used as stock solution for affinity determination.

Chemical formula: C_65_H_101_FLuN_13_O_24_SSi;

Molecular weight: 1702.70 g/mol;

Exact mass: 1701.60 g/mol;

t*_R_*-value: 8.62 min (10 - 90% B in 15 min, Method A)

Capacity factor *k:* 4.7

ESI-MS: calculated monoisotopic mass (C_65_H_101_FLuN_13_O_24_SSi): 1701.60;

found: ESI (positive ion mode): *m/z*= 851.3 [M(^nat^Lu-**5**)+2H]^2+^, 1701.8 [M(^nat^Lu‑**5**)+H]^+^;

*[^177^Lu]Lu*-*proinhibitor I ([^177^Lu]Lu-****5****)*

5.00 µL of the precursor (**5**) (0.20 mM in DMSO, 1.00 nmol, 1.00 eq.) were added to 10.0 µL of 1 M NaOAc buffer (aq.) (pH = 5.5). Subsequently, 14.0 to 35.9 MBq [^177^Lu]LuCl_3_ (A_s_ > 3000 GBq/mg, 740 MBq/mL, 0.04 M HCl, ITG, Garching, Germany) were added and the mixture was filled up to 100 µL with 0.04 M HCl (in Tracepur^®^-H_2_O). 10.0 µL of 0.1 M sodium ascorbate (aq.) (in Tracepur^®^-H_2_O) were added and the reaction mixture was heated for 25 min at 80 °C. After removal of free [^177^Lu]Lu^3+^ via HLB cartridge (30 mg), [^177^Lu]Lu-**5** was purified by radio‑RP‑HPLC, which afforded [^177^Lu]Lu-**5** in 44.0 ± 7.6% (n = 4) isolated RCY (d.c. to the start of synthesis). Exact apparent A_m_ of products, which were purified by radio-RP-HPLC could not be determined as the amount of cold precursor within the product fraction could only be estimated. In these cases, the amount of substance was roughly determined by the percentage of product, determined by radio-RP-HPLC. This afforded apparent A_m_ of approximately 4.64 to 14.5 GBq/µmol. RCP as determined by radio‑RP‑HPLC and radio‑TLC was 89.3 ± 1.9%. Higher RCP could not be achieved, as right after purification, quality control revealed up to 13% of oxidized byproduct again.

Chemical formula: C_65_H_101_F^177^LuN_13_O_24_SSi;

Molecular weight: 1704.68 g/mol;

Exact mass: 1703.60 g/mol;

t*_R_*-value: 10.1 min (10 - 90% B in 15 min, Method A)

t*_R_*-value of co-injected cold standard: 10.0 min (10 - 90% B in 15 min, Method A)

Capacity factor *k*: 5.7

- - - 1. Proinhibitor II (6)

*(S)-2-((S)-5-(tert-butoxy)-4-(1H-imidazole-1-carboxamido)-5-oxopentanamido)octanoic acid‑[2‑CT] (****S-21****)*

Fmoc-L-2-Aminooctanoic acid was coupled to 2‑CTC resin according to GP1. Further reactions on compound **S-20** (load: 0.72 mmol/g, 0.86 mmol, 1.00 eq.) were performed according to standard Fmoc‑SPPS on 2‑CT resin, applying the above-mentioned methods (GP2 & GP3). In brief, the Fmoc protective group was removed (GP3) and Fmoc‑L‑Glu‑O*t*Bu (549 mg, 1.29 mmol, 1.50 eq.) was coupled (GP2) over a period of 16 h using TBTU (552 mg, 1.72 mmol, 2.00 eq.), HOAt (234 mg, 1.72 mmol, 2.00 eq.) and DIPEA (658 µL, 3.87 mmol, 4.50 eq.). After removal of the Fmoc protective group (GP3), the resin was dried in a desiccator for 30 min and transferred into a round bottom flask, where it was dissolved in 7.81 mL DCE. At 0 °C, triethylamine (298 µL, 2.15 mmol, 2.50 eq.) and 1,1’‑carbonyldiimidazole (154 mg, 0.95 mmol, 1.10 eq.) were added and the mixture was stirred for further 5 min at 0 °C. Afterwards, it was warmed to 40 °C and stirred for 14 h under argon atmosphere. The resin was washed with DCM (4x) and again dried in a desiccator for 30 min. Some resin beads were taken and treated with TFA according to GP7. RP‑HPLC/MS revealed nearly complete conversion to product **S‑21**.

Chemical formula: C_21_H_34_N_4_O_6_;

Molecular weight: 438.53 g/mol;

Exact mass: 438.25 g/mol;

t*_R_*-value: 9.61 min (10 - 90% B in 15 min, Method A)

Capacity factor *k:* 5.4

ESI-MS: calculated monoisotopic mass (C_21_H_34_N_4_O_6_): 438.25;

found: ESI (positive ion mode): *m/z*= 439.3 [M(**S-21**)+H]^+^, 480.4 [M(**S-21**)+MeCN+H]^+^;

*(6S,10S,15S)-6,10-bis(tert-butoxycarbonyl)-15-hexyl-3,8,13-trioxo-1-phenyl-2-oxa-7,9,14-triazahexadecan-16-oic acid (****S-22****)*

Compound **S-22** was synthesized in analogy to a previously published procedure by Weineisen et al.^(6)^ with some minor modifications. Resin-bound **S‑21** (0.86 mmol, 1.00 eq.) was dissolved in 7.81 mL DCE and cooled to 0 °C. H-L‑Glu(OBzl)-OtBu*HCl (**S-11**) (425 mg, 1.29 mmol, 1.50 eq.) and triethylamine (298 µL, 2.15 mmol, 2.50 eq.) were added and stirred for further five minutes at 0 °C. The reaction mixture was warmed to 40 °C and stirred for 4.3 h under argon atmosphere. The resin was transferred to a syringe for peptide synthesis (equipped with a frit, pore size 25 µm) and washed with DCM (4x). Some resin beads were taken according to GP7 and analyzed via RP‑HPLC, which indicated nearly complete conversion to **S-22**. The dried resin was treated with a mixture of HFIP/DCM (1/4, v/v) at room temperature for 4 h in total (4 x 30 min, 2 x 1 h). Thereby, all acid-labile protective groups were retained. The major portion of solvent was removed under a stream of nitrogen and the residual crude product additionally dried by lyophilization. The resulting yellow oil was used in the next step without further purification (81.8 mg, 14.3%).

Chemical formula: C_34_H_53_N_3_O_10_;

Molecular weight: 663.81 g/mol;

Exact mass: 663.37 g/mol;

t*_R_*-value: 15.7 min (10 - 90% B in 15 min, Method A)

Capacity factor *k:* 9.5

ESI-MS: calculated monoisotopic mass (C_30_H_45_NO_10_): 663.37;

found: ESI (positive ion mode): *m/z*= 552.4 [M(**S-22**)-2*t*Bu+H]^+^, 608.5 [M(**S-22**)-*t*Bu+H]^+^,

664.6 [M(**S-22**)+H]^+^;

*5-benzyl 1-(tert-butyl) (((S)-1-(tert-butoxy)-5-(((S)-1-(tert-butoxy)-1-oxooctan-2-yl)amino)-1,5-dioxo-pentan-2-yl)carbamoyl)-L-glutamate (****S-23****)*

Compound **S-23** was synthesized in analogy to a previously published procedure by Bergmeier *et al.*^(4)^ with some minor modifications. Glassware and reagents were handled under air- and moisture-free conditions (GP9). Lyophilized educt **S-22** (crude product, ~81.8 mg, 0.12 mmol, 1.00 eq.) was dissolved in 2 mL dry DCM and the first portion of O‑*tert*‑butyl‑*N,N’*‑diisopropylisourea (**S-8**) (40.1 µL, 0.18 mmol, 1.50 eq.) was added. The reaction mixture was stirred under reflux (~42 °C) and argon atmosphere for 22 h. A second portion of **S-8** (200 µL, 0.90 mmol, 7.49 eq.) was added and also DCM, in order to keep the solvent amount constantly between 2 and 3 mL. After stirring for further 24 h at reflux temperature and under argon atmosphere, the reaction was terminated by diluting the suspension with DCM. Solid by‑products were removed by filtration and the organic layer was washed once with H_2_O. The aqueous phase was extracted twice with DCM. The combined organic phases were transferred into a round bottom flask and the solvent removed under reduced pressure. Purification by preparative RP-HPLC (70 - 90% B in 20 min, Method C, 5 mL/min) provided 41.4 mg (48%) of compound **S-23** as a colorless solid.

Chemical formula: C_38_H_61_N_3_O_10_;

Molecular weight: 719.92 g/mol;

Exact mass: 719.44 g/mol;

t*_R_*-value: 19.2 min (10 - 90% B in 15 min, Method A)

Capacity factor *k:* 11.8

ESI-MS: calculated monoisotopic mass (C_38_H_61_N_3_O_10_): 719.44;

found: ESI (positive ion mode): *m/z*= 720.6 [M(**S-23**)+H]^+^, 742.6 [M(**S-23**)+Na]^+^,

758.6 [M(**S‑23**)+K]^+^;

*(5S,10S,14S)-10,14-bis(tert-butoxycarbonyl)-5-hexyl-2,2-dimethyl-4,7,12-trioxo-3-oxa-6,11,13-triazaheptadecan-17-oic acid (****21****)*

Compound **S-23** (41.4 mg, 57.5 µmol, 1.00 eq.) was dissolved in 6 mL DCM and the solution was flushed five minutes with argon to remove dissolved oxygen. 6.12 mg of palladium on carbon (10% wt) (corresponds to 0.61 mg palladium, 5.75 µmol, 0.10 eq.) were added and the flask was sealed with a rubber septum. Remaining air was displaced with argon and subsequently replaced by hydrogen gas. The mixture was stirred under hydrogen atmosphere at room temperature for 24 h. Palladium on carbon was filtered off, and the solvent was evaporated *in vacuo*. This afforded 35.5 mg (98.1%) of compound **21** as a colorless clear oil, which was used in the next step without further purification.

Chemical formula: C_31_H_55_N_3_O_10_;

Molecular weight: 629.79 g/mol;

Exact mass: 629.39 g/mol;

t*_R_*-value: 15.8 min (10 - 90% B in 15 min, Method A)

Capacity factor *k:* 9.5

ESI-MS: calculated monoisotopic mass (C_31_H_55_N_3_O_10_): 629.39;

found: ESI (positive ion mode): *m/z*= 630.5 [M(**21**)+H]^+^, 652.5 [M(**21**)+Na]^+^;

*L-Glu(L‑2‑Aoc-OtBu)-OtBu-(carbonyl)‑L‑Glu[D-Orn(Dde)-2-CT]-OtBu (****S-24****)*

According to GP2, fragment **21** (35.5 mg, 56.4 µmol, 1.00 eq.) was coupled to resin‑bound H‑D‑Orn(Dde) (**18**) (81.2 µmol 1.44 eq.), with TBTU (36.2 mg, 0.11 mmol, 2.00 eq.) and HOAt (15.0 mg, 0.11 mmol, 2.00 eq.) as coupling reagents and *sym*-collidine (67.7 µL, 0.51 mmol, 9.00 eq.) as base. After shaking for 23.5 h at room temperature, formation of product **S-24** could be confirmed (GP7), as only one major peak with the expected m/z-ratio of 908.4 occurred.

Chemical formula: C_46_H_77_N_5_O_13_;

Molecular weight: 908.14 g/mol;

Exact mass: 907.55 g/mol;

t*_R_*-value: 12.7 min (40 - 95% B in 15 min, Method A)

Capacity factor *k*: 7.5

ESI-MS: calculated monoisotopic mass (C_46_H_77_N_5_O_13_): 907.55;

found: ESI (positive ion mode): *m/z*= 454.8 [M(**S-24**)+2H]^2+^, 908.4 [M(**S-24**)+H]^+^;

*Proinhibitor II (****6****)*

Further reactions on resin-bound compound **S-24** were performed according to standard Fmoc‑SPPS on 2‑CT resin, applying the above-mentioned methods (GP2 - GP8). In brief, the Dde protective group was removed (GP4) and succinic anhydride (56.7 mg, 0.57 mmol, 7.00 eq.) was coupled (GP2) over a period of at least 2.5 h, only using DIPEA (96.4 µL, 0.57 mmol, 7.00 eq.) and no further coupling reagents. The peptide was elongated with Fmoc‑D‑Lys-O*t*Bu*HCl (74.7 mg, 0.16 mmol, 2.00 eq.). Therefore, the resin‑bound acid was preactivated for five minutes using TBTU (52.0 mg, 0.16 mmol, 2.00 eq.), HOAt (22.0 mg, 0.16 mmol, 2.00 eq.) and DIPEA (82.7 µL, 0.49 mmol, 6.00 eq.). The amino acid was dissolved in DMF, added to the preactivated resin and shaken for at least 2.5 h. Fmoc-removal was conducted (GP3) and Fmoc‑D‑Dap(Dde)‑OH (79.5 mg, 0.16 mmol, 2.00 eq.) was coupled for at least 2.5 h using TBTU (52.0 mg, 0.16 mmol, 2.00 eq.), HOAt (22.0 mg, 0.16 mmol, 2.00 eq.) and *sym*-collidine (75.2 µL, 0.57 mmol, 7.00 eq.) as coupling reagents. Afterwards, Dde-removal was performed according to GP5. The SiFA‑BA moiety (12.0 mg, 42.5 µmol, 0.53 eq.) was attached using TBTU (52.0 mg, 0.16 mmol, 2.00 eq.), HOAt (22.0 mg, 0.16 mmol, 2.00 eq.) and *sym*-collidine (75.2 µL, 0.57 mmol, 7.00 eq.) as coupling reagents. After an incubation time of at least 2 h, the Fmoc protective group was removed (GP3) and the DOTA chelator moiety was added to the resin. Therefore, DOTA-NHS (67.8 mg, 89.6 µmol, 1.10 eq.) and DIPEA (118 µL, 8.52 mmol, 7.00 eq.) were each dissolved in DMF. First, DIPEA in DMF was added to the resin for preactivation. After five minutes, DOTA-NHS in DMF was added and incubated with the resin-bound amine for 23.2 h. An adequate conversion (RP-HPLC/MS analysis after GP6) was achieved and the peptide was cleaved off the resin with TFA/TIPS/DCM (95/2.5/2.5, GP8) and purified afterwards by preparative RP‑HPLC (40 - 70% C(A) in 20 min). Subsequent lyophilization afforded 20.0 mg (16.0% yield, chemical purity >99%) of pure product **6** as a colorless powder.

Chemical formula: C_68_H_110_FN_13_O_24_Si;

Molecular weight: 1540.78 g/mol;

Exact mass: 1539.75 g/mol;

t*_R_*-value: 9.44 min (10 - 90% B in 15 min, Method A)

Capacity factor *k:* 5.5

ESI-MS: calculated monoisotopic mass (C_68_H_110_FN_13_O_24_Si): 1539.75;

found: ESI (positive ion mode): *m/z*= 770.5 [M(**6**)+2H]^2+^, 1539.9 [M(**6**)+H]^+^,

1924.9 [M_5_(**6**)+4H]^4+^;

*^nat^Lu-proinhibitor II (^nat^Lu‑****6****)*

100 µL of the precursor (**6**) (2.00 mM in DMSO, 0.20 µmol, 1.00 eq.) were added to 60.0 µL of LuCl_3_ (20 mM in Tracepur^®^-H_2_O, 1.20 µmol, 6.00 eq.) and 40.0 µL Tracepur^®^-H_2_O. The reaction mixture was heated for 30 min at 95 °C and afforded ^nat^Lu-**5** in >99% chemical purity (>99% yield), determined by RP‑HPLC (220 nm). This ^nat^Lu-**6** solution (now 1.00 mM) was directly used as stock solution for affinity determination.

Chemical formula: C_68_H_107_FLuN_13_O_24_Si;

Molecular weight: 1712.72 g/mol;

Exact mass: 1711.67 g/mol;

t*_R_*-value: 9.48 min (10 - 90% B in 15 min, Method A)

Capacity factor *k:* 5.3

ESI-MS: calculated monoisotopic mass (C_68_H_107_FLuN_13_O_24_Si): 1711.67;

found: ESI (positive ion mode): *m/z*= 856.4 [M(^nat^Lu-**6**)+2H]^2+^, 1711.6 [M(^nat^Lu‑**6**)+H]^+^;

*[^177^Lu]Lu-proinhibitor II ([^177^Lu]Lu-****6****)*

5.00 µL of the precursor (**6**) (0.20 mM in DMSO, 1.00 nmol, 1.00 eq.) were added to 10.0 µL of 1 M NaOAc buffer (aq.) (pH = 5.5). Subsequently, 28.0 or 32.5 MBq [^177^Lu]LuCl_3_ (A_s_ > 3000 GBq/mg, 740 MBq/mL, 0.04 M HCl, ITG, Garching, Germany) were added and the mixture was filled up to 100 µL with 0.04 M HCl (in Tracepur^®^-H_2_O). 10.0 µL of 0.1 M sodium ascorbate (aq.) (in Tracepur^®^-H_2_O) were added and the reaction mixture was heated for 25 min at 95 °C. Removal of free [^177^Lu]Lu^3+^ via HLB cartridge (30 mg) was required, which afforded [^177^Lu]Lu-**6** in 81.4 ± 6.9% (n = 2) isolated RCY (d.c. to the start of synthesis). The apparent A_m_ were 24.1 and 24.8 GBq/µmol at the end of synthesis. RCP as determined by radio‑RP‑HPLC and radio‑TLC was 98.2 ± 0.1%.

Chemical formula: C_68_H_107_F^177^LuN_13_O_24_Si;

Molecular weight: 1714.70 g/mol;

Exact mass: 1713.67 g/mol;

t*_R_*-value: 11.1 min (10 - 90% B in 15 min, Method A)

t*_R_*-value of co-injected cold standard: 11.0 min (10 - 90% B in 15 min, Method A)

Capacity factor *k*: 6.4

- - - 1. Proinhibitor III (7)

*(S)-2-((S)-2-(((benzyloxy)carbonyl)amino)-5-(tert-butoxy)-5-oxopentanamido)octanoic acid (****S-25****)*

Fmoc-L-2-Aminooctanoic acid was coupled to 2‑CTC resin according to GP1. Further reactions on compound **S‑20** (load: 0.59 mmol/g, 0.63 mmol, 1.00 eq., n = 3) were performed according to standard Fmoc‑SPPS on 2‑CT resin, applying the above-mentioned methods (GP2 & GP3). In brief, the Fmoc protective group was removed (GP3) and Cbz‑L-Glu(O*t*Bu)-OH (321 mg, 0.95 mmol, 1.50 eq.) was coupled (GP2) over a period of 2 h, using TBTU (405 mg, 1.26 mmol, 2.00 eq.), HOAt (171 mg, 1.26 mmol, 2.00 eq.) and DIPEA (582 µL, 3.42 mmol, 5.43 eq.). Some resin beads were taken and treated with TFA according to GP6. RP‑HPLC/MS analysis revealed nearly complete conversion to product **S-25**. The protected dipeptide was cleaved off the resin by incubation with HFIP/DCM (1/4) for 4 h in total (4 x 30 min, 2 x 1 h). Flash chromatography purification (40 ‑ 90% B in 10 min, Method D, 12 mL/min) of the crude product provided 649 mg (72.0%) of compound **S-25** as a colorless, viscous oil.

Chemical formula: C_25_H_38_N_2_O_7_;

Molecular weight: 478.59 g/mol;

Exact mass: 478.27 g/mol;

t*_R_*-value: 14.8 min (10 - 90% B in 15 min, Method A)

Capacity factor *k:* 8.9

ESI-MS: calculated monoisotopic mass (C_25_H_38_N_2_O_7_): 478.27;

found: ESI (positive ion mode): *m/z*= 422.8 [M(**S-25**)-*t*Bu+H]^+^, 478.8 [M(**S-25**)+H]^+^,

500.7 [M(**S-25**)+Na]^+^, 956.8 [M_2_(**S-25**)+H]^+^, 978.8 [M_2_(**S-25**)+Na]^+^, 994.7 [M_2_(**S-25**)+K]^+^;

*Tert-butyl (S)-2-((S)-2-(((benzyloxy)carbonyl)amino)-5-(tert-butoxy)-5-oxopentanamido)octanoate (****S-26****)*

Compound **S-26** was synthesized in analogy to a previously published procedure by Bergmeier *et al.*^(4)^ with some minor modifications. Glassware and reagents were handled under air- and moisture-free conditions (GP9). Lyophilized educt **S-25** (649 mg, 1.36 mmol, 1.00 eq.) was dissolved in 2 mL dry DCM and the first portion of O‑*tert*‑butyl‑*N,N’*‑diisopropylisourea (**S-8**) (454 µL, 2.04 mmol, 1.50 eq.) was added. The reaction mixture was stirred under reflux (~42 °C) and argon atmosphere for 22 h. A second portion of **S‑8** (454 µL, 2.04 mmol, 1.50 eq.) was added and also DCM, in order to keep the solvent amount constantly between 2 and 3 mL. After stirring for further 24 h at reflux temperature and under argon atmosphere, the reaction was terminated by diluting the suspension with DCM. Solid by‑products were removed by filtration and the organic layer washed once with H_2_O. The aqueous phase was extracted twice with DCM. The combined organic phases were transferred into a round bottom flask and the solvent removed under reduced pressure. Purification by flash chromatography (40 - 90% B in 10 min, Method D, 12 mL/min) provided 481 mg (66.2%) of compound **S‑26** as a colorless solid.

Chemical formula: C_29_H_46_N_2_O_7_;

Molecular weight: 534.69 g/mol;

Exact mass: 534.33 g/mol;

t*_R_*-value: 16.7 min (10 - 90% B in 15 min, Method A)

Capacity factor *k:* 10.1

ESI-MS: calculated monoisotopic mass (C_29_H_46_N_2_O_7_): 534.33;

found: ESI (positive ion mode): *m/z*= 423.0 [M(**S‑26**)-2*t*Bu+H]^+^, 479.1 [M(**S‑26**)-*t*Bu+H]^+^, 535.2 [M(**S‑26**)+H]^+^, 557.0 [M(**S‑26**)+Na]^+^, 579.2 [M(**S‑26**)+K]^+^;

*Tert-butyl (S)-2-((S)-2-amino-5-(tert-butoxy)-5-oxopentanamido)octanoate (****S‑27****)*

Compound **S-26** (481 mg, 0.89 mmol, 1.00 eq.) was dissolved in 10 mL DCM and the solution was flushed 5 minutes with argon to remove dissolved oxygen. 94.2 mg of palladium on carbon (10% wt) (corresponds to 9.42 mg palladium, 89.0 µmol, 0.10 eq.) were added and the flask was sealed with a rubber septum. Remaining air was displaced with argon and subsequently replaced by hydrogen gas. The mixture was stirred under hydrogen atmosphere at room temperature for 26 h. Palladium on carbon was filtered off, and the solvent was evaporated *in vacuo*. This afforded 162 mg (45.3%) of compound **S‑27** as a slightly brown viscous oil, which was used in the next step without further purification.

Chemical formula: C_21_H_40_N_2_O_5_;

Molecular weight: 400.56 g/mol;

Exact mass: 400.29 g/mol;

t*_R_*-value: 7.07 min (40 - 95% B in 15 min, Method A)

Capacity factor *k:* 3.7

ESI-MS: calculated monoisotopic mass (C_21_H_40_N_2_O_5_): 400.29;

found: ESI (positive ion mode): *m/z*= 401.0 [M(**S‑27**)+H]^+^, 422.9 [M(**S‑27**)+Na]^+^,

438.9 [M(**S‑27**)+K]^+^, 801.0 [M_2_(**S‑27**)+H]^+^, 822.9 [M_2_(**S‑27**)+Na]^+^;

*5-Benzyl 1-(tert-butyl) (1H-imidazole-1-carbonyl)-L-glutamate (****S-28****)*

Compound **S-11** (396 mg, 1.20 mmol, 1.00 eq.) was dissolved in 10 mL DCE and cooled to 0 °C. Triethylamine (416 µL, 3.00 mmol, 2.50 eq.), DMAP on polystyrene (3.00 mmol/g, 16.0 mg, 48.0 µmol, 0.04 eq.) and 1,1’‑carbonyldiimidazole (**S-9**) (214 mg, 1.32 mmol, 1.10 eq.) were added and the mixture was stirred for further 5 min at 0 °C. Afterwards, it was warmed to room temperature and stirred for 42 h under argon atmosphere. DMAP was filtered off and the solution was washed once with saturated NaHCO_3_, brine and H_2_O. The combined organic phases were dried over Na_2_SO_4_, filtered and the solvent was removed under reduced pressure. The obtained crude product (439 mg, 94.5%) was analyzed via RP‑HPLC/MS and revealed almost exclusively product **S-28**, which was used in the next step without further purification.

Chemical formula: C_20_H_25_N_3_O_5_;

Molecular weight: 387.44 g/mol;

Exact mass: 387.18 g/mol;

t*_R_*-value: 10.1 min (10 - 90% B in 15 min, Method A)

Capacity factor *k:* 5.7

ESI-MS: calculated monoisotopic mass (C_20_H_25_N_3_O_5_): 387.18;

found: ESI (positive ion mode): *m/z*= 388.2 [M(**S-28**)+H]^+^;

*5-Benzyl 1-(tert-butyl) (((S)-5-(tert-butoxy)-1-(((S)-1-(tert-butoxy)-1-oxooctan-2-yl)amino)-1,5-dioxopentan-2-yl)carbamoyl)-L-glutamate (****S-29****)*

Compound **S-29** was synthesized in analogy to a previously published procedure by Weineisen et al.^(6)^ with some minor modifications. The H-L-Glu(OtBu)-L-2-Aoc-OtBu dipeptide **S-27** (162 mg, 0.40 mmol, 1.10 eq.) was dissolved in 10 mL DCE and compound **S-28** (141 mg, 0.36 mmol, 1.00 eq.) was added. At 0 °C, triethylamine (139 µL, 1.00 mmol, 2.78 eq.) was added and the mixture was stirred for further five minutes at 0 °C. The solution was warmed to 40 °C and stirred for 21 h under argon atmosphere. The reaction mixture was washed once with H_2_O and brine. The combined aqueous phases were extracted once with DCM. The entire organic layer was dried over Na_2_SO_4_, filtered and the solvent removed *in vacuo*. Purification of the crude product by preparative RP‑HPLC (45 ‑ 90% B in 20 min, Method B, 5 mL/min) afforded 128 mg (49%) of compound **S-29** as a colorless clear viscous oil.

Chemical formula: C_38_H_61_N_3_O_10_;

Molecular weight: 719.92 g/mol;

Exact mass: 719.44 g/mol;

t*_R_*-value: 17.5 min (40 - 95% B in 15 min, Method A)

Capacity factor *k:* 10.7

ESI-MS: calculated monoisotopic mass (C_38_H_61_N_3_O_10_): 719.44;

found: ESI (positive ion mode): *m/z*= 720.0 [M(**S-29**)+H]^+^, 742.0 [M(**S-29**)+MeCN+H]^+^;

*(5S,8S,12S)-8-(3-(tert-butoxy)-3-oxopropyl)-12-(tert-butoxycarbonyl)-5-hexyl-2,2-dimethyl-4,7,10-trioxo-3-oxa-6,9,11-triazapentadecan-15-oic acid (****22****)*

Compound **S-29** (128 mg, 0.18 mmol, 1.00 eq.) was dissolved in 6 mL DCM and the solution was flushed 5 minutes with argon to remove dissolved oxygen. 19.2 mg of palladium on carbon (10% wt) (corresponds to 1.92 mg palladium, 18.0 µmol, 0.10 eq.) were added and the flask was sealed with a rubber septum. Remaining air was displaced with argon and subsequently replaced by hydrogen gas. The mixture was stirred under hydrogen atmosphere at room temperature for 23 h. Palladium on carbon was filtered off, and the solvent was evaporated *in vacuo*. This afforded 102 mg (89.9%) of compound **22** as a colorless solid, which was used in the next step without further purification.

Chemical formula: C_31_H_55_N_3_O_10_;

Molecular weight: 629.79 g/mol;

Exact mass: 629.39 g/mol;

t*_R_*-value: 13.1 min (40 - 95% B in 15 min, Method A)

Capacity factor *k:* 7.7

ESI-MS: calculated monoisotopic mass (C_31_H_55_N_3_O_10_): 629.39;

found: ESI (positive ion mode): *m/z*= 630.0 [M(**22**)+H]^+^, 651.9 [M(**22**)+Na]^+^,

667.9 [M(**22**)+K]^+^;

*L‑Glu(L‑2-Aoc-OtBu)-OtBu-(carbonyl)-L‑Glu[D-Orn(Dde)-2-CT]-OtBu (****S-30****)*

According to GP2, fragment **22** (102 mg, 0.16 mmol, 1.00 eq.) was coupled to resin‑bound H‑D‑Orn(Dde) (**18**) (0.23 mmol 1.44 eq.), with TBTU (104 mg, 0.32 mmol, 2.00 eq.) and HOAt (44.0 mg, 0.32 mmol, 2.00 eq.) as coupling reagents and *sym*-collidine (191 µL, 1.44 mmol, 9.00 eq.) as base. After shaking for 19 h at room temperature, formation of product **S‑30** could be confirmed (GP7), as only one major peak with the expected m/z-ratio of 908.0 occurred.

Chemical formula: C_46_H_77_N_5_O_13_;

Molecular weight: 908.14 g/mol;

Exact mass: 907.55 g/mol;

t*_R_*-value: 13.7 min (40 - 95% B in 15 min, Method A)

Capacity factor *k*: 8.1

ESI-MS: calculated monoisotopic mass (C_46_H_77_N_5_O_13_): 907.55;

found: ESI (positive ion mode): *m/z*= 908.0 [M(**S‑30**)+H]^+^, 1814.9 [M_2_(**S‑30**)+H]^+^;

*Proinhibitor III (****7****)*

Further reactions on resin-bound compound **S-30** (0.23 mmol, 1.00 eq.) were performed according to standard Fmoc‑SPPS on 2‑CT resin, applying the above-mentioned methods (GP2 - GP8). In brief, the Dde protective group was removed (GP4) and succinic anhydride (161 mg, 1.61 mmol, 7.00 eq.) was coupled (GP2) over a period of at least 2.5 h, only using DIPEA (374 µL, 2.20 mmol, 9.56 eq.) and no further coupling reagents. The peptide was elongated with Fmoc‑D‑LysO*t*Bu*HCl (212 mg, 0.46 mmol, 2.00 eq.). Therefore, the resin‑bound acid was preactivated for five minutes by using TBTU (148 mg, 0.46 mmol, 2.00 eq.), HOAt (63.0 mg, 0.46 mmol, 2.00 eq.) and DIPEA (335 µL, 1.97 mmol, 8.56 eq.). The amino acid was dissolved in DMF, added to the preactivated resin and shaken for at least 2.5 h. Fmoc-removal was conducted (GP3) and Fmoc‑D-Dap(Dde)-OH (226 mg, 0.46 mmol, 2.00 eq.) was coupled for at least 2.5 h using TBTU (148 mg, 0.46 mmol, 2.00 eq.), HOAt (63.0 mg, 0.46 mmol, 2.00 eq.) and *sym*-collidine (213 µL, 1.61 mmol, 7.00 eq.) as coupling reagents. Afterwards, Dde-removal was performed according to GP5. The SiFA‑BA moiety (64.9 mg, 0.23 mmol, 1.00 eq.) was attached using TBTU (148 mg, 0.46 mmol, 2.00 eq.), HOAt (63.0 mg, 0.46 mmol, 2.00 eq.) and *sym*-collidine (213 µL, 1.61 mmol, 7.00 eq.) as coupling reagents and incubation for at least 2 h. Fmoc protective group removal (GP3) was performed followed by coupling of the DOTA moiety. Therefore, DOTA-NHS (175 mg, 0.23 mmol, 1.00 eq.) and DIPEA (324 µL, 1.91 mmol, 8.28 eq.) were each dissolved in DMF. First, DIPEA in DMF was added to the resin for preactivation. After five minutes, DOTA-NHS in DMF was added and incubated with the resin‑bound amine for 16 h. An adequate conversion (RP-HPLC/MS analysis after GP6) was reached and the peptide was cleaved off the resin with TFA/TIPS/DCM (95/2.5/2.5/, GP8) and purified afterwards by preparative RP‑HPLC (40 - 65% B in 20 min, Method B, 5 mL/min). Subsequent lyophilization afforded 16.6 mg (4.68% yield, chemical purity >99%) of pure product **7** as a colorless powder.

Chemical formula: C_68_H_110_FN_13_O_24_Si;

Molecular weight: 1540.78 g/mol;

Exact mass: 1539.75 g/mol;

t*_R_*-value: 9.15 min (10 - 90% B in 15 min, Method A)

Capacity factor *k:* 5.1

ESI-MS: calculated monoisotopic mass (C_68_H_110_FN_13_O_24_Si): 1539.75;

found: ESI (positive ion mode): *m/z*= 770.5 [M(**7**)+2H]^2+^, 1539.9 [M(**7**)+H]^+^;

*^nat^Lu-proinhibitor III (^nat^Lu‑****7****)*

100 µL of the precursor (**7**) (2.00 mM in DMSO, 0.20 µmol, 1.00 eq.) were added to 60.0 µL of LuCl_3_ (20 mM in Tracepur^®^-H_2_O, 1.20 µmol, 6.00 eq.) and 40.0 µL Tracepur^®^-H_2_O. The reaction mixture was heated for 30 min at 95 °C and afforded ^nat^Lu-**7** in >99% chemical purity (>99% yield), determined by RP‑HPLC (220 nm). This ^nat^Lu-**7** solution (now 1.00 mM) was directly used as stock solution for affinity determination.

Chemical formula: C_68_H_107_FLuN_13_O_24_Si;

Molecular weight: 1712.72 g/mol;

Exact mass: 1711.67 g/mol;

t*_R_*-value: 9.29 min (10 - 90% B in 15 min, Method A)

Capacity factor *k:* 5.2

ESI-MS: calculated monoisotopic mass (C_68_H_107_FLuN_13_O_24_Si): 1711.67;

found: ESI (positive ion mode): *m/z*= 856.1 [M(^nat^Lu-**7**)+2H]^2+^, 1711.1 [M(^nat^Lu‑**7**)+H]^+^;

*[^177^Lu]Lu-proinhibitor III ([^177^Lu]Lu-****7****)*

5.00 µL of the precursor (**7**) (0.20 mM in DMSO, 1.00 nmol, 1.00 eq.) were added to 10.0 µL of 1 M NaOAc buffer (aq.) (pH = 5.5). Subsequently, 27.4 MBq [^177^Lu]LuCl_3_ (A_s_ > 3000 GBq/mg, 740 MBq/mL, 0.04 M HCl, ITG, Garching, Germany) were added and the mixture was filled up to 100 µL with 0.04 M HCl (in Tracepur^®^-H_2_O). 10.0 µL of 0.1 M sodium ascorbate (aq.) (in Tracepur^®^-H_2_O) were added and the reaction mixture was heated for 25 min at 95 °C. This afforded [^177^Lu]Lu-**7** in >99% (n = 1) isolated RCY (d.c. to the start of synthesis). The apparent A_m_ was 27.4 GBq/µmol at the end of synthesis. RCP as determined by radio‑RP‑HPLC and radio‑TLC was 94.7%.

Chemical formula: C_68_H_107_F^177^LuN_13_O_24_Si;

Molecular weight: 1714.70 g/mol;

Exact mass: 1713.67 g/mol;

t*_R_*-value: 11.0 min (10 - 90% B in 15 min, Method A)

t*_R_*-value of co-injected cold standard: 10.9 min (10 - 90% B in 15 min, Method A)

Capacity factor *k*: 6.3

- - 1. Synthesis of PSMA-binding motifs with substituents & bioisosteres of the P1’‑γ‑carboxylic acid
       1. 2-Aminoheptanoic acid derivative 8

*Tert-butyl (S)-2-aminoheptanoate (****23****)*

Compound **23** was synthesized in analogy to a previously published procedure by Hyun et al.^(8)^ with some minor modifications. (S)-2-Aminoheptanoic acid (**S-31**) (300 mg, 2.07 mmol, 1.00 eq.) was dissolved in 7.13 mL of tert‑butyl acetate (**S-32**) and 70% (v/v) perchloric acid (aq.) (384 µL, 4.74 mmol, 2.29 eq.) was added slowly. The reaction mixture was stirred for 22 h at room temperature. H_2_O and some drops of 0.3 M HCl were added for quenching. The pH value of the aqueous phase was adjusted to 9 with 10% (w/v) Na_2_CO_3_(aq.) and extracted three times with DCM. The combined organic phases were dried over Na_2_SO_4_, filtered and the solvent was removed under reduced pressure. Purification by column chromatography (DCM/MeOH/acetone = 5/1/1) yielded 446 mg (>99%) of pure product **23** as a slightly yellow viscous oil.

Chemical formula: C_11_H_23_NO_2_;

Molecular weight: 201.31 g/mol;

Exact mass: 201.17 g/mol;

t*_R_*-value: not detectable at 220/254 nm

*R*_f_-value: 0.76 (DCM/MeOH/acetone = 5/1/1)

ESI-MS: calculated monoisotopic mass (C_11_H_23_NO_2_): 201.17;

found: ESI (positive ion mode): *m/z*= 202.0 [M(**23**)+H]^+^, 243.0 [M(23)+MeCN+H]^+^;

^1^H-NMR (300 MHz, CDCl_3_) δ(ppm): 3.75 (t, ^3^*J* = 6.2 Hz, 1H, H(1)), 2.04 (s, 2H, H(2)), 1.91 – 1.70 (m, 2H, H(3)), 1.48 (s, 9H, H(4, 4’, 4’’)), 1.31 (*virt*. td, 6H, H(5, 6, 7)), 0.93 – 0.78 (m, 3H, H(8)).

^13^C‑NMR (75 MHz, CDCl_3_) δ(ppm): 171.02 (s, 1C, C(1)), 83.45 (s, 1C, C(2)), 54.35 (s, 1C, C(3)), 32.18 (s, 1C, C(4)), 31.41 (s, 1C, C(5)), 28.09 (s, 3C, C(6, 6’, 6’’)), 24.56 (s, 1C, C(7)), 22.43 (s, 1C, C(8)), 14.03 (s, 1C, C(9)).

*L-2-Aha-OtBu-(carbonyl)-L‑Glu[D-Orn(Dde)-2-CT]-OtBu (****S-33****)*

Compound **20** (for the detailed synthesis procedure: see section 3.1.2.1) (0.24 mmol, 1.00 eq.) was transferred into a round bottom flask and dissolved in 2.50 mL DCE. At 0 °C, triethylamine (83.2 µL, 0.60 mmol, 2.50 eq.) and *tert-*butyl (*S*)-2-aminoheptanoate (**23**) (60.9 mg, 0.30 mmol, 1.25 eq.) were added and the mixture was stirred for further 5 min at 0 °C. Afterwards, it was warmed to 40 °C and stirred for 16.3 h under argon atmosphere. The resin was washed with DCM (4x), some resin beads were taken and treated with HFIP/DCM (1/4) according to GP7. RP‑HPLC/MS analysis revealed nearly complete conversion to product **S-33**.

Chemical formula: C_36_H_60_N_4_O_10_;

Molecular weight: 708.89 g/mol;

Exact mass: 708.43 g/mol;

t*_R_*-value: 14.2 min (10 - 90% B in 15 min)

Capacity factor *k:* 8.5

ESI-MS: calculated monoisotopic mass (C_36_H_60_N_4_O_10_): 708.43;

found: ESI (positive ion mode): *m/z*= 709.1 [M(**S-33**)+H]^+^, 731.1 [M(**S-33**)+Na]^+^;

*2-Aminoheptanoic acid derivative* ***8***

Further reactions on resin-bound compound **S-33** (0.24 mmol, 1.00 eq.) were performed according to standard Fmoc‑SPPS on 2‑CT resin, applying the above-mentioned methods (GP2 - GP8). In brief, the Dde protective group was removed (GP4) and succinic anhydride (170 mg, 1.68 mmol, 7.00 eq.) was coupled (GP2) over a period of at least 2.5 h, only using DIPEA (286 µL, 1.68 mmol, 7.00 eq.) and no further coupling reagents. The peptide was elongated with Fmoc‑D‑LysO*t*Bu*HCl (221 mg, 0.48 mmol, 2.00 eq.). Therefore, the resin‑bound acid was preactivated for five minutes using TBTU (154 mg, 0.48 mmol, 2.00 eq.), HOAt (65.3 mg, 0.48 mmol, 2.00 eq.) and DIPEA (245 µL, 1.44 mmol, 6.00 eq.). The amino acid was dissolved in DMF, added to the preactivated resin and shaken for at least 2.5 h. Fmoc-removal was conducted (GP3) and Fmoc‑D-Dap(Dde)-OH (235 mg, 0.48 mmol, 2.00 eq.) was coupled for at least 2.5 h using TBTU (154 mg, 0.48 mmol, 2.00 eq.), HOAt (65.3 mg, 0.48 mmol, 2.00 eq.) and *sym*-collidine (223 µL, 1.68 mmol, 7.00 eq.) as coupling reagents. Afterwards, Dde-removal was performed according to GP5. The SiFA‑BA moiety (67.7 mg, 0.24 mmol, 1.00 eq.) was attached using TBTU (154 mg, 0.48 mmol, 2.00 eq.), HOAt (65.3 mg, 0.48 mmol, 2.00 eq.) and *sym*-collidine (223 µL, 1.68 mmol, 7.00 eq.) as coupling reagents and incubation for at least 2 h. Removal of the Fmoc protective group (GP3) was followed by coupling of the DOTA moiety. Therefore, DOTA-NHS (91.4 mg, 0.12 mmol, 0.50 eq.) and DIPEA (286 µL, 1.68 mmol, 7.00 eq.) were each dissolved in DMF. First, DIPEA in DMF was added to the resin for preactivation. After five minutes, DOTA-NHS in DMF was added and incubated with the resin‑bound amine for 70.5 h. An adequate conversion (RP-HPLC/MS analysis after GP6) was reached and the peptide was cleaved off the resin with TFA/TIPS/DCM (95/2.5/2.5, GP8) and purified afterwards by preparative RP‑HPLC (30 - 80% B in 20 min, Method B, 5 mL/min and 25 - 70% B in 15 min, Method B, 1 mL/min). Subsequent lyophilization afforded 3.17 mg (0.95% yield, chemical purity 95.5%) of pure product **8** as a colorless powder.

Chemical formula: C_62_H_101_FN_12_O_21_Si;

Molecular weight: 1397.64 g/mol;

Exact mass: 1396.70 g/mol;

t*_R_*-value: 11.8 min (10 - 90% B in 15 min, Method A)

Capacity factor *k:* 6.9

ESI-MS: calculated monoisotopic mass (C_62_H_101_FN_12_O_21_Si): 1396.70;

found: ESI (positive ion mode): *m/z*= 699.4 [M(**8**)+2H]^2+^, 1398.9 [M(**8**)+H]^+^;

*^nat^Lu-2-Aminoheptanoic acid derivative* ***8*** *(^nat^Lu‑****8****)*

100 µL of the precursor (**8**) (2.00 mM in DMSO, 0.20 µmol, 1.00 eq.) were added to 60.0 µL of LuCl_3_ (20 mM in Tracepur^®^-H_2_O, 1.20 µmol, 6.00 eq.) and 40.0 µL Tracepur^®^-H_2_O. The reaction mixture was heated for 30 min at 95 °C and afforded ^nat^Lu-**8** in 94.9% chemical purity (>99% yield), determined by RP‑HPLC (220 nm). This ^nat^Lu-**8** solution (now 1.00 mM) was directly used as stock solution for affinity determination.

Chemical formula: C_62_H_98_FLuN_12_O_21_Si;

Molecular weight: 1569.58 g/mol;

Exact mass: 1568.61 g/mol;

t*_R_*-value: 11.7 min (10 - 90% B in 15 min, Method A)

Capacity factor *k:* 6.8

ESI-MS: calculated monoisotopic mass (C_62_H_98_FLuN_12_O_21_Si): 1568.61;

found: ESI (positive ion mode): *m/z*= 784.5 [M(^nat^Lu-**8**)+2H]^2+^, 1568.0 [M(^nat^Lu‑**8**)+H]^+^;

- - - 1. Furyl derivative 9

*Tert-butyl (S)-2-amino-3-(furan-2-yl)propanoate (****24*TFA****)*

Compound **24** was synthesized in analogy to a previously published procedure by Hyun et al.^(8)^ with some minor modifications. 3-(2-Furyl)-L-alanine (**S-34**) (300 mg, 2.93 mmol, 1.00 eq.) was dissolved in 6.65 mL of tert‑butyl acetate (**S-32**) and 70% (v/v) perchloric acid (aq.) (359 µL, 4.42 mmol, 2.29 eq.) was added slowly. The reaction mixture was stirred for 22 h at room temperature. H_2_O and some drops of 0.3 M HCl were added for quenching. The pH value of the aqueous phase was adjusted to 9 with 10% (w/v) Na_2_CO_3_ (aq.) and extracted three times with DCM. The combined organic phases were dried over Na_2_SO_4_, filtered and the solvent was removed under reduced pressure. Purification by column chromatography (DCM/MeOH/acetone = 5/1/1) and preparative RP-HPLC (30 ‑ 75% B in 20 min, Method B, 9 mL/min) yielded 65.5 mg (10.4%) of pure product **24***TFA as a colorless solid.

Chemical formula (w/o TFA): C_11_H_17_NO_3_;

Molecular weight (w/o TFA): 211.26 g/mol;

Exact mass (w/o TFA): 211.12 g/mol;

t*_R_*-value: 6.93 min (10 - 90% B in 15 min, Method A)

Capacity factor *k:* 3.6

*R*_f_-value: 0.69 (DCM/MeOH/acetone = 5/1/1)

ESI-MS: calculated monoisotopic mass (C_11_H_17_NO_3_): 211.12;

found: ESI (positive ion mode): *m/z*= 212.1 [M(**24**)+H]^+^, 253.1 [M(**24**)+MeCN+H]^+^;

3-(2-Furyl)-L-alanine*-OtBu-(carbonyl)-* L*‑Glu[D-Orn(Dde)-2-CT]-OtBu (****S-35****)*

Compound **20** (for the detailed synthesis procedure: see section 3.1.2.1) (0.29 mmol, 1.45 eq.) was transferred into a round bottom flask and dissolved in 8.00 mL DCE. At 0 °C, triethylamine (69.8 µL, 0.50 mmol, 2.50 eq.) and **24***TFA (65.5 mg, 0.20 mmol, 1.00 eq.) were added and the mixture was stirred for further 5 min at 0 °C. Afterwards, it was warmed to 40 °C and stirred for 17 h under argon atmosphere. The resin was washed with DCM (4x), some resin beads were taken and treated with HFIP/DCM (1/4) according to GP7. RP‑HPLC/MS analysis revealed nearly complete conversion to product **S‑35**.

Chemical formula: C_36_H_54_N_4_O_11_;

Molecular weight: 718.85 g/mol;

Exact mass: 718.38 g/mol;

t*_R_*-value: 12.4 min (10 - 90% B in 15 min, Method A)

Capacity factor *k:* 7.3

ESI-MS: calculated monoisotopic mass (C_36_H_54_N_4_O_11_): 718.38;

found: ESI (positive ion mode): *m/z*= 718.8 [M(**S‑35**)+H]^+^, 740.7 [M(**S‑35**+Na]^+^,

756.8 [M(**S‑35**)+K]^+^;

*Furyl derivative* ***9***

Further reactions on resin-bound compound **S-35** (0.29 mmol, 1.00 eq.) were performed according to standard Fmoc‑SPPS on 2‑CT resin, applying the above-mentioned methods (GP2 - GP8). In brief, the Dde protective group was removed (GP4) and succinic anhydride (203 mg, 2.03 mmol, 7.00 eq.) was coupled (GP2) over a period of at least 2.5 h, only using DIPEA (345 µL, 2.03 mmol, 7.00 eq.) and no further coupling reagents. The peptide was elongated with Fmoc‑D‑Lys-O*t*Bu*HCl (267 mg, 0.58 mmol, 2.00 eq.). Therefore, the resin‑bound acid was preactivated for five minutes using TBTU (186 mg, 0.58 mmol, 2.00 eq.), HOAt (78.9 mg, 0.58 mmol, 2.00 eq.) and DIPEA (296 µL, 1.74 mmol, 6.00 eq.). The amino acid was dissolved in DMF, added to the preactivated resin and shaken for at least 2.5 h. Fmoc-removal was conducted (GP3) and Fmoc‑D-Dap(Dde)-OH (285 mg, 0.58 mmol, 2.00 eq.) was coupled for at least 2.5 h using TBTU (186 mg, 0.58 mmol, 2.00 eq.), HOAt (78.9 mg, 0.58 mmol, 2.00 eq.) and *sym*-collidine (269 µL, 2.03 mmol, 7.00 eq.) as coupling reagents. Afterwards, Dde-removal was performed according to GP5. The SiFA‑BA moiety (81.8 mg, 0.29 mmol, 1.00 eq.) was attached using TBTU (186 mg, 0.58 mmol, 2.00 eq.), HOAt (78.9 mg, 0.58 mmol, 2.00 eq.) and *sym*-collidine (269 µL, 2.03 mmol, 7.00 eq.) as coupling reagents and incubation for at least 2 h. Removal of the Fmoc protective group (GP3) was followed by coupling of the DOTA moiety. Therefore, DOTA-NHS (110 mg, 0.15 mmol, 0.50 eq.) and DIPEA (345 µL, 2.03 mmol, 7.00 eq.) were each dissolved in DMF. First, DIPEA in DMF was added to the resin for preactivation. After five minutes, DOTA-NHS in DMF was added and incubated with the resin‑bound amine for 13 h. An adequate conversion (RP-HPLC/MS analysis after GP6) was reached and the peptide was cleaved off the resin with TFA/TIPS/DCM (95/2.5/2.5, GP8) and purified afterwards by preparative RP‑HPLC (35 - 80% B in 20 min, Method B, 5 mL/min). Subsequent lyophilization afforded 8.72 mg (2.14%, yield, chemical purity >99%) of pure product **9** as a colorless powder.

Chemical formula: C_62_H_95_FN_12_O_22_Si;

Molecular weight: 1407.59 g/mol;

Exact mass: 1406.64 g/mol;

t*_R_*-value: 11.3 min (10 - 90% B in 15 min, Method A)

Capacity factor *k:* 6.5

ESI-MS: calculated monoisotopic mass (C_62_H_95_FN_12_O_22_Si): 1406.64;

found: ESI (positive ion mode): *m/z*= 703.8 [M(**9**)+2H]^2+^, 1406.6 [M(**9**)+H]^+^;

*^nat^Lu-Furyl derivative* ***9*** *(^nat^Lu‑****9****)*

100 µL of the precursor (**9**) (2.00 mM in DMSO, 0.20 µmol, 1.00 eq.) were added to 60.0 µL of LuCl_3_ (20 mM in Tracepur^®^-H_2_O, 1.20 µmol, 6.00 eq.) and 40.0 µL Tracepur^®^-H_2_O. The reaction mixture was heated for 30 min at 95 °C and afforded ^nat^Lu-**9** in 98.1% chemical purity (>99% yield), determined by RP‑HPLC (220 nm). This ^nat^Lu-**9** solution (now 1.00 mM) was directly used as stock solution for affinity determination.

Chemical formula: C_62_H_92_FLuN_12_O_22_Si;

Molecular weight: 1579.53 g/mol;

Exact mass: 1578.56 g/mol;

t*_R_*-value: 11.2 min (10 - 90% B in 15 min, Method A)

Capacity factor *k:* 6.5

ESI-MS: calculated monoisotopic mass (C_62_H_92_FLuN_12_O_22_Si): 1578.56;

found: ESI (positive ion mode): *m/z*= 789.7 [M(^nat^Lu-**9**)+2H]^2+^, 1579.3 [M(^nat^Lu‑**9**)+H]^+^;

- - - 1. Alkyne derivative 10

(2S)-2-Amino-4-pentynoate-*OtBu-(carbonyl)-L‑Glu[D-Orn(Dde)-2-CT]-OtBu (****S-36****)*

Compound **20** (for the detailed synthesis procedure: see section 3.1.2.1) (0.24 mmol, 1.00 eq.) was transferred into a round bottom flask and dissolved in 2.18 mL DCE. At 0 °C, triethylamine (83.2 µL, 0.60 mmol, 2.50 eq.) and *tert*-butyl (2*S*)-2-amino-4-pentynoate (**25**)*HCl  (59.6 mg, 0.29 mmol, 1.20 eq.) were added and the mixture was stirred for further 5 min at 0 °C. Afterwards, it was warmed to 40 °C and stirred for 20 h under argon atmosphere. The resin was washed with DCM (4x), some resin beads were taken and treated with HFIP/DCM (1/4) according to GP7. RP‑HPLC/MS analysis revealed nearly complete conversion to product **S-36**.

Chemical formula: C_34_H_52_N_4_O_10_;

Molecular weight: 676.81 g/mol;

Exact mass: 676.37 g/mol;

t*_R_*-value: 6.05 min (40 - 95% B in 15 min, Method A)

Capacity factor *k:* 3.0

ESI-MS: calculated monoisotopic mass (C_34_H_52_N_4_O_10_): 676.37;

found: ESI (positive ion mode): *m/z*= 677.0 [M(**S-36**)+H]^+^, 699.0 [M(**S-36**)+Na]^+^;

*Alkyne derivative* ***10***

Further reactions on resin-bound compound **S-36** (0.24 mmol, 1.00 eq.) were performed according to standard Fmoc‑SPPS on 2‑CT resin, applying the above-mentioned methods (GP2 - GP8). In brief, the Dde protective group was removed (GP4) and succinic anhydride (170 mg, 1.68 mmol, 7.00 eq.) was coupled (GP2) over a period of at least 2.5 h, only using DIPEA (286 µL, 1.68 mmol, 7.00 eq.) and no further coupling reagents. The peptide was elongated with Fmoc‑D‑Lys-O*t*Bu*HCl (221 mg, 0.48 mmol, 2.00 eq.). Therefore, the resin‑bound acid was preactivated for five minutes with TBTU (154 mg, 0.48 mmol, 2.00 eq.), HOAt (65.3 mg, 0.48 mmol, 2.00 eq.) and DIPEA (245 µL, 1.44 mmol, 6.00 eq.). The amino acid was dissolved in DMF, added to the preactivated resin and shaken for at least 2.5 h. Fmoc-removal was conducted (GP3) and Fmoc‑D-Dap(Dde)-OH (235 mg, 0.48 mmol, 2.00 eq.) was coupled for at least 2.5 h using TBTU (154 mg, 0.48 mmol, 2.00 eq.), HOAt (65.3 mg, 0.48 mmol, 2.00 eq.) and *sym*-collidine (223 µL, 1.68 mmol, 7.00 eq.) as coupling reagents. Afterwards, Dde-removal was performed according to GP5. The SiFA‑BA moiety (67.7 mg, 0.24 mmol, 1.00 eq.) was attached using TBTU (154 mg, 0.48 mmol, 2.00 eq.), HOAt (65.3 mg, 0.48 mmol, 2.00 eq.) and *sym*-collidine (223 µL, 1.68 mmol, 7.00 eq.) as coupling reagents and incubation for at least 2 h. Removal of the Fmoc protective group (GP3) was followed by coupling of the DOTA moiety. Therefore, DOTA-NHS (91.4 mg, 0.12 mmol, 0.50 eq.) and DIPEA (286 µL, 1.68 mmol, 7.00 eq.) were each dissolved in DMF. First, DIPEA in DMF was added to the resin for preactivation. After five minutes, DOTA-NHS in DMF was added and incubated with the resin‑bound amine for 71 h. An adequate conversion (RP-HPLC/MS analysis after GP6) was reached and the peptide was cleaved off the resin with TFA/TIPS/DCM (95/2.5/2.5, GP8) and purified afterwards by preparative RP‑HPLC (30 - 80% B in 20 min, Method B, 5 mL/min). Subsequent lyophilization afforded 10.9 mg (3.33% yield, chemical purity 98.5%) of pure product **10** as a colorless powder.

Chemical formula: C_60_H_93_FN_12_O_21_Si;

Molecular weight: 1365.55 g/mol;

Exact mass: 1364.63 g/mol;

t*_R_*-value: 11.1 min (10 - 90% B in 15 min, Method A)

Capacity factor *k:* 6.4

ESI-MS: calculated monoisotopic mass (C_60_H_93_FN_12_O_21_Si): 1364.63;

found: ESI (positive ion mode): *m/z*= 682.7 [M(**10**)+2H]^2+^, 1364.4 [M(**10**)+H]^+^;

*^nat^Lu-Alkyne derivative* ***10*** *(^nat^Lu‑****10****)*

100 µL of the precursor (**10**) (2.00 mM in DMSO, 0.20 µmol, 1.00 eq.) were added to 60.0 µL of LuCl_3_ (20 mM in Tracepur^®^-H_2_O, 1.20 µmol, 6.00 eq.) and 40.0 µL Tracepur^®^-H_2_O. The reaction mixture was heated for 30 min at 95 °C and afforded ^nat^Lu-**10** in 99.1% chemical purity (>99% yield), determined by RP‑HPLC (220 nm). This ^nat^Lu-**10** solution (now 1.00 mM) was directly used as stock solution for affinity determination.

Chemical formula: C_60_H_90_FLuN_12_O_21_Si;

Molecular weight: 1537.49 g/mol;

Exact mass: 1536.55 g/mol;

t*_R_*-value: 10.9 min (10 - 90% B in 15 min, Method A)

Capacity factor *k:* 6.3

ESI-MS: calculated monoisotopic mass (C_60_H_90_FLuN_12_O_21_Si): 1536.55;

found: ESI (positive ion mode): *m/z*= 769.6 [M(^nat^Lu-**10**)+2H]^2+^, 1538.4 [M(^nat^Lu‑**10**)+H]^+^;

*[^177^Lu]Lu-Alkyne derivative* ***10*** *([^177^Lu]Lu-****10****)*

5.00 µL of the precursor (**10**) (0.20 mM in DMSO, 1.00 nmol, 1.00 eq.) were added to 10.0 µL of 1 M NaOAc buffer (aq.) (pH = 5.5). Subsequently, 37.5 to 62.0 MBq [^177^Lu]LuCl_3_ (A_s_ > 3000 GBq/mg, 740 MBq/mL, 0.04 M HCl, ITG, Garching, Germany) were added and the mixture was filled up to 100 µL with 0.04 M HCl (in Tracepur^®^-H_2_O). 10.0 µL of 0.1 M sodium ascorbate (aq.) (in Tracepur^®^-H_2_O) were added and the reaction mixture was heated for 25 min at 95 °C. Occasionally, removal of free [^177^Lu]Lu^3+^ via HLB cartridge (30 mg) was required, which afforded [^177^Lu]Lu-**10** in 70.3 or >99% (n = 3) isolated RCY (d.c. to the start of synthesis). The apparent A_m_ were 26.3 to 62.0 GBq/µmol at the end of synthesis. RCP as determined by radio‑RP‑HPLC and radio‑TLC was 93.6 ± 2.5%.

Chemical formula: C_60_H_90_F^177^LuN_12_O_21_Si;

Molecular weight: 1539.47 g/mol;

Exact mass: 1538.55 g/mol;

t*_R_*-value: 10.3 min (10 - 90% B in 15 min, Method A)

t*_R_*-value of co-injected cold standard: 10.2 min (10 - 90% B in 15 min, Method A)

Capacity factor *k*: 5.9

- - - 1. Tetrazole derivative 11

*Tert-butyl N^5^-benzyl-N^2^-((benzyloxy)carbonyl)-L-glutaminate* *(****S-39****)*

Compound **S-39** was synthesized in analogy to a previously published procedure by Kozikowski et al.^(9)^ Benzyl amine (**S-38**) (305 μL, 2.80 mmol, 1.40 eq.) and BOP (1.06 g, 2.40 mmol, 1.20 eq.) were added to a solution of *N*-Cbz-L‑Glu-O*t*Bu (**S‑37**) (675 mg, 2.00 mmol, 1.00 eq.) in 18.0 mL DMF. The resulting solution was cooled to 0 °C and triethylamine (638 μL, 4.60 mmol, 2.30 eq.) was added. After stirring for 66 h at room temperature the reaction mixture was poured into ice‑cold water (100 mL) and extracted four times with EtOAc. The organic layer was washed successively with 1 M HCl, H_2_O, saturated NaHCO_3_ and brine. The organic phase was dried over Na_2_SO_4,_ filtered and the solvent was removed *in vacuo*. Purification by column chromatography (EtOAc/*n*-hexane = 1/1) yielded 876 mg (2.05 mmol, >99%) of *N*‑Cbz‑L‑Gln(Bn)‑O*t*Bu (**S-39**) as a colorless solid.

Chemical formula: C_24_H_30_N_2_O_5_;

Molecular weight: 426.51 g/mol;

Exact mass: 426.22 g/mol;

t*_R_*-value: n.d.

Capacity factor *k:* n.d.

R*_f_*-value: 0.54 (EtOAc/*n*-hexane = 1/1)

ESI-MS: n.d.

^1^H-NMR (400 MHz, CDCl_3_) δ(ppm): 7.38 – 7.26 (m, 10H, H(1, 1’, 2, 2’, 3, 4, 4’, 5, 6, 6’), 6.27 (s, 1H, H(7)), 5.56 (d, ^3^*J* = 8.1 Hz, 1H, H(8)), 5.08 (s, 2H, H(9)), 4.42 (*virt*. t, 2H, H(10)), 4.23 (td, ^3^*J*= 8.8, 3.8 Hz, 1H, H(11)), 2.26 (*virt*. ddt, 3H, H(12, 13)), 2.02 – 1.86 (m, 1H, H(14)), 1.45 (s, 9H, H(15, 15’, 15’’)).

^13^C‑NMR (101 MHz, CDCl_3_) δ(ppm): 171.92 (s, 1C, C(1)), 171.15 (s, 1C, C(2)), 156.51 (s, 1C, C(3)), 138.37 (s, 1C, C(4)), 136.37 (s, 1C, C(5)), 128.82 (s, 2C, C(6, 6’)), 128.65 (s, 2C, C(7, 7’)), 128.33 (s, 1C, C(8)), 128.26 (s, 2C, C(9, 9’)), 127.95 (s, 2C, C(10, 10’)), 127.61 (s, 1C, C(11)), 82.68 (s, 1C, C(12)), 67.16 (s, 1C, C(13)), 54.13 (s, 1C, C(14)), 43.84 (s, 1C, C(15)), 32.72 (s, 1C, C(16)), 29.42 (s, 1C, C(17)),

28.11 (s, 3C, C(18, 18’, 18’’)).

*Tert-butyl (S)-4-(1-benzyl-1H-tetrazol-5-yl)-2-(((benzyloxy)carbonyl)amino)butanoate (****S-40****)*

Compound **S-40**was synthesized in analogy to a previously published procedure by Kozikowski et al.^(9)^ Diisopropyl azodicarboxylate (530 μL, 2.70 mmol, 1.35 eq.) was added to a solution of PPh_3_ (683 mg, 2.60 mmol, 1.30 eq.) and *N‑*Cbz‑L‑Gln(Bn)‑O*t*Bu (**S-39**) (853 mg, 2.00 mmol, 1.00 eq.) in 10 mL ice‑cold, anhydrous MeCN over two minutes. Trimethylsilyl azide (372 μL, 2.80 mmol, 1.40 eq.) was added over five minutes, and the solution was allowed to stir for 15 h at room temperature. Afterwards, the solution was cooled to 0 °C and 726 μL of a 3 M NaNO_2_ solution (aq.) (152 mg, 2.20 mmol, 1.10 eq.) was added. The mixture was allowed to stir for 30 min at room temperature and 3.30 mL of cerium(IV) ammonium nitrate (1.21 g, 2.20 mmol, 1.10 eq.) in H_2_O was added. The solution was stirred for further 20 min and subsequently poured into ice‑cold H_2_O. The mixture was extracted three times with DCM and the combined organic phases were washed once with water, then dried over Na_2_SO_4,_ filtered and the solvent removed *in vacuo*. Purification by column chromatography (EtOAc/*n*-hexane = 1/2) yielded 222 mg (24.6%) of product **S-40** as a colorless solid.

Chemical formula: C_24_H_29_N_5_O_4_;

Molecular weight: 451.53 g/mol;

Exact mass: 451.22 g/mol;

t*_R_*-value: 14.5 min (10 - 90% B in 15 min, Method A)

Capacity factor *k:* 8.7

R*_f_*-value: 0.28 (EtOAc/*n*-hexane = 1/2)

ESI-MS: calculated monoisotopic mass (C_9_H_17_N_5_O_2_): 451.22;

found: ESI (positive ion mode): *m/z*= 395.7 [M(**S-40**)-tBu+H]^+^, 451.7 [M(**S-40**)+H]^+^;

^1^H-NMR (400 MHz, CDCl_3_) δ(ppm): 7.43 – 7.28 (m, 8H, H(1, 1’, 2, 2’, 3, 3’, 4, 4’)), 7.14 (*virt*. dd, 2H, H(5, 6)), 5.56 – 5.30 (m, 2H, H(7)), 5.24 – 4.99 (m, 2H, H(8)), 4.26 (td, ^3^*J*= 8.2, 4.4 Hz, 1H, H(9)), 2.75 *(virt*. tq, 2H, H(10)), 2.34 (*virt*. dq, 1H, H(11)), 2.01 (*virt*. dddd, 1H, H(12)), 1.42 (s, 9H, H(13, 13’, 13’’)).

^13^C‑NMR (101 MHz, CDCl_3_) δ(ppm): 170.51 (s, 1C, C(1)), 156.19 (s, 1C, C(2)), 154.26 (s, 1C, C(3)), 136.31 (s, 1C, C(4)), 133.33 (s, 1C, C(5)), 129.40 (s, 2C, C(6, 6’)), 129.11 (s, 1C, C(7)), 128.73 (s, 2C, C(8, 8’)), 128.45 (s, 1C, C(9)), 128.30 (s, 2C, C(10, 10’)), 127.67 (s, 2C, C(11, 11’)), 83.11 (s, 1C, C(12)), 67.23 (s, 1C, C(13)), 53.93 (s, 1C, C(14)), 50.90 (s, 1C, C(15)), 30.29 (s, 1C, C(16)), 28.06 (s, 3C, C(17, 17’, 17’’)), 19.98 (s, 1C, C(18)).

*Tert-butyl (S)-2-amino-4-(1H-tetrazol-5-yl)butanoate (****S-41****)*

Compound **S-40** (120 mg, 0.27 mmol, 1.00 eq.) was dissolved in 15.0 mL MeOH and the solution was flushed 5 minutes with argon to remove dissolved oxygen. 12.0 mg of palladium on carbon (10% wt) (corresponds to 1.20 mg palladium, 11.3 µmol, 0.04 eq.) were added and the flask was sealed with a rubber septum. Remaining air was displaced with argon and subsequently replaced by hydrogen gas. The mixture was stirred under hydrogen atmosphere at room temperature for 23 h. Palladium on carbon was filtered off, and the solvent was evaporated *in vacuo*. This afforded a mixture of product **S‑41** (minor portion) and reactant **S-40** with free α-amine, still bearing the Bn-protective group at the tetrazole moiety (major portion). However, as a certain conversion to product **S‑41** could be observed by RP‑HPLC/MS analysis and the *N*‑Cbz‑protective group was cleaved off efficiently, the crude product was used in the next step without further purification.

Chemical formula: C_9_H_17_N_5_O_2_;

Molecular weight: 227.27 g/mol;

Exact mass: 227.14 g/mol;

t*_R_*-value: 4.6 min (10 - 90% B in 15 min, Method A)

Capacity factor *k:* 2.1

ESI-MS: calculated monoisotopic mass (C_9_H_17_N_5_O_2_): 227.14;

found: ESI (positive ion mode): *m/z*= 228.0 [M(**S‑41**)+H]^+^, 268.7 [M(**S‑41**)+MeCN+H]^+^;

5-Benzyl 1-(tert-butyl) (((S)-1-(tert-butoxy)-1-oxo-4-(1H-tetrazol-5-yl)butan-2-yl)carbamoyl)-L-glutamate (**S-42**)

Compound **S-42** was synthesized in analogy to a previously published procedure by Weineisen et al.^(6)^ with some minor modifications. *Tert*-butyl (*S*)-2-amino-4-(1*H*-tetrazol-5-yl)butanoate (**S‑41**) (~100 mg crude product, ~266 µmol, 1.20 eq.) was dissolved in 2.00 mL DCE and compound **S-28** (86.0 mg, 222 µmol, 1.00 eq.) was added (for the detailed synthesis procedure of **S-28**: see section 3.1.2.3). At 0 °C, triethylamine (76.9 µL, 555 µmol, 2.50 eq.) was added and the mixture was stirred for further five minutes at 0 °C. The solution was warmed to 40 °C and stirred for 20 h under argon atmosphere. The solvent was removed *in vacuo* and the crude product was purified via flash chromatography (10 ‑ 90% B in 15 min, Method D, 12 mL/min), which afforded 39.6 mg (32.7%) of compound **S-42** as a colorless solid.

Chemical formula: C_26_H_38_N_6_O_7_;

Molecular weight: 546.63 g/mol;

Exact mass: 546.28 g/mol;

t*_R_*-value: 11.3 min (30 - 90% B in 15 min, Method A)

Capacity factor *k:* 6.5

ESI-MS: calculated monoisotopic mass (C_26_H_38_N_6_O_7_): 546.28;

found: ESI (positive ion mode): *m/z*= 490.4 [M(**S-42**)-*t*Bu+H]^+^, 546.7 [M(**S‑42**)+H]^+^,

568.8 [M(**S-42**)+Na]^+^;

*(S)-5-(tert-butoxy)-4-(3-((S)-1-(tert-butoxy)-1-oxo-4-(1H-tetrazol-5-yl)butan-2-yl)ureido)-5-oxopentanoic acid (****26****)*

Compound **S‑42** (17.0 mg, 31.2 µmol, 1.00 eq.) was dissolved in 6.00 mL MeOH and the solution was flushed 5 minutes with argon to remove dissolved oxygen. 3.30 mg of palladium on carbon (10% wt) (corresponds to 330 µg palladium, 3.12 µmol, 0.10 eq.) were added and the flask was sealed with a rubber septum. Remaining air was displaced with argon and subsequently replaced by hydrogen gas. The mixture was stirred under hydrogen atmosphere at room temperature for 69.5 h. Palladium on carbon was filtered off, and the solvent was evaporated *in vacuo*. This afforded 44.0 mg of crude product as a colorless oil, with some minor by‑products and remaining reactant **S-42**. As the main component could be identified as product **26** by RP‑HPLC/MS analysis, the crude product was used in the next step without further purification.

Chemical formula: C_19_H_32_N_6_O_7_;

Molecular weight: 456.50 g/mol;

Exact mass: 456.23 g/mol;

t*_R_*-value: 8.94 min (10 - 90% B in 15 min, Method A)

Capacity factor *k:* 5.0

ESI-MS: calculated monoisotopic mass (C_19_H_32_N_6_O_7_): 456.23;

found: ESI (positive ion mode): *m/z*= 345.9 [M(**26**)-2*t*Bu+H]^+^, 456.7 [M(**26**)+H]^+^,

478.7 [M(**26**)+Na]^+^, 912.5 [M_2_(**26**)+H]^+^, 934.7 [M_2_(**26**)+Na]^+^, 950.3 [M_2_(**26**)+K]^+^;

(S)-2-amino-4-(1H-tetrazol-5-yl)butanoic acid-*OtBu-(carbonyl)-L-Glu[D-Orn(Dde)-2-CT]-OtBu (****S-43****)*

According to GP2, fragment **26** (28.5 mg, ~62.4 µmol, 1.00 eq.) was coupled to resin‑bound H‑D‑Orn(Dde) (**18**) (89.0 µmol 1.43 eq.) with TBTU (40.1 mg, 125 µmol, 2.00 eq.) and HOAt (17.0 mg, 125 µmol, 2.00 eq.) as coupling reagents and *sym*-collidine (74.5 µL, 562 µmol, 9.00 eq.) as base. After shaking for 14 h at room temperature, formation of product **S-43** could be confirmed (GP7), as only one major peak with the expected m/z-ratio of 734.9 occurred.

Chemical formula: C_34_H_54_N_8_O_10_;

Molecular weight: 734.85 g/mol;

Exact mass: 734.40 g/mol;

t*_R_*-value: 9.91 min (10 - 90% B in 15 min, Method A)

Capacity factor *k:* 5.6

ESI-MS: calculated monoisotopic mass (C_34_H_54_N_8_O_10_): 734.40;

found: ESI (positive ion mode): *m/z*= 734.9 [M(**S-43**)+H]^+^;

*Tetrazole derivative* ***11***

Further reactions on resin-bound compound **S-43** (89.0 µmol, 1.00 eq.) were performed according to standard Fmoc‑SPPS on 2‑CT resin, applying the above-mentioned methods (GP2 - GP8). In brief, the Dde protective group was removed (GP4) and succinic anhydride (62.3 mg, 0.62 mmol, 7.00 eq.) was coupled (GP2) over a period of at least 2.5 h, only using DIPEA (106 µL, 0.62 mmol, 7.00 eq.) and no further coupling reagents. The peptide was elongated with Fmoc‑D‑Lys-O*t*Bu*HCl (82.1 mg, 0.18 mmol, 2.00 eq.). Therefore, the resin‑bound acid was preactivated for five minutes with TBTU (57.2 mg, 0.18 mmol, 2.00 eq.), HOAt (24.2 mg, 0.18 mmol, 2.00 eq.) and DIPEA (91.0 µL, 0.53 mmol, 6.00 eq.). The amino acid was dissolved in DMF, added to the preactivated resin and shaken for at least 2.5 h. Fmoc-removal was conducted (GP3) and Fmoc‑D-Dap(Dde)-OH (88.3 mg, 0.18 mmol, 2.00 eq.) was coupled for at least 2.5 h using TBTU (57.2 mg, 0.18 mmol, 2.00 eq.), HOAt (24.2 mg, 0.18 mmol, 2.00 eq.) and *sym*-collidine (83.5 µL, 0.62 mmol, 7.00 eq.) as coupling reagents. Afterwards, Dde-removal was performed according to GP5. The SiFA‑BA moiety (25.1 mg, 89.0 µmol, 1.00 eq.) was attached using TBTU (57.2 mg, 0.18 mmol, 2.00 eq.), HOAt (24.2 mg, 0.18 mmol, 2.00 eq.) and *sym*-collidine (83.5 µL, 0.62 mmol, 7.00 eq.) as coupling reagents and incubation for at least 2 h. Removal of the Fmoc protective group (GP3) was followed by coupling of the DOTA moiety. Therefore, DOTA-NHS (33.9 mg, 44.5 µmol, 0.50 eq.) and DIPEA (106 µL, 0.62 mmol, 7.00 eq.) were each dissolved in DMF. First, DIPEA in DMF was added to the resin for preactivation. After five minutes, DOTA-NHS in DMF was added and incubated with the resin‑bound amine for 15 h. As only low conversion (RP-HPLC/MS analysis after GP7) was reached, further DOTA-NHS (33.9 mg, 44.5 µmol, 0.50 eq.) and DIPEA (106 µL, 0.62 mmol, 7.00 eq.) was added and again incubated for 71 h. Again, no increased conversion could be observed and hence, HOAt (24.2 mg, 0.18 mmol, 2.00 eq.) and DIPEA (212 µL, 1.24 mmol, 14.0 eq.) were added to the reaction mixture. RP‑HPLC/MS analysis after 20 h revealed no further significant increase of product signal and hence, the peptide was cleaved off the resin with TFA/TIPS/DCM (95/2.5/2.5, GP8) and purified afterwards by preparative RP‑HPLC (25 - 80% B in 20 min, Method B, 5 mL/min and 25 ‑ 70% B in 15 min, Method B, 1 mL/min). Subsequent lyophilization afforded 3.55 mg (2.80% yield, chemical purity >99%) of pure product **11** as a colorless powder.

Chemical formula: C_60_H_95_FN_16_O_21_Si;

Molecular weight: 1423.59 g/mol;

Exact mass: 1422.66 g/mol;

t*_R_*-value: 10.8 min (10 - 90% B in 15 min, Method A)

Capacity factor *k:* 6.2

ESI-MS: calculated monoisotopic mass (C_60_H_95_FN_16_O_21_Si): 1422.66;

found: ESI (positive ion mode): *m/z*= 711.6 [M(**11**)+2H]^2+^, 1422.5 [M(**11**)+H]^+^;

*^nat^Lu-Tetrazole derivative* ***11*** *(^nat^Lu‑****11****)*

100 µL of the precursor (**11**) (2.00 mM in DMSO, 0.20 µmol, 1.00 eq.) were added to 60.0 µL of LuCl_3_ (20 mM in Tracepur^®^-H_2_O, 1.20 µmol, 6.00 eq.) and 40.0 µL Tracepur^®^-H_2_O. The reaction mixture was heated for 30 min at 95 °C and afforded ^nat^Lu-**11** in 92.9% chemical purity (>99% yield), determined by RP‑HPLC (220 nm). This ^nat^Lu-**11** solution (now 1.00 mM) was directly used as stock solution for affinity determination.

Chemical formula: C_60_H_92_FLuN_16_O_21_Si;

Molecular weight: 1595.54 g/mol;

Exact mass: 1594.58 g/mol;

t*_R_*-value: 10.6 min (10 - 90% B in 15 min, Method A)

Capacity factor *k:* 6.1

ESI-MS: calculated monoisotopic mass (C_60_H_92_FLuN_16_O_21_Si): 1594.58;

found: ESI (positive ion mode): *m/z*= 797.5 [M(^nat^Lu-**11**)+2H]^2+^, 816.6 [M(^nat^Lu‑**11**)+K+H]^2+^,

1595.0 [M(^nat^Lu‑**11**)+H]^+^;

*[^177^Lu]Lu-Tetrazole derivative* ***11*** *([^177^Lu]Lu-****11****)*

5.00 µL of the precursor (**11**) (0.20 mM in DMSO, 1.00 nmol, 1.00 eq.) were added to 10.0 µL of 1 M NaOAc buffer (aq.) (pH = 5.5). Subsequently, 42.2 to 63.5 MBq [^177^Lu]LuCl_3_ (A_s_ > 3000 GBq/mg, 740 MBq/mL, 0.04 M HCl, ITG, Garching, Germany) were added and the mixture was filled up to 100 µL with 0.04 M HCl (in Tracepur^®^-H_2_O). 10.0 µL of 0.1 M sodium ascorbate (aq.) (in Tracepur^®^-H_2_O) were added and the reaction mixture was heated for 25 min at 95 °C. This afforded [^177^Lu]Lu-**11** in >99% (n = 4) isolated RCY (d.c. to the start of synthesis). The apparent A_m_ ranged from 42.2 to 63.5 GBq/µmol at the end of synthesis. RCP as determined by radio‑RP‑HPLC and radio‑TLC was 97.2 ± 1.2%.

Chemical formula: C_60_H_92_F^177^LuN_16_O_21_Si;

Molecular weight: 1597.51 g/mol;

Exact mass: 1596.58 g/mol;

t*_R_*-value: 10.0 min (10 - 90% B in 15 min, Method A)

t*_R_*-value of co-injected cold standard: 9.9 min (10 - 90% B in 15 min, Method A)

Capacity factor *k*: 5.7

- - 1. Summary of cold complexation and radiolabeling reaction conditions and results

***Table 1*:** Reaction conditions, chemical purity and yield of the investigated ^nat^Ga‑ and ^nat^Lu‑PSMA ligands.

| PSMA inhibitor | Reaction conditions | Chemical purity | Yield^a^ |
| --- | --- | --- | --- |
| ^nat^Ga-2 | 75 °C, 30 min | 93.0% | 3.35% |
| ^nat^Ga-3 | 75 °C. 30 min | 97.2% | 26.5% |
| ^nat^Lu-3 | 70 °C, 25 min | 92.3% | >99% |
| ^nat^Ga-4 | 75 °C, 30 min | 97.6% | 15.7% |
| ^nat^Lu-5 | 70 °C, 30 min | >99% | >99% |
| ^nat^Lu-6 | 95 °C, 25 min | >99% | >99% |
| ^nat^Lu-7 | 95 °C, 25 min | >99% | >99% |
| ^nat^Lu-8 | 95 °C, 25 min | 94.9% | >99% |
| ^nat^Lu-9 | 95 °C, 25 min | 98.1% | >99% |
| ^nat^Lu-10 | 95 °C, 25 min | 99.1% | >99% |
| ^nat^Lu-11 | 95 °C, 25 min | 92.9% | >99% |

^a^calculated relative to the uncomplexed precursor. Number of experiments is n = 1.

***Table 2*:** Reaction conditions, RCY, apparent A_m_, used activities and RCP of the investigated [^177^Lu]Lu-PSMA ligands.

| PSMA inhibitor | Reaction conditions | RCY^a^ | apparent A_m_ [GBq/µmol] | used activities [MBq] | RCP | No. of experi-ments |
| --- | --- | --- | --- | --- | --- | --- |
| [^177^Lu]Lu-3 | 70 °C, 25 min | 69.7 ± 18.2% | 12.5 ‑ 21.9 | 22.3 ‑ 62.2 | 96.9 ± 2.1% | 6 |
| [^177^Lu]Lu-5 | 80 °C, 25 min | 44.0 ± 7.6% | 4.64 ‑ 14.5 | 14.0 ‑ 35.9 | 89.3 ± 1.9% | 4 |
| [^177^Lu]Lu-6 | 95 °C, 25 min | 81.4 ± 6.9% | 24.1 & 24.8 | 28.0 & 32.5 | 98.2 ± 0.1% | 2 |
| [^177^Lu]Lu-7 | 95 °C, 25 min | >99% | 27.4 | 27.4 | 94.7% | 1 |
| [^177^Lu]Lu-10 | 95 °C, 25 min | 70.3 to >99% | 26.3 ‑ 62.0 | 37.5 ‑ 62.0 | 93.6 ± 2.5% | 3 |
| [^177^Lu]Lu-11 | 95 °C, 25 min | >99% | 42.2 ‑ 63.5 | 42.2 - 63.5 | 97.2 ± 1.2% | 4 |

^a^decay corrected to the start of synthesis. Data for isolated RCY and RCP are expressed as mean ± SD.

- 1. *In vitro* experiments
     1. Cell culture

PSMA-positive LNCaP cells (300265; Cell Lines Service, Eppelheim, Germany) were cultivated in Dulbecco’s modified Eagle medium/Nutrition Mixture F-12 with GlutaMAX (1/1, DMEM‑F12, Thermo Fisher Scientific, Darmstadt, Germany) supplemented with 10% fetal bovine serum (Merck KGaA, Darmstadt, Germany) and kept at 37 °C in a humidified 5% CO_2_ atmosphere. One day (24 ± 2 h) prior to all *in vitro* experiments, the cultivated LNCaP cells were harvested using a mixture of trypsin/ethylenediaminetetraacetic acid (0.05%/0.02%) in phosphate-buffered saline (PBS) (Thermo Fisher Scientific, Darmstadt, Germany) and centrifuged at 1,300 rpm (ca. 190 × g) for 3 min at room temperature (Heraeus Megafuge 16, Thermo Fisher Scientific, Darmstadt, Germany). After centrifugation, the supernatant was disposed and the cell pellet was resuspended in culture medium. Cells were counted with a Neubauer hemocytometer (Paul Marienfeld GmbH & Co. KG, Lauda‑Königshofen, Germany) and seeded in 24-well plates (Greiner Bio‑One, Kremsmünster, Austria). IC_50_ values were determined by transferring 1.50 × 10^5^ cells/mL per well into 24‑well plates, whereas internalization was assessed by transferring 1.25 × 10^5^ cells/mL per well into Poly‑L-lysine (PLL)-coated 24‑well plates (Greiner Bio‑One, Kremsmünster, Austria).

- - 1. Affinity determinations (IC_50_)

The culture medium was removed and the cells were washed with 500 µL of Hank’s balanced salt solution (HBSS) (Merck KGaA, Darmstadt, Germany), containing 1% bovine serum albumin (BSA) (Merck KGaA, Darmstadt, Germany). Afterwards, 200 µL of HBSS (1% BSA) were added to each well and equilibrated on ice (4 °C) for 15 min. 25 µL/well of either HBSS (1% BSA) (= control) or of solutions, containing the respective unlabeled ligand in increasing concentrations (10^-10^- 10^-4^ M in HBSS) were added, followed by the addition of 25 µL of ([^125^I]I‑BA)KuE in HBSS (1%BSA) to each well. Experiments were carried out in triplicates for each concentration. The final concentrations of unlabeled ligand ranged from 10^-11^- 10^-5^ M and the final radioligand concentration was 0.2 nM in all binding assays. The cells were incubated for one hour at 4 °C. Incubation was terminated by removal of the incubation medium. The cells were washed with 250 µL of HBSS (1% BSA) and the wash medium was combined with the respective supernatant. This fraction represents the amount of free radioligand. The cells were lysed by addition of 250 µL of 1 M aqueous NaOH. The lysate of each well was transferred to the respective vial as well as 250 µL of 1 M NaOH used for rinsing the well. Quantification of the amount of free and bound activity was performed in a γ-counter. The corresponding IC_50_ values were calculated using the GraphPad PRISM7 software.

- - 1. **Internalization studies**

The culture medium was removed and the cells were washed with 500 µL of DMEM‑F12 containing 5% BSA. Afterwards, 200 µL of DMEM‑F12 (5% BSA) were added to each well and left to equilibrate at 37 °C for 15 min. 25 µL of DMEM‑F12 (5% BSA) were added to each well, followed by the addition of 25 µL of the respective ^177^Lu-labeled ligand (10.0 nM in DMEM‑F12 (5% BSA)). For blocking PSMA‑specific binding and uptake, 25 µL of 2‑(phosphonomethyl)pentane-1,5-dioic acid (2‑PMPA) (100 µM in DMEM (5% BSA)) instead of DMEM‑F12 (5% BSA) were added prior to radiotracer addition. The same procedure was conducted with ([^125^I]I‑BA)KuE (2.0 nM in DMEM‑F12 (5% BSA)) which served as the reference. Each experiment (control and blockade) was performed in triplicate. The final concentration of the ^177^Lu-labeled ligand was 1.0 nM and that of ([^125^I]I‑BA)KuE was 0.2 nM in all internalization assays. The cells were incubated for one hour at 37 °C. Incubation was terminated by placing the plate on ice (4 °C, 1 min) and removal of the incubation medium. The cells were washed with 250 µL of ice-cold PBS and the wash medium was combined with the respective supernatant. This fraction represents the amount of free radioligand. 250 µL of ice-cold 2‑PMPA (10 µM in PBS) were added and the cells were incubated for 10 min at 4 °C. Afterwards, the cells were rinsed again with 250 µL of ice-cold PBS and the wash medium was combined with the respective supernatant. This fraction represents the amount of cell surface-bound ligand. In the last step, the cells were lysed by addition of 250 µL of 1 M aqueous NaOH. After 20 min, the lysate of each well was transferred to the respective vial as well as 250 µL of 1 M NaOH used for rinsing the well. This fraction represents the amount of internalized radioligand. Quantification of the amount of free, cell surface-bound and internalized activity was performed in a γ‑counter. The corresponding internalization values were corrected for non-specific binding and normalized to the specific binding observed for the reference ([^125^I]I-BA)KuE.

- 1. *In vivo* experiments

All animal experiments were conducted in accordance with general animal welfare regulations in Germany (German animal protection act, as amended on 18.05.2018, Art. 141 G v. 29.3.2017 I 626, approval no. 55.2‑1-54-2532-71-13) and the institutional guidelines for the care and use of animals. To establish tumor xenografts, LNCaP cells (approx. 10^7^ cells) were suspended in 200 μL of a 1/1 mixture (v/v) of DMEM F‑12 and Matrigel (BD Biosciences, Heidelberg, Germany) and inoculated subcutaneously onto the right shoulder of 6 ‑ 8 weeks old CB17-SCID mice (Charles River Laboratories, Sulzfeld, Germany). Mice were used for experiments when tumor size reached 5 ‑ 10 mm in diameter (3 ‑ 6 weeks after inoculation).

- - 1. Biodistribution

Approximately 2 ‑ 10 MBq (0.20 nmol) of the ^177^Lu-labeled PSMA inhibitors were injected into the tail vein of LNCaP tumor xenograft-bearing male CB17-SCID mice (n = 3 to 5). They were sacrificed by CO_2_ asphyxiation and cervical dislocation either 1 h or 24 h post injection (p.i.) (n = 3 for [^177^Lu]Lu-**5**, -**6** & -**7** (proinhibitor compounds I‑III), n = 4 for [^177^Lu]Lu-**10** (alkyne) and n = 5 for [^177^Lu]Lu-**3** (carbamate I) and n = 5 for [^177^Lu]Lu-**11** (tetrazole)). Selected organs were removed, weighed and organ activities measured in a γ-counter.

***In vivo* data of PSMA inhibitors**

***Table 3***: Biodistribution of [^177^Lu]Lu-PSMA-10 ([^177^Lu]Lu-**1**) and related derivatives with modified inhibitor motifs at 24 h p.i. in male LNCaP tumor xenograft-bearing CB17-SCID mice. Data are expressed as a percentage of the injected dose per gram (% ID/g), mean ± standard deviation (n = 2 for [^177^Lu]Lu-**5***, n = 3 for [^177^Lu]Lu-**6** and [^177^Lu]Lu-**7**, n = 4 for [^177^Lu]Lu-**10** and n = 5 for [^177^Lu]Lu-**1**, [^177^Lu]Lu-**3** and [^177^Lu]Lu-**11**. *Ingestion of radioactively contaminated animal feed led to putative high activities in stomach and intestine, therefore excluding all values of mouse 3).

| Organ | [^177^Lu]Lu-1 ([^177^Lu]Lu-PSMA‑10) | [^177^Lu]Lu-3 (carbamate I) | [^177^Lu]Lu-5 (proinhibitor I) | [^177^Lu]Lu-6 (proinhibitor II) | [^177^Lu]Lu-7 (proinhibitor III) | [^177^Lu]Lu-10 (alkyne) | [^177^Lu]Lu-11 (tetrazole) |
| --- | --- | --- | --- | --- | --- | --- | --- |
| Blood | 0.00 ± 0.00 | 0.00 ± 0.00 | 0.00 ± 0.00 | 0.00 ± 0.00 | 0.01 ± 0.00 | 0.01 ± 0.01 | 0.00 ± 0.00 |
| Heart | 0.02 ± 0.00 | 0.02 ± 0.01 | 0.01 ± 0.00 | 0.03 ± 0.01 | 0.03 ± 0.01 | 0.01 ± 0.00 | 0.02 ± 0.00 |
| Lung | 0.03 ± 0.00 | 0.03 ± 0.01 | 0.33 ± 0.30 | 0.10 ± 0.08 | 0.04 ± 0.01 | 0.03 ± 0.03 | 0.05 ± 0.02 |
| Liver | 0.18 ± 0.06 | 0.13 ± 0.05 | 0.05 ± 0.00 | 0.14 ± 0.02 | 0.17 ± 0.05 | 0.09 ± 0.04 | 0.18 ± 0.04 |
| Spleen | 0.17 ± 0.03 | 0.09 ± 0.01 | 0.05 ± 0.01 | 0.11 ± 0.03 | 0.08 ± 0.02 | 0.04 ± 0.00 | 0.11 ± 0.03 |
| Pancreas | 0.01 ± 0.00 | 0.02 ± 0.01 | 0.01 ± 0.00 | 0.03 ± 0.01 | 0.02 ± 0.00 | 0.01 ± 0.01 | 0.02 ± 0.00 |
| Stomach | 0.06 ± 0.01 | 0.06 ± 0.04 | 0.14 ± 0.07 | 0.05 ± 0.01 | 0.04 ± 0.01 | 0.12 ± 0.11 | 0.12 ± 0.11 |
| Intestine | 0.11 ± 0.05 | 0.12 ± 0.06 | 0.55 ± 0.04 | 0.18 ± 0.06 | 0.10 ± 0.05 | 0.32 ± 0.29 | 0.52 ± 0.68 |
| Kidneys | 1.97 ± 0.78 | 0.31 ± 0.05 | 0.48 ± 0.02 | 2.36 ± 0.39 | 2.04 ± 0.75 | 1.29 ± 0.28 | 3.23 ± 0.77 |
| Adrenals | 0.06 ± 0.04 | 0.04 ± 0.01 | 0.00 ± 0.00 | 0.04 ± 0.00 | 0.03 ± 0.01 | 0.02 ± 0.02 | 0.17 ± 0.19 |
| Muscle | 0.00 ± 0.00 | 0.01 ± 0.01 | 0.00 ± 0.00 | 0.02 ± 0.00 | 0.01 ± 0.00 | 0.00 ± 0.00 | 0.02 ± 0.01 |
| Bone | 0.02 ± 0.01 | 0.02 ± 0.01 | 0.00 ± 0.00 | 0.02 ± 0.01 | 0.04 ± 0.01 | 0.03 ± 0.01 | 0.18 ± 0.04 |
| Tumor | 9.82 ± 0.30 | 1.20 ± 0.55 | 0.09 ± 0.02 | 0.33 ± 0.11 | 0.14 ± 0.05 | 0.10 ± 0.03 | 0.68 ± 0.16 |
| Submandibular gland | 0.04 ± 0.01 | 0.03 ± 0.01 | 0.02 ± 0.01 | 0.07 ± 0.01 | 0.05 ± 0.02 | 0.02 ± 0.00 | 0.04 ± 0.01 |
| Parotid gland | 0.04 ± 0.01 | 0.04 ± 0.02 | 0.02 ± 0.00 | 0.09 ± 0.03 | 0.06 ± 0.01 | 0.02 ± 0.00 | 0.06 ± 0.02 |

***Table 4***: Biodistribution of [^177^Lu]Lu-PSMA-10 ([^177^Lu]Lu-**1**) and related derivatives with modified inhibitor motifs at 1 h p.i. in male LNCaP tumor xenograft-bearing CB17-SCID mice. Data are expressed as a percentage of the injected dose per gram (% ID/g), mean ± standard deviation (n = 5 for [^177^Lu]Lu-**1**, [^177^Lu]Lu-**3** and [^177^Lu]Lu-**11**).

| Organ | [^177^Lu]Lu-1^a^ ([^177^Lu]Lu-PSMA‑10) | [^177^Lu]Lu-3 (carbamate I) | [^177^Lu]Lu-11 (tetrazole) |
| --- | --- | --- | --- |
| Blood | 0.48 ± 0.15 | 0.97 ± 0.18 | 1.09 ± 0.30 |
| Heart | 0.42 ± 0.15 | 0.36 ± 0.04 | 0.41 ± 0.04 |
| Lung | 0.80 ± 0.16 | 2.88 ± 2.96 | 1.23 ± 0.27 |
| Liver | 0.40 ± 0.13 | 0.87 ± 0.28 | 0.74 ± 0.17 |
| Spleen | 10.7 ± 2.9 | 2.51 ± 0.95 | 1.04 ± 0.15 |
| Pancreas | 0.31 ± 0.12 | 0.25 ± 0.06 | 0.33 ± 0.11 |
| Stomach | 0.26 ± 0.08 | 0.30 ± 0.09 | 1.55 ± 1.67 |
| Intestine | 0.28 ± 0.10 | 0.36 ± 0.17 | 0.67 ± 0.21 |
| Kidneys | 173 ± 56 | 61.8 ± 25.9 | 33.2 ± 3.8 |
| Adrenals | 1.30 ± 0.37 | 1.46 ± 0.70 | 0.61 ± 0.42 |
| Muscle | 0.36 ± 0.10 | 0.13 ± 0.04 | 0.15 ± 0.03 |
| Bone | 0.37 ± 0.24 | 0.18 ± 0.02 | 0.47 ± 0.44 |
| Tumor | 12.2 ± 1.8 | 5.31 ± 0.94 | 3.40 ± 0.63 |
| Submandibular gland | n.d. | 0.37 ± 0.08 | 0.40 ± 0.05 |
| Parotid gland | n.d. | 0.62 ± 0.20 | 0.56 ± 0.08 |

^a^Values for [^177^Lu]Lu-PSMA-10 ([^177^Lu]Lu-**1**) at 1 h p.i. were taken from patent WO002019020831A1.^(2)^

***Table 5***: Tumor-to-tissue ratios of [^177^Lu]Lu-PSMA-10 ([^177^Lu]Lu-**1**) and related derivatives with modified inhibitor motifs at 24 h p.i. in male LNCaP tumor xenograft-bearing CB17-SCID mice. Data are expressed as mean ratios ± standard deviation (n = 4 for [^177^Lu]Lu-**10** and n = 5 for [^177^Lu]Lu-**1**, [^177^Lu]Lu-**3** and [^177^Lu]Lu-**11**).

| Ratio  Tumor-to- | [^177^Lu]Lu-1 ([^177^Lu]Lu-PSMA‑10) | [^177^Lu]Lu-3 (carbamate I) | [^177^Lu]Lu-10 (alkyne) | [^177^Lu]Lu-11 (tetrazole) |
| --- | --- | --- | --- | --- |
| blood | 11498 ± 1953 | 947 ± 652 | 26.8 ± 16.9 | 227 ± 50 |
| kidney | 5.66 ± 1.99 | 3.80 ± 1.36 | 0.08 ± 0.01 | 0.22 ± 0.06 |
| muscle | 2441 ± 373 | 127 ± 92 | 27.7 ±10.9 | 55.9 ± 24.3 |
| liver | 57.5 ± 13.1 | 8.94 ± 2.94 | 1.46 ± 0.88 | 3.74 ± 0.50 |
| submandibular gland | 275 ± 49 | 34.5 ± 9.2 | 6.11 ± 1.78 | 16.9 ± 2.2 |
| parotid gland | 250 ± 64 | 31.8 ± 9.3 | 6.38 ± 3.06 | 12.2 ± 1.7 |

***Table 6***: Tumor-to-tissue ratios of [^177^Lu]Lu-**3** (carbamate I) and [^177^Lu]Lu-**11** (tetrazole) at 1 h p.i. in male LNCaP tumor xenograft‑bearing CB17-SCID mice. Data are expressed as mean ratios ± standard deviation (n = 5 for [^177^Lu]Lu-**3** and [^177^Lu]Lu-**11**).

| Ratio  Tumor-to- | [^177^Lu]Lu-3 (carbamate I) | [^177^Lu]Lu-11 (tetrazole) |
| --- | --- | --- |
| blood | 5.57 ± 1.18 | 3.46 ± 1.62 |
| kidney | 0.09 ± 0.03 | 0.10 ± 0.03 |
| muscle | 44.1 ± 9.0 | 24.9 ± 10.2 |
| liver | 6.61 ± 2.19 | 5.00 ± 2.11 |
| submandibular gland | 14.7 ± 3.5 | 8.74 ± 2.52 |
| parotid gland | 9.09 ± 3.13 | 6.20 ± 1.74 |

- - 1. Metabolite analysis

[^177^Lu]Lu-**11** (9.64 MBq) was injected into the tail vein of a LNCaP tumor xenograft-bearing CB17-SCID mouse. The animal was sacrificed 1 h p.i. and subjected to the standard procedure for biodistribution studies. In addition, urine was taken from all mice that were investigated in this experiment (8.4 ‑ 9.0 MBq) and pooled (n = 5). Relevant dissected organs (tumor, kidneys, liver) and body fluids (blood and urine) were collected, homogenized if necessary and subjected to mechanochemical as well as solid phase extraction (SPE).

Four 2 mL LoBind tubes were equipped with steel and ceramic beads from Lysis Tubes W (analytikjena, Jena, Germany) for kidneys, tumor and liver (bisected). 1 mL radioimmunoprecipitation assay (RIPA) buffer containing 2.0 µmol of 2‑PMPA was added to each tube and the organs were homogenized with a MM‑400 ball mill (Retsch GmbH, Haan, Germany) at 30 Hz for 20 min. Afterwards, the homogenates were transferred to LoBind tubes free from steel and ceramic beads and centrifuged (15,200 rpm, 10 min, 21 °C). The first supernatants were stored, and the precipitates were again subjected to mechanochemical extraction with 1 mL RIPA buffer (2.0 µmol PMPA) at 30 Hz for 20 min. After centrifugation (15,200 rpm, 10 min, 21 °C), also the second supernatants were kept for SPE.

After cardiac puncture, 1 mL Tracepur^®^-H_2_O was directly added to the collected blood sample and centrifuged twice (13,000 rpm, 5 min) to separate the plasma from the blood cells. The precipitate was dissolved in 500 µL Tracepur^®^-H_2_O and together with the first supernatant again centrifuged (13,000 rpm, 5 min). Both supernatants were kept for SPE.

Pooled urine samples were centrifuged (13,000 rpm, 5 min) and the supernatant was directly analyzed via radio‑RP‑HPLC.

For solid phase extraction, the supernatants were loaded onto Strata-X cartridges (200 mg), which were preconditioned with 5 mL MeOH and 5 mL H_2_O (eight cartridges in total, for all organ and blood supernatants). The cartridges were washed with 1 mL Tracepur^®^-H_2_O and dried prior to elution. For each cartridge, 750 µL of MeCN/H_2_O (6/4, 0.1% TFA) were used for elution and the extracts were analyzed via radio‑RP-HPLC. The extraction efficiencies were calculated before and after SPE and the results are given in *Table 7*.

***Table 7***: Extraction efficiencies of [^177^Lu]Lu-**11** from liver, tumor, kidneys and blood using a MM‑400 ball mill. The percentage of activity after sample extraction and after SPE purification was quantified, decay corrected and the overall extracted activity was calculated.

| Organs and body fluids | efficiency [% extracted radioactivity] | | |
| --- | --- | --- | --- |
|  | **sample extraction** | **SPE purification** | **overall** |
| liver | 96.1 | 67.2 | 64.5 |
| tumor | 97.3 | 76.1 | 74.0 |
| kidneys | 96.0 | 74.8 | 71.8 |
| blood | 94.8 | 78.9 | 74.8 |


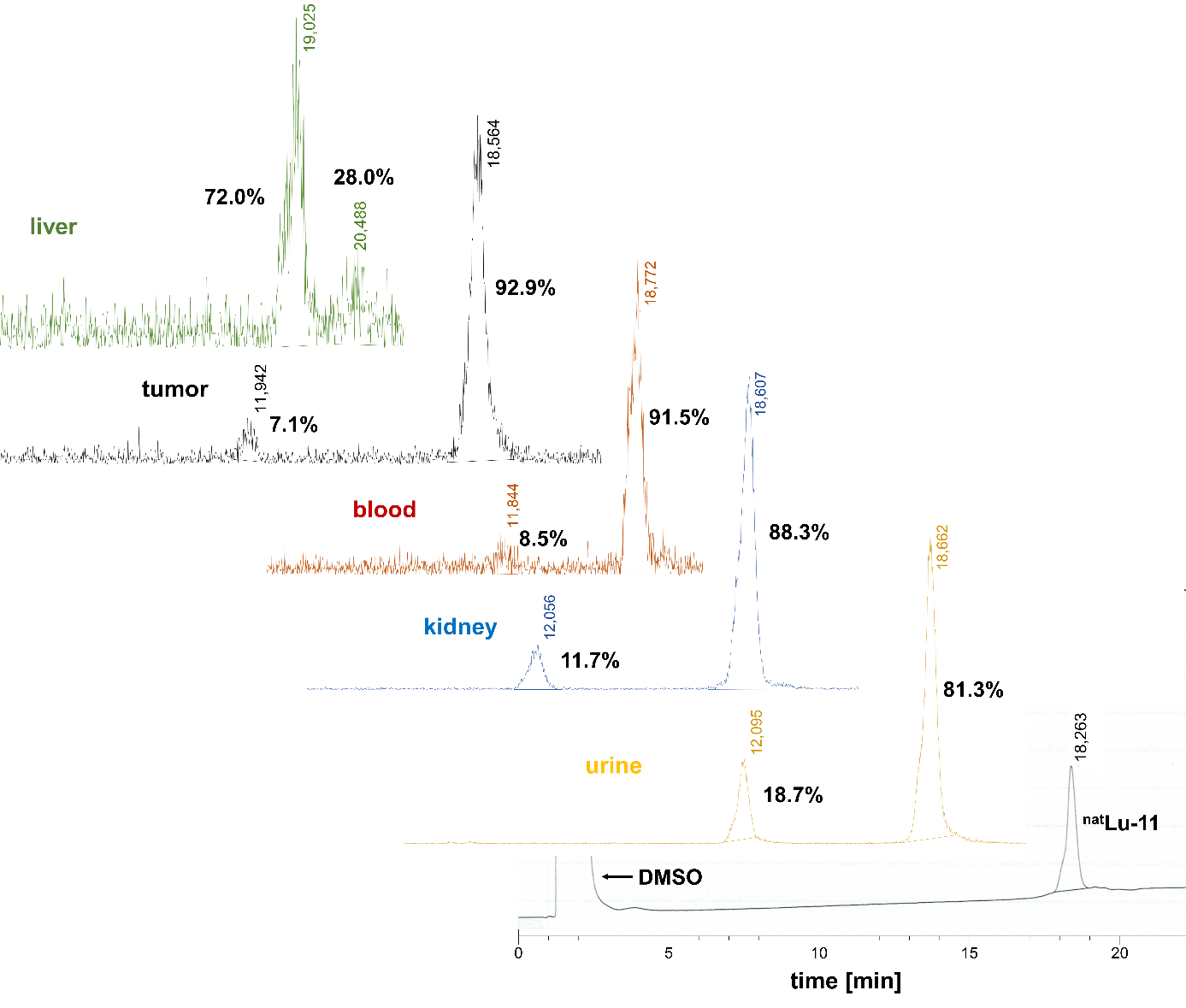


***Figure 1***: Radio-RP‑HPLC analyses of extracts from homogenized organs and body fluids of tumor xenograft‑bearing CB17‑SCID mice, 1 h p.i. of [^177^Lu]Lu-**11** (9.64 MBq, gradient: 25 - 40% MeCN (0.1% TFA) in 20 min, flow rate: 1 mL/min). The retention time (18.3 min) of the intact cold standard (^nat^-Lu-**11**) was previously determined and hence, served as a reference. The radioactivity detector was placed downstream of the UV‑detector causing for a slight time delay of the radioactivity signals.

- - 1. *μ*SPECT/CT imaging

Imaging experiments were conducted using a MILabs VECTor^4^ small-animal SPECT/PET/OI/CT. The resulting data were analyzed by the associated PMOD (version 4.0) software. Mice were anaesthetized with isoflurane and the ^177^Lu-labeled PSMA compounds were injected via the tail vein. Mice were euthanized 1 h or 24 h p.i. and blood samples for later biodistribution or metabolite analysis were taken by cardiac puncture before image acquisition. Static images were acquired with 45 min acquisition time using the HE-GP-RM collimator and a step-wise multi‑planar bed movement. All images were reconstructed using the MILabs‑Rec software (version 10.02) and a pixel-based Similarity-Regulated Ordered Subsets Expectation Maximization (SROSEM) algorithm with a window-based scatter correction (20% below and 20% above the photopeak, respectively). Voxel size CT: 80 µm, voxel size SPECT: 0.8 mm, 1.6 mm (FWHM) Gaussian blurring post processing filter, with calibration factor in kBq/mL and decay correction, no attenuation correction.

1. ABBREVIATIONS

2-Aha 2-aminoheptanoic acid

2-Aoc 2-aminooctanoic acid

2-CT 2-chlorotrityl

2-PMPA 2-(phosphonomethyl)pentane-1,5‑dioic acid

A_m_ molar activity

BOP (benzotriazol-1-yloxy)tris(dimethylamino)phosphonium hexafluorophosphate

BSA bovine serum albumin

Bzl benzyl

CAN cerium(IV) ammonium nitrate

Cbz carbobenzoxy

CDI 1,1’‑carbonyldiimidazole

CT computed tomography

Dap 2,3-diaminopropionic acid

DCE 1,2-dichloroethane

DCM dichloromethane

Dde *N*-1-(4,4-dimethyl-2,6-dioxocyclohex-1-ylidene)ethylamine

DIAD diisopropyl azodicarboxylate

DIPEA *N*,*N*-diisopropylethylamine

DMAP 4-dimethylaminopyridine

DMEM Dulbecco's Modified Eagle's Medium

DMF dimethylformamide

DMSO dimethyl sulfoxide

DOTA 1,4,7,10-tetraazacyclododecane-1,4,7,10-tetraacetic acid

EDC *N*-(3-dimethylaminopropyl)-*N*’-ethylcarbodiimide

FWHM full width at half maximum

HBSS Hank’s buffered salt solution

HFIP 1,1,1,3,3,3-hexafluoro-2-propanol

HOAt 1-hydroxy-7-azabenzotriazole

IC_50_ half maximal inhibitory concentration

ID/g injected dose per gram

MeCN acetonitrile

MeOH methanol

NHS *N*-hydroxysuccinimide

NMP *N*-methyl-2-pyrrolidone

OI optical imaging

PBS phosphate-buffered saline

PET positron emission tomography

PPh_3_ triphenylphosphane

PSMA prostate-specific membrane antigen

RCP radiochemical purity

RCY radiochemical yield

RIPA radioimmunoprecipitation assay

RP‑HPLC reversed-phase high-performance liquid chromatography

r.t. room temperature

SiFA-BA silicon-based fluoride acceptor-benzoic acid

SPECT single-photon emission computed tomography

TBTU 2-(1*H*-benzotriazole-1-yl)-1,1,3,3-tetramethylaminium tetrafluoroborate

*t*Bu *tert*-butyl

*t*BuOH *tert*-butanol

TEA triethylamine

TFA trifluoroacetic acid

TIPS triisopropylsilane

TLC thin layer chromatography

TMSN_3_ trimethylsilyl azide

1. REFERENCES

1. Wurzer A, Di Carlo D, Schmidt A, Beck R, Eiber M, Schwaiger M, et al. Radiohybrid Ligands: A Novel Tracer Concept Exemplified by 18F- or 68Ga-Labeled rhPSMA Inhibitors. Journal of Nuclear Medicine. 2020;61(5):735-42.

2. Wurzer AJ, Wester H-J, Eiber MJ, inventors; Technische Universitaet Muenchen, Germany; Technische Universitaet Muenchen - Klinikum Rechts der Isar . assignee. Preparation of peptides as dual mode radiotracers and therapeutics patent WO2019020831A1. 2019.

3. Zhang D-W, Luo Z, Liu G-J, Weng L-H. α N-O turn induced by fluorinated α-aminoxy diamide: synthesis and conformational studies. Tetrahedron. 2009;65(48):9997-10001.

4. Bergmeier SC, Cobas AA, Rapoport H. Chirospecific synthesis of (1S,3R)-1-amino-3-(hydroxymethyl)cyclopentane, precursor for carbocyclic nucleoside synthesis. Dieckmann cyclization with an α-amino acid. J Org Chem. 1993;58(9):2369-76.

5. Yang X, Mease RC, Pullambhatla M, Lisok A, Chen Y, Foss CA, et al. [18F]Fluorobenzoyllysinepentanedioic Acid Carbamates: New Scaffolds for Positron Emission Tomography (PET) Imaging of Prostate-Specific Membrane Antigen (PSMA). Journal of Medicinal Chemistry. 2016;59(1):206-18.

6. Weineisen M, Simecek J, Schottelius M, Schwaiger M, Wester H-J. Synthesis and preclinical evaluation of DOTAGA-conjugated PSMA ligands for functional imaging and endoradiotherapy of prostate cancer. EJNMMI Research. 2014;4(1):63.

7. Shin I, Lee M-r, Lee J, Jung M, Lee W, Yoon J. Synthesis of Optically Active Phthaloyl d-Aminooxy Acids from l-Amino Acids or l-Hydroxy Acids as Building Blocks for the Preparation of Aminooxy Peptides. The Journal of Organic Chemistry. 2000;65(22):7667-75.

8. Hyun S-H, Kim H-k, Kim J-M, Thompson DH. Oriented Insertion of phi29 N-Hexahistidine-tagged gp10 Connector Protein Assemblies into C20BAS Bolalipid Membrane Vesicles. J Am Chem Soc. 2010;132(48):17053-5.

9. Kozikowski AP, Zhang J, Nan F, Petukhov PA, Grajkowska E, Wroblewski JT, et al. Synthesis of Urea-Based Inhibitors as Active Site Probes of Glutamate Carboxypeptidase II:  Efficacy as Analgesic Agents. Journal of Medicinal Chemistry. 2004;47(7):1729-38.
